# The Global Burden of Motor Neuron Disease: An Analysis of the 2019 Global Burden of Disease Study

**DOI:** 10.3389/fneur.2022.864339

**Published:** 2022-04-21

**Authors:** Jin Park, Jee-Eun Kim, Tae-Jin Song

**Affiliations:** Department of Neurology, Seoul Hospital Ewha Womans University College of Medicine, Seoul, South Korea

**Keywords:** motor neuron disease, amyotrophic lateral sclerosis, incidence, prevalence, disease burden

## Abstract

Up-to-date, accurate information on the disease burden of motor neuron disease (MND) is the cornerstone for evidence-based resource allocation and healthcare planning. We aimed to estimate the burden of MND globally from 1990 to 2019, as part of the Global Burden of Disease, Injuries and Risk Factor (GBD) study. Amyotrophic lateral sclerosis, progressive muscular atrophy, primary lateral sclerosis, pseudobulbar palsy, spinal muscular atrophy and hereditary spastic paraplegia- were included for analysis as MNDs. We measured age-standardized incidence, prevalence, death, and disability-adjusted life-years (DALYs) in 204 countries and territories worldwide from 1990 to 2019 using spatial Bayesian analyses. The effects of age, sex, and the sociodemographic index (measures of income per capita, education, and fertility) on incidence, prevalence, death, and disability-adjusted life-years due to MNDs were explored. According to 2019 GBD estimates, there were ~268,673 [95% uncertainty interval (UI), 213,893–310,663] prevalent cases and 63,700 (95% UI, 57,295–71,343) incident cases of MND worldwide. In 2019, MND caused 1,034,606 (95% UI, 979,910–1,085,401) DALYs and 39,081 (95% UI, 36,566–41,129) deaths worldwide. The age-standardized rates of prevalence, incidence, death, and DALYs for MNDs in 2019 were 3.37 (95% UI, 2.9–3.87) per 100,000 people, 0.79 (95% UI, 0.72–0.88) per 100,000 people, 0.48 (95% UI, 0.45–0.51) per 100,000 people, and 12.66 (95% UI, 11.98–13.29) per 100,000 people, respectively. The global prevalence and deaths due to MND in 2019 were increased (1.91% [95% UI, 0.61–3.42] and 12.39% [95% UI, 5.81–19.27], respectively) compared to 1990, without significant change in incidence. More than half of the prevalence and deaths due to MND occurred in three high-income regions (North America, Western Europe, and Australasia). In most cases, the prevalence, incidence, and DALYs of MNDs were high in regions with high sociodemographic index; however, in high-income East Asia, these were relatively low compared to similar sociodemographic index groups elsewhere. The burden of MND increased between 1990 and 2019. Its expected increase in the future highlights the importance of global and national healthcare planning using more objective evidence. Geographical heterogeneity in the MND burden might suggest the influences of sociodemographic status and genetic background in various regions.

## Introduction

Motor neuron diseases (MNDs) are rare neurological disease groups of neurodegenerative disorders associated with the degeneration of motor neurons in the upper and lower extremities (1). They include amyotrophic lateral sclerosis (ALS), primary lateral sclerosis, hereditary spastic paraplegia, progressive muscular atrophy, spinal muscular atrophy, and pseudobulbar palsy (2). Among MNDs, ALS—the most common disease entity—causes respiratory failure in 50% of patients within 2 years of diagnosis. Other MNDs also have poor long-term prognoses, imposing a socioeconomic burden on patients and care givers (1, 2).

Although epidemiologic studies on MNDs have been published in the United States and Europe, their incidence, prevalence, and burden are not well known because the diseases are rare (3–6). Although it varies according to age, sex, and region, the peak incidence of ALS is at ~70 years, the incidence rate is 1.7 per 100,000 person-years, and the prevalence is 4.5 per 100,000 people (7, 8). Moreover, according to the 2016 Global Burden of Diseases (GBD) estimates, the incidence rate of all age was 0.78 per 100 000 person-years for MNDs (9). In addition, the age-standardized prevalence was high in high-income Europe, Australasia, and North America, excluding the Asia-Pacific region (9). Despite this high prevalence, there are few recent global epidemiologic studies of MNDs.

The purpose of this study was to investigate the global burden of MNDs, including the incidence, prevalence, death, disability-adjusted life-years (DALYs), years lived with disability (YLDs), and years of life lost (YLLs) between 1990 and 2019 from GBD information according to age, sex, regions, and the estimates from individual countries. Furthermore, we investigated the GBD of MNDs based on the sociodemographic index (SDI) reflecting the development of each country.

## Methods

### Overview

The GBD Study is a systematic and comprehensive study of diseases worldwide. Based on the estimates of this study, it is possible to compare and analyze the current status of the global, regional, and national burden of diseases (10). The GBD Study complies with the Guidelines for Accurate and Transparent Health Estimates Reporting statement (11). Based on the Institute for Health Metrics and Evaluation (IHME) of the University of Washington, which is in charge of the GBD Study, only anonymous information is used in the GBD Study, resulting in the waiver of informed consent.

Our study used the estimates from the GBD's public website. All the results related to GBD research on MNDs can be freely accessed and downloaded from the GBD Compare website and the Global Health Data Exchange website (GBD Compare, available at: https://vizhub.healthdata.org/gbd-compare/; Global Health Data Exchange, available at: http://ghdx.healthdata.org/) (10). GBD 2019 methods are described in detail on the GBD website and in a previous study (12). The GBD 2019 is a multinational collaborative study conducted by worldwide countries that is updated every year. The most recent version provides the burden of diseases according to age, sex, and region (369 diseases and injuries in 204 countries and territories) from 1990 to 2019. The estimates acquisition and analysis of our study followed the methodology detailed on the GBD website. Our dataset from 1990 to 2019 for MNDs was provided on the GBD website, and the estimates were extracted from the GBD standards. For comparison of the temporal change by region and country, the variations of the estimates were presented as the percentage change in age-standardized rates between 1990 and 2019.

### Case Definition

MNDs are a set of chronic, degenerative, and progressive neurological conditions typified by the destruction of motor neurons and the subsequent deterioration of voluntary muscle activity. The most common MND is ALS. The International Statistical Classification of Diseases and Related Health Problems (ICD)-10 code corresponding to MNDs is G12. The GBD Study's gold standard diagnostic criteria are the El Escorial Criteria, combined with other similar criteria (e.g., the original set from World Federation of Neurology) if necessary (9, 13, 14).

### Search Terms

Detailed methods for obtaining information for nonfatal estimates and death have been described in previous research (12). Considering DALYs, YLDs, and YLLs, these estimates for MNDs were acquired from surveillance systems of diseases, registries, survey microdata, health claims data, and systematic reviews of published and unpublished reports (12). The IHME searched PubMed, Medline, CINAHL®, Embase, World Health Organization Library Information System, CAP abstracts, and System for Information on Gray Literature in Europe databases for Global Burden of Disease Study data, regardless of language, age, and sex. The terms, “motor neuron disease,” “amyotrophic lateral sclerosis,” “primary lateral sclerosis,” “spinal muscular atrophy,” “progressive muscular atrophy,” and “pseudobulbar palsy” were searched individually. These terms were re-searched with combinations of the following terms, “epidemiology,” “population sample,” “population study,” “population-based,” “cross-sectional,” “cross sectional,” “prevalen^*^,” and “inciden^*^.” (12) Systematic reviews from the above data sources and the National Health Interview Survey, National Health and Nutrition Examination Survey in United States and other nationwide claim data were reviewed for the GBD Study (12, 15). The studies or dataset complied with small sample size (<150), review article, not a population sample study, studies in which the subpopulation of the national population was not clearly explained were excluded by IHME (12, 15). These datasets are repositioned to the Global Health Data Exchange, and data of different characteristics are analyzed using DisMod-MR 2.1, a Bayesian meta-regression tool (16). All rates were age-standardized using the GBD standard. Data were described using 95% uncertainty intervals (UIs) and changes from 1990 to 2019 as percentages (95% UIs) provided by the GBD website.

### Sociodemographic Index

The SDI was used to investigate the association of the level of development of regions or countries with the GBD of MNDs (12). The SDI is a composite indicator that measures the development of individual countries. It is defined as 0 in the lowest case and 1 in the highest case and calculated based on the lag-distributed income per capita, the total fertility rate for those under 25, and the average educational level of the population over the age of 15 (17). In our study, the age-standardized prevalence and DALYs for each region and DALYs for each country were estimated according to the SDI.

## Results

### 2019 GBD of Motor Neuron Diseases by Region

**Figure 1:**
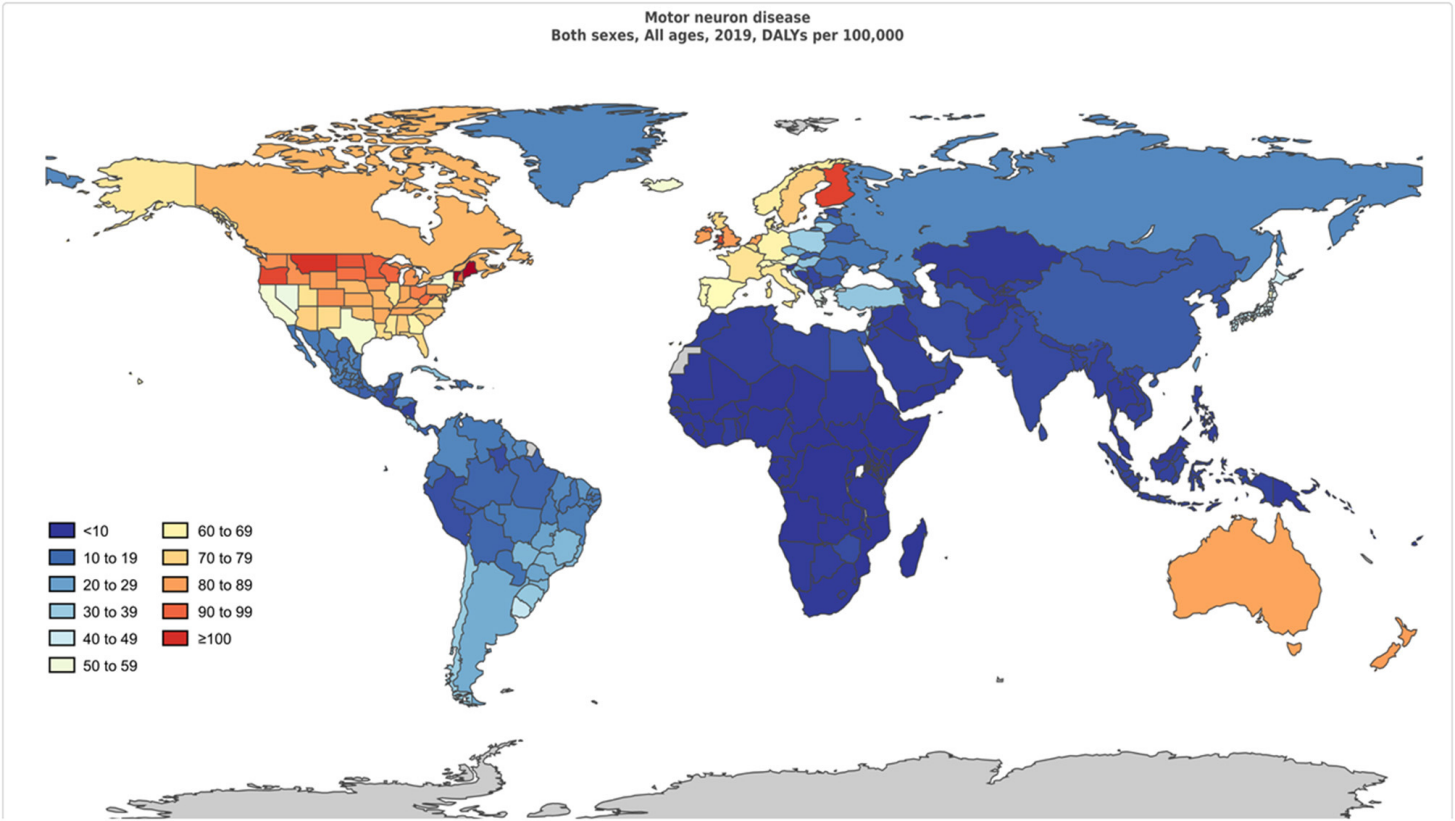
Age-standardized disability-adjusted life-years per 100,000 population of motor neuron diseases by region for both sexes, 2019.

Prevalence, incidence, DALYs, YLDs, YLLs, and death due to MNDs in counts and age-standardized rates for both sexes for 2019 are listed in Table 1. The age-standardized rates of DALYs of MNDs in the 21 GBD world regions generally increased with SDI (Figure 1).

**Table 1:** Prevalence, incidence, DALYs, YLD, YLL, and death of motor neuron diseases in counts and age-standardized rate for both sexes combined in 1990 and 2019, with percentage change between 1990 and 2019 by GBD region.

	**1990**		**2019**		**Percentage change in age-standardized rates between 1990 and 2019 (%)**
	**Counts (95% UI)**	**Age-standardized rate (per 100k)**	**Counts (95% UI)**	**Age-standardized rate (per 100k)**	
**Prevalence**					
Global	159074.07 (134173.93, 187017.72)	3.31 (2.83, 3.85)	268673.82 (231893.92, 310663.85)	3.37 (2.90, 3.87)	1.91 (0.61, 3.42)
East Asia	30416.42 (24265.40, 37552.45)	2.47 (2, 3.02)	43368.36 (35365.62, 53087.77)	2.81 (2.28, 3.42)	14.01 (11.92, 16.55)
Southeast Asia	6976.22 (5524.27, 8766.2)	1.56 (1.26, 1.90)	11553.01 (9320.49, 14150.96)	1.70 (1.37, 2.07)	8.81 (7.37, 10.41)
Central Asia	1763.87 (1427.23, 2136.48)	2.61 (2.15, 3.16)	2482.75 (2010.03, 3034.69)	2.66 (2.17, 3.22)	1.81 (−0.08, 3.63)
High-income Asia Pacific	8184.62 (6975.14, 9505.28)	4.37 (3.71, 5.10)	13685.11 (11728.72, 15889.95)	4.96 (4.21, 5.73)	13.50 (10.20, 17.06)
South Asia	16889.59 (13251.61, 21327.56)	1.60 (1.28, 2.01)	32423.68 (25648.91, 40699.06)	1.77 (1.41, 2.21)	10.32 (8.69, 12.11)
Central Europe	4732.18 (3963.39, 5604.84)	3.78 (3.13, 4.46)	5114.87 (4353.92, 6023.38)	4.16 (3.46, 4.91)	10.11 (7.77, 12.54)
Eastern Europe	7394.11 (6051.33, 8952.39)	3.28 (2.67, 3.97)	7226.55 (6019.61, 8647.80)	3.43 (2.81, 4.11)	4.31 (2.55, 6.30)
Western Europe	32546.71 (28367.54, 37168.89)	6.65 (5.83, 7.62)	56841.52 (48862.68, 64813.67)	8.33 (7.24, 9.54)	25.29 (22.14, 28.44)
Southern Latin America	1903.01 (1608.70, 2235.20)	3.90 (3.31, 4.57)	3446.54 (2961.42, 3967.82)	4.77 (4.08, 5.53)	22.33 (17.63, 26.30)
High-income North America	24268.48 (21294.92, 27403.77)	7.69 (6.74, 8.72)	43939.61 (40591.11, 47456.48)	8.86 (8.19, 9.51)	15.16 (6.95, 25.60)
Andean Latin America	618.51 (499.81, 765.97)	1.76 (1.45, 2.12)	1195.76 (986.3, 1444.28)	1.90 (1.57, 2.29)	8.13 (4.99, 10.95)
Central Latin America	3582.30 (2860.72, 4423.03)	2.28 (1.86, 2.76)	6259.06 (5183.23, 7537.21)	2.49 (2.06, 2.99)	9.41 (7.15, 12.11)
Tropical Latin America	3578.71 (2864.26, 4373.53)	2.43 (1.99, 2.92)	6577.87 (5550.97, 7804.33)	2.84 (2.39, 3.37)	16.94 (13.06, 21.43)
North Africa and Middle East	7659.13 (6139.90, 9397.60)	2.49 (2.03, 3.01)	15573.91 (12657.01, 19025.23)	2.58 (2.11, 3.10)	3.55 (2.01, 5.10)
Central Sub-Saharan Africa	640.30 (508.60, 805.50)	1.41 (1.14, 1.74)	1574.70 (1246.39, 1974.15)	1.42 (1.16, 1.73)	0.75 (−1.69, 3.26)
Eastern Sub-Saharan Africa	2258.65 (1776.01, 2848.39)	1.44 (1.16, 1.77)	5243.76 (4117.62, 6614.97)	1.49 (1.21, 1.83)	3.86 (2.86, 4.92)
Southern Sub-Saharan Africa	902.47 (715.56, 1136.22)	1.81 (1.47, 2.22)	1455.91 (1164.75, 1808.32)	1.89 (1.53, 2.32)	4.45 (3.13, 5.94)
Western Sub-Saharan Africa	2386.40 (1875.05, 3002.83)	1.44 (1.16, 1.77)	5937.72 (4650.94, 7517.55)	1.49 (1.19, 1.83)	3.42 (2.58, 4.23)
Oceania	86.03 (68.61, 107.11)	1.43 (1.17, 1.73)	175.43 (139.94, 217.28)	1.40 (1.14, 1.69)	−1.95 (−4.69, 0.84)
Australasia	1426.95 (1245.42, 1648.03)	6.35 (5.57, 7.32)	3341.02 (2886.63, 3856.43)	8.03 (6.99, 9.18)	26.40 (19.56, 33.03)
Caribbean	859.40 (714.72, 1029.32)	2.53 (2.14, 3.02)	1256.66 (1070.71, 1478.73)	2.59 (2.20, 3.04)	2.31 (−0.17, 4.80)
**Incidence**					
Global	35589.21 (31621.30, 40068.04)	0.79 (0.71, 0.89)	63700.04 (57295.90, 71343.33)	0.79 (0.72, 0.88)	0.28 (−0.37, 0.94)
East Asia	6278.11 (5218.62, 7576.54)	0.59 (0.50, 0.72)	9432.66 (7816.94, 11699.79)	0.54 (0.46, 0.65)	−9.02 (−10.92,−6.96)
Southeast Asia	1475.31 (1218.21, 1780.07)	0.41 (0.34, 0.50)	2610.84 (2121.40, 3208.19)	0.40 (0.33, 0.49)	−1.44 (−2.49,−0.44)
Central Asia	303.68 (255.62, 359.75)	0.49 (0.42, 0.60)	431.58 (355.63, 525.64)	0.49 (0.41, 0.59)	−0.63 (−2.04, 0.91)
High-income Asia Pacific	1546.95 (1383.49, 1718.90)	0.81 (0.72, 0.89)	3114.27 (2840.71, 3436.45)	0.87 (0.78, 0.97)	8.26 (5.65, 11.08)
South Asia	3903.90 (3204.06, 4756.93)	0.42 (0.35, 0.52)	6855.22 (5615.30, 8401.31)	0.42 (0.34, 0.51)	−1.92 (−3.40,−0.29)
Central Europe	826.68 (716.85, 956.76)	0.63 (0.55, 0.72)	1098.89 (968.04, 1253.9)	0.69 (0.61, 0.79)	10.29 (8.12, 12.69)
Eastern Europe	1239.63 (1035.78, 1495.5)	0.52 (0.44, 0.62)	1499.10 (1284.01, 1760.28)	0.58 (0.50, 0.68)	11.46 (8.25, 14.71)
Western Europe	7787.23 (7325.06, 8273.72)	1.55 (1.45, 1.64)	13796.97 (13037.65, 14494.46)	1.85 (1.73, 1.95)	19.51 (17.68, 21.37)
Southern Latin America	402.63 (355.60, 449.83)	0.84 (0.75, 0.94)	734.86 (664.39, 806.44)	0.96 (0.87, 1.06)	14.01 (10.52, 17.64)
High-income North America	5685.47 (5372.57, 6019.86)	1.75 (1.64, 1.85)	11322.79 (10817.90, 11839.31)	1.97 (1.88, 2.06)	12.83 (9.85, 16.22)
Andean Latin America	125.78 (104.87, 147.14)	0.42 (0.35, 0.49)	268.04 (228.69, 312.01)	0.45 (0.38, 0.52)	7.84 (5.06, 10.82)
Central Latin America	704.93 (595.06, 814.16)	0.51 (0.44, 0.59)	1414.86 (1230.26, 1621.14)	0.59 (0.51, 0.67)	15.36 (12.06, 18.73)
Tropical Latin America	779.70 (672.64, 897.89)	0.62 (0.54, 0.71)	1915.61 (1705.55, 2132.61)	0.83 (0.74, 0.91)	32.92 (27.11, 38.88)
North Africa and Middle East	1862.19 (1577.27, 2168.99)	0.61 (0.52, 0.72)	3409.38 (2880.66, 4056.06)	0.62 (0.53, 0.73)	1.92 (0.29, 3.56)
Central Sub-Saharan Africa	225.47 (183.85, 275.70)	0.62 (0.51, 0.78)	539.21 (437.93, 660.28)	0.64 (0.53, 0.81)	3.20 (0.55, 5.66)
Eastern Sub-Saharan Africa	812.17 (667.31, 988.29)	0.67 (0.55, 0.84)	1735.36 (1424.63, 2131.85)	0.66 (0.55, 0.83)	−0.89 (−1.78,−0.03)
Southern Sub-Saharan Africa	213.43 (178.65, 257.52)	0.53 (0.44, 0.65)	376.93 (311.48, 465.51)	0.55 (0.46, 0.68)	3.38 (1.97, 4.86)
Western Sub-Saharan Africa	721.11 (593.72, 867.07)	0.51 (0.42, 0.63)	1634.14 (1349.80, 1970.13)	0.51 (0.42, 0.63)	0.01 (−0.78, 0.78)
Oceania	21.21 (17.65, 25.04)	0.45 (0.38, 0.53)	45.65 (37.71, 54.73)	0.43 (0.36, 0.52)	−3.05 (−5.39,−0.43)
Australasia	471.97 (449.22, 495.56)	2.09 (1.99, 2.19)	1108.53 (1056.79, 1154.97)	2.47 (2.36, 2.58)	18.42 (15.85, 20.95)
Caribbean	201.66 (175.29, 229.01)	0.66 (0.57, 0.75)	355.13 (314.48, 400.05)	0.73 (0.64, 0.81)	10.40 (8, 12.89)
**DALYs**					
Global	624364.36 (594254.18, 665295.3)	13.20 (12.70, 13.92)	1034606.59 (979910.92, 1085401.11)	12.66 (11.98, 13.29)	−4.50 (−10.09, 1.87)
East Asia	152285.99 (135032.40, 170843.37)	13.01 (11.54, 14.55)	113080.73 97726.25, 128867.40)	6.83 (6.05, 7.70)	−47.53 (−55.99,−37.57)
Southeast Asia	11458.45 (10039.59, 13066.93)	3.09 (2.69, 3.48)	20690.9 (17170.16, 24504.33)	3.01 (2.51, 3.56)	−2.64 (−18.56, 13.88)
Central Asia	1187.31 (1007.70, 1426.9)	1.98 (1.69, 2.40)	2207.82 (1926.05, 2531.33)	2.40 (2.10, 2.75)	21.56 (0.77, 40.61)
High-income Asia Pacific	36131.48 (34218.25, 38298.73)	21.06 (19.40, 23.14)	53490.14 (48237.91, 57800.70)	14.66 (13.45, 15.74)	−30.35 (−38.57,−22.21)
South Asia	27013.59 (20867.31, 33597.14)	3.08 (2.28, 4.04)	72624.17 (58024.33, 88717.91)	4.49 (3.57, 5.51)	45.83 (18.05, 78.76)
Central Europe	16722.43 (16036.79, 17499.34)	14.08 (13.39, 14.90)	22124.33 (19284.68, 25046.48)	14.33 (12.37, 16.36)	1.82 (−11.75, 16.63)
Eastern Europe	11952.50 (10375.70, 15679.64)	4.85 (4.25, 6.26)	30570.79 (27335.72, 33943.33)	10.69 (9.64, 11.77)	120.46 (66.55, 162.88)
Western Europe	169362.09 (165230.78, 173320.48)	36.52 (35.58, 37.51)	283150.27 (262769.68, 301431.82)	39.39 (36.60, 41.89)	7.86 (0.25, 15.15)
Southern Latin America	3956.35 (3724.62, 4193.44)	8.26 (7.78, 8.75)	17187.38 (15694.86, 18385.60)	22.30 (20.38, 23.94)	170.04 (145.72, 193.32)
High-income North America	115678.28 (112284.79, 118971.11)	37.32 (36.20, 38.44)	252573.77 (242925.55, 260048.76)	46.91 (45.25, 48.37)	25.69 (21.4, 29.91)
Andean Latin America	1624.72 (1433.85, 1823.69)	5.81 (5.16, 6.57)	4723.74 (3757.67, 5888.11)	8.02 (6.38, 9.96)	38.04 (8.38, 75.25)
Central Latin America	9302.06 (8828.52, 9825.84)	7.35 (7.07, 7.66)	31333.12 (26157.22, 36995.01)	12.74 (10.62, 15.02)	73.35 (43.60, 105.07)
Tropical Latin America	14370.77 (13490.43, 15459.08)	11.51 (10.93, 12.2)	44751.24 (40955.11, 47744.19)	18.58 (16.98, 19.87)	61.43 (40.45, 77.07)
North Africa and Middle East	33985.19 (22089.94, 57107.66)	9.40 (7.06, 13.85)	41628.31 (33815.43, 50605.43)	7.84 (6.36, 9.54)	−16.63 (−44.77, 17.91)
Central Sub-Saharan Africa	503.65 (390.60, 696.80)	1.05 (0.80, 1.54)	1065.5 (847.45, 1333.19)	0.98 (0.76, 1.27)	−6.01 (−28.77, 17.51)
Eastern Sub-Saharan Africa	1739.75 (1403.49, 2223.76)	1.02 (0.77, 1.44)	3725.92 (3107.29, 4408.37)	1.02 (0.81, 1.25)	−0.41 (−18.27, 15.35)
Southern Sub-Saharan Africa	1021.90 (888.99, 1161.36)	2.64 (2.26, 3.02)	1687.40 (1392.86, 2091.32)	2.40 (1.99, 2.98)	−9.14 (−28.18, 19.85)
Western Sub-Saharan Africa	3411.76 (2655.83, 4296.15)	2.57 (1.92, 3.30)	6169.26 (5024.46, 7541.74)	1.95 (1.59, 2.41)	−23.98 (−44.73, 2.11)
Oceania	182.01 (129.88, 246.33)	4.49 (3.11, 6.21)	256.61 (187.75, 348.56)	2.60 (1.86, 3.60)	−42.14 (−54.72,−26.07)
Australasia	10073.49 (9630.45, 10508.85)	46.16 (43.92, 48.28)	23113.79 (21006.39, 25198.22)	55.16 (50.13, 60.39)	19.51 (7.51, 32.2)
Caribbean	2400.61 (2034.38, 2946.3)	7.57 (6.72, 8.87)	8451.39 (6865.67, 10354.28)	16.93 (13.7, 20.92)	123.77 (79.18, 179.23)
**YLD**					
Global	33800.6 (23550.19, 45745.61)	0.70 (0.49, 0.940)	57068.01 (39981.62, 76338.40)	0.72 (0.50, 0.96)	1.86 (0.57, 3.35)
East Asia	6461.53 (4357.38, 8992.43)	0.52 (0.36, 0.73)	9213.77 (6322.25, 12911.4)	0.60 (0.40, 0.83)	14.02 (11.92, 16.55)
Southeast Asia	1482.08 (1008.87, 2089.48)	0.33 (0.23, 0.46)	2454.48 (1672.07, 3425.34)	0.36 (0.25, 0.50)	8.81 (7.37, 10.41)
Central Asia	374.75 (256.68, 515.69)	0.56 (0.38, 0.77)	527.5 (359.41, 735)	0.57 (0.39, 0.79)	1.81 (−0.08, 3.63)
High-income Asia Pacific	1739.51 (1198.60, 2333.35)	0.93 (0.65, 1.25)	2909.13 (1991.34, 3943.44)	1.05 (0.73, 1.41)	13.5 (10.20, 17.06)
South Asia	3587.87 (2412.77, 5024.02)	0.34 (0.23, 0.48)	6887.60 (4692.36, 9654.53)	0.38 (0.26, 0.53)	10.32 (8.69, 12.11)
Central Europe	1005.45 (696.71, 1373.12)	0.80 (0.56, 1.10)	1086.98 (746.37, 1460.78)	0.89 (0.61, 1.21)	10.12 (7.77, 12.54)
Eastern Europe	1570.87 (1077.89, 2181.94)	0.70 (0.48, 0.97)	1535.46 (1049.49, 2121.1)	0.73 (0.50, 1.01)	4.32 (2.55, 6.30)
Western Europe	6916.85 (4790.61, 9100.59)	1.41 (0.98, 1.86)	12063.70 (8288.2, 15935.77)	1.77 (1.22, 2.33)	25.18 (22.01, 28.29)
Southern Latin America	404.41 (278.3, 543.93)	0.83 (0.57, 1.12)	732.52 (509.39, 974.33)	1.01 (0.71, 1.36)	22.34 (17.63, 26.30)
High-income North America	5158.58 (3599.96, 6824.02)	1.64 (1.14, 2.16)	9333.71 (6642.86, 12243.18)	1.88 (1.34, 2.47)	15.11 (6.92, 25.50)
Andean Latin America	131.41 (89.10, 183.88)	0.37 (0.26, 0.52)	254.08 (174.61, 349.50)	0.40 (0.28, 0.56)	8.14 (4.99, 10.95)
Central Latin America	761.13 (516.31, 1053.70)	0.48 (0.33, 0.67)	1329.94 (915.54, 1828.29)	0.53 (0.36, 0.73)	9.42 (7.15, 12.11)
Tropical Latin America	760.34 (517.12, 1061.99)	0.52 (0.35, 0.71)	1397.76 (969.35, 1907.94)	0.60 (0.42, 0.82)	16.95 (13.06, 21.43)
North Africa and Middle East	1626.98 (1102.95, 2254.79)	0.53 (0.36, 0.73)	3308.29 (2269.07, 4603.9)	0.55 (0.38, 0.76)	3.55 (2.01, 5.10)
Central Sub-Saharan Africa	136.03 (92.65, 191.01)	0.30 (0.21, 0.42)	334.54 (227.59, 470.20)	0.30 (0.21, 0.42)	0.76 (−1.69, 3.26)
Eastern Sub-Saharan Africa	479.87 (324.06, 671.58)	0.31 (0.21, 0.43)	1113.98 (752.18, 1554.22)	0.32 (0.22, 0.44)	3.86 (2.86, 4.92)
Southern Sub-Saharan Africa	191.73 (130.30, 266.67)	0.38 (0.27, 0.53)	309.29 (212.52, 431.30)	0.40 (0.28, 0.56)	4.45 (3.13, 5.94)
Western Sub-Saharan Africa	506.99 (342.18, 708.31)	0.31 (0.21, 0.43)	1261.42 (853.55, 1773.88)	0.32 (0.22, 0.44)	3.42 (2.58, 4.23)
Oceania	18.28 (12.34, 25.69)	0.3 (0.21, 0.42)	37.27 (25.46, 52.52)	0.30 (0.21, 0.41)	−1.96 (−4.69, 0.84)
Australasia	303.33 (208.63, 404.58)	1.35 (0.93, 1.80)	709.57 (490.27, 941.35)	1.71 (1.19, 2.26)	26.30 (19.37, 33)
Caribbean	182.61 (126.57, 248.5)	0.54 (0.37, 0.73)	267.03 (186.61, 361.73)	0.55 (0.38, 0.75)	2.31 (−0.17, 4.80)
**YLL**					
Global	590563.76 (562254.06, 628441.15)	12.55 (12.05, 13.19)	977538.58 (926348.26, 1025429.87)	11.94 (11.30, 12.53)	−4.86 (−10.71, 1.88)
East Asia	145824.46 (128648.03, 164532.34)	12.49 (11.07, 14.05)	103866.96 (89445.70, 119783.67)	6.23 (5.45, 7.09)	−50.11 (−58.65,−39.99)
Southeast Asia	9976.37 (8684.10, 11565.76)	2.76 (2.39, 3.13)	18236.42 (14925.97, 22196.65)	2.65 (2.18, 3.22)	−4.02 (−21.83, 14.57)
Central Asia	812.55 (700.25, 1013.13)	1.42 (1.24, 1.79)	1680.33 (1467.58, 1933.98)	1.84 (1.61, 2.11)	29.27 (0.41, 57.26)
High-income Asia Pacific	34391.98 (32598.18, 36483.71)	20.13 (18.48, 22.29)	50581.01 (45452.57, 54772.78)	13.61 (12.43, 14.57)	−32.38 (−41.04,−24.05)
South Asia	23425.72 (17514.04, 29950.16)	2.74 (1.97, 3.68)	65736.57 (51380.09, 81332.34)	4.12 (3.21, 5.11)	50.24 (18.95, 89.09)
Central Europe	15716.99 (15154.80, 16413.13)	13.27 (12.66, 14.06)	21037.36 (18144.93, 24002.57)	13.45 (11.49, 15.51)	1.32 (−12.94, 17.03)
Eastern Europe	10381.62 (9050.47, 13849.17)	4.15 (3.65, 5.46)	29035.33 (25781.93, 32248.89)	9.96 (8.91, 11.02)	139.98 (74.76, 191.90)
Western Europe	162445.24 (158821.11, 165324.18)	35.1 (34.29, 35.97)	271086.57 (251170.14, 289081.57)	37.62 (34.88, 40.06)	7.16 (−0.81, 14.73)
Southern Latin America	3551.94 (3360.44, 3753)	7.43 (7.04, 7.84)	16454.87 (14917.84, 17662.02)	21.29 (19.32, 22.94)	186.53 (159.09, 212.06)
High-income North America	110519.7 (107667.40, 113278.24)	35.69 (34.76, 36.64)	243240.06 (234099.78, 249719.10)	45.03 (43.51, 46.25)	26.18 (21.67, 30.60)
Andean Latin America	1493.30 (1311.10, 1694.55)	5.44 (4.79, 6.18)	4469.66 (3520.72, 5594.46)	7.62 (6.01, 9.52)	40.1 (8.41, 80.44)
Central Latin America	8540.92 (8126.66, 8984.78)	6.86 (6.63, 7.11)	30003.18 (24869.81, 35597.06)	12.21 (10.13, 14.51)	77.86 (45.85, 111.83)
Tropical Latin America	13610.42 (12785.64, 14665.37)	10.99 (10.46, 11.69)	43353.48 (39612.65, 46355.59)	17.98 (16.40, 19.30)	63.51 (41.51, 79.84)
North Africa and Middle East	32358.21 (20550.87, 55563.11)	8.87 (6.52, 13.39)	38320.02 (30629.59, 47074.26)	7.29 (5.82, 8.97)	−17.84 (−47.13, 19.10)
Central Sub-Saharan Africa	367.62 (264.20, 546.24)	0.75 (0.52, 1.20)	730.96 (549.08, 963.01)	0.68 (0.48, 0.95)	−8.74 (−37.30, 25)
Eastern Sub-Saharan Africa	1259.88 (984.53, 1664.58)	0.72 (0.50, 1.10)	2611.94 (2137.47, 3162.18)	0.70 (0.53, 0.90)	−2.23 (−24.82, 21.49)
Southern Sub-Saharan Africa	830.17 (717.97, 947.57)	2.26 (1.92, 2.59)	1378.11 (1113.30, 1740.13)	2 (1.61, 2.53)	−11.45 (−32.94, 22.62)
Western Sub-Saharan Africa	2904.78 (2192.13, 3727.32)	2.26 (1.63, 2.95)	4907.83 (3849.77, 6230.45)	1.64 (1.29, 2.10)	−27.67 (−50.27, 1.83)
Oceania	163.73 (111.25, 226.23)	4.19 (2.82, 5.88)	219.34 (152.77, 311.34)	2.3 (1.59, 3.32)	−45.06 (−57.93,−27.91)
Australasia	9770.16 (9330.12, 10207.42)	44.81 (42.66, 47.01)	22404.22 (20249.28, 24424.65)	53.46 (48.23, 58.57)	19.30 (6.98, 32.37)
Caribbean	2218 (1858.03, 2766.82)	7.03 (6.18, 8.30)	8184.36 (6603.55, 10106.40)	16.38 (13.20, 20.43)	133.07 (84.79, 192.48)
**Death**					
Global	17653.17 (17010.69, 18269.68)	0.43 (0.41, 0.44)	39081.23 (36566.69, 41129.62)	0.48 (0.45, 0.51)	12.39 (5.71, 19.27)
East Asia	2663.02 (2377.81, 2945.9)	0.25 (0.22, 0.27)	3072.55 (2627.3, 3569.17)	0.16 (0.14, 0.18)	−35.31 (−46.51,−22.89)
Southeast Asia	248.99 (214.58, 282.63)	0.08 (0.07, 0.09)	544.44 (443.3, 663.93)	0.08 (0.07, 0.10)	1.72 (−19.22, 23.69)
Central Asia	21.64 (18.66, 27.74)	0.04 (0.04, 0.05)	49.13 (43.22, 56.06)	0.06 (0.05, 0.06)	37.79 (4.77, 67.94)
High-income Asia Pacific	1141.94 (1098.81, 1179.04)	0.6 (0.57, 0.62)	2606.37 (2267.78, 2857.88)	0.59 (0.53, 0.64)	−0.99 (−10.5, 7.93)
South Asia	581 (415.94, 785.87)	0.08 (0.06, 0.12)	1945.55 (1498.29, 2432.51)	0.13 (0.10, 0.16)	54.31 (18.99, 101.97)
Central Europe	393.57 (382.73, 405.58)	0.29 (0.29, 0.30)	756.25 (653.93, 860.78)	0.40 (0.35, 0.46)	36.7 (18.54, 55.35)
Eastern Europe	297.33 (254.99, 408.05)	0.11 (0.10, 0.15)	971.69 (860.18, 1084.05)	0.30 (0.27, 0.34)	173.01 (92.82, 240.55)
Western Europe	6315.26 (6109.95, 6446.10)	1.17 (1.13, 1.19)	12606.56 (11547.44, 13506.02)	1.48 (1.37, 1.58)	26.95 (17.95, 35.13)
Southern Latin America	104.31 (98.98, 110.03)	0.22 (0.21, 0.23)	611.29 (554.79, 655.45)	0.75 (0.68, 0.81)	242.23 (210.3, 271.37)
High-income North America	4175.26 (4034.67, 4285.74)	1.22 (1.19, 1.26)	10697.33 (10121.78, 11091.36)	1.77 (1.68, 1.82)	44.19 (38.81, 49.30)
Andean Latin America	39.18 (34.63, 44.36)	0.17 (0.15, 0.19)	154.03 (121.92, 190.99)	0.27 (0.21, 0.33)	63.45 (27.76, 107.07)
Central Latin America	206.03 (199.25, 212.88)	0.20 (0.20, 0.21)	987.61 (823.04, 1166.85)	0.41 (0.34, 0.48)	102.23 (67, 139.48)
Tropical Latin America	339.4 (326.45, 356.83)	0.32 (0.31, 0.34)	1490.06 (1364.14, 1590.49)	0.61 (0.56, 0.66)	90.03 (68.72, 104.27)
North Africa and Middle East	560.77 (414.24, 841.72)	0.20 (0.16, 0.28)	1067.64 (855.02, 1318.24)	0.22 (0.18, 0.28)	9.26 (−26.75, 51.11)
Central Sub-Saharan Africa	7.23 (5.08, 11.68)	0.02 (0.01, 0.04)	14.83 (10.65, 20.57)	0.02 (0.01, 0.03)	−11.41 (−40.72, 28.01)
Eastern Sub-Saharan Africa	23.31 (16.85, 34.40)	0.02 (0.01, 0.03)	47.72 (36.74, 60.85)	0.02 (0.01, 0.02)	−8.80 (−30.36, 15.93)
Southern Sub-Saharan Africa	21.69 (18.36, 25.06)	0.07 (0.06, 0.08)	37.45 (30.16, 47.43)	0.06 (0.05, 0.07)	−13.55 (−35.57, 21.73)
Western Sub-Saharan Africa	75.18 (54.79, 97.51)	0.07 (0.05, 0.09)	120.67 (95.65, 154.05)	0.05 (0.04, 0.06)	−29.89 (−52.67, 0.04)
Oceania	4.87 (3.28, 6.84)	0.14 (0.10, 0.20)	6.21 (4.26, 9.02)	0.08 (0.05, 0.11)	−47.87 (−59.78,−32.31)
Australasia	379.17 (360.49, 395.74)	1.63 (1.55, 1.70)	1023.71 (914.65, 1126.65)	2.13 (1.91, 2.34)	30.60 (18.06, 42.89)
Caribbean	54.02 (49.50, 60.30)	0.19 (0.18, 0.21)	270.14 (223.52, 321.49)	0.53 (0.44, 0.63)	179.60 (129.52, 239.82)

*Data in parentheses are 95% uncertainty intervals (UI). MND, motor neuron disease; GBD, global burden of disease; DALY, disability-adjusted life-year; YLD, years lived with disability; YLL, years of life lost*.

Globally, 268,674 individuals (95% UI, 231893.92–310663.85) had MND in 2019. The number of patients with MND in 2019 was 1.7 times higher than in 1990 (159074.07 [95% UI, 134173.93–187017.72]). The age-standardized incidence of MND in 2019 was 0.79 (95% UI, 0.72–0.88), and the number of patients was 63,700 (95% UI, 57295.90–71343.33). The global age-standardized DALYs value of MND was 12.66 (95% UI, 11.98–13.29), and the count was 1034606.59 (95% UI, 979910.92–1085401.11). The YLD and YLL values of MND were 57,068.01 (95% UI, 39981.62–76338.40) and 977538.58 (95% UI, 926348.26–1025429.87), respectively. The global death count of MND in 2019 was 39081.23 (95% UI, 36566.69–41129.62).

High-income North America, Western Europe, Australasia, and Asia Pacific, as well as Southern Latin America had higher age-standardized prevalence rates. The age-standardized prevalence rates were low in the following regions: Oceania, Central sub-Saharan Africa, Western sub-Saharan Africa, Eastern sub-Saharan Africa, and Southeast Asia.

The age-standardized incidence rates were high in Australasia, high-income North America, Western Europe, Southern Latin America, and high-income Asia Pacific and low in Southeast Asia, South Asia, Oceania, Andean Latin America, and Central Asia.

**Figure 2:**
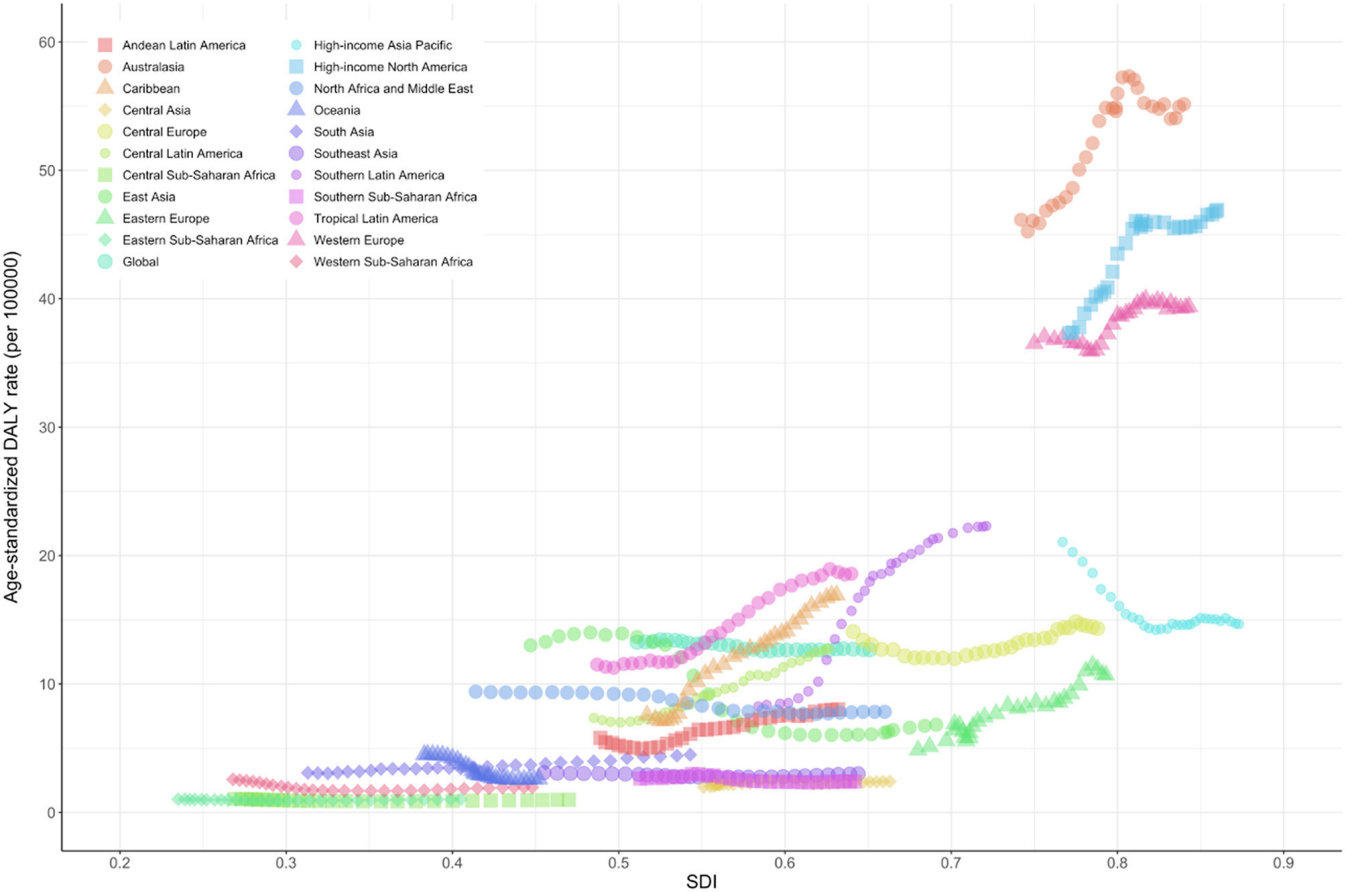
Global disability-adjusted life-years and its age-standardized rate of motor neuron diseases by age and sex. Values are dotted at the midpoints of 5-year age categories. Shaded areas represent 95% uncertainty intervals of the age-standardized DALY rates. DALY, disability-adjusted life-years; UI, uncertainty interval.

The age-standardized DALY rates were high in Australasia, high-income North America, Western Europe, Southern Latin America, and Tropical Latin America. Central sub-Saharan Africa, Eastern sub-Saharan Africa, Western sub-Saharan Africa, Southern sub-Saharan Africa, and Central Asia had lower age-standardized DALY rates. The global age-standardized DALY rates of motor neuron diseases by age and sex are shown in Figure 2.

The age-standardized rates of deaths caused by MND showed a similar pattern as DALYs. Australasia, high-income North America, Western Europe, Southern Latin America, and Tropical Latin America were the top five regions with high age-standardized death rates. Central sub-Saharan Africa, Eastern sub-Saharan Africa, Western sub-Saharan Africa, Southern sub-Saharan Africa, and Central Asia had relatively low age-standardized death rates.

### Regional Trend of Motor Neuron Disease Between 1990 and 2019

Changes in the age-standardized prevalence rates between 1990 and 2019 were most prominent in Australasia and Western Europe but lowest in Oceania and Central sub-Saharan Africa.

Changes in the age-standardized DALYs and death rates between 1990 and 2019 showed a similar pattern. The highest increase in the DALY and death were observed in Southern Latin America and the Caribbean. The lowest changes in the DALY and death were observed in Oceania and East Asia.

### 2019 GBD of Motor Neuron Diseases by Country

Prevalence, incidence, DALYs, YLDs, YLLs, and death due to MNDs by country in counts and age-standardized rates for both sexes for 2019 are listed in Supplementary Table 1. The age-standardized prevalence rates were high in Canada, Andorra, Finland, Ireland, and Sweden. In contrast, Kiribati, Somalia, Burundi, Central African Republic, and Solomon Islands had lower age-standardized prevalence of MND than other countries.

In 2019, the age-standardized incidence of MND was low in Malaysia, Seychelles, Indonesia, Maldives, and Philippines. In contrast, Ireland, Finland, Australia, United Kingdom, and Andorra had high age-standardized incidence of MND.

Age-standardized DALYs and death were high in the following countries: Ireland, Australia, Andorra, New Zealand, and Finland. The age-standardized DALYs and death were low in the following countries: Somalia, Central African Republic, Burundi, and Democratic Republic of the Congo South.

### National Trend of Motor Neuron Disease Between 1990 and 2019

Between 1990 and 2019, the DALYs rates were increased to the greatest extent in Barbados, Costa Rica, and Uruguay. The DALYs rates decreased in the following countries: Slovenia, Guam, Bosnia and Herzegovina, and Republic of Korea. Portugal, Italy, Lithuania, and Costa Rica showed the highest increase in age-standardized prevalence and incidence rates over the examined period. Sudan showed low DALY but an increased death rate in 2019 compared to 1990.

### Association Between Prevalence and DALYs of MNDs According to the SDI

**Figure 3:**
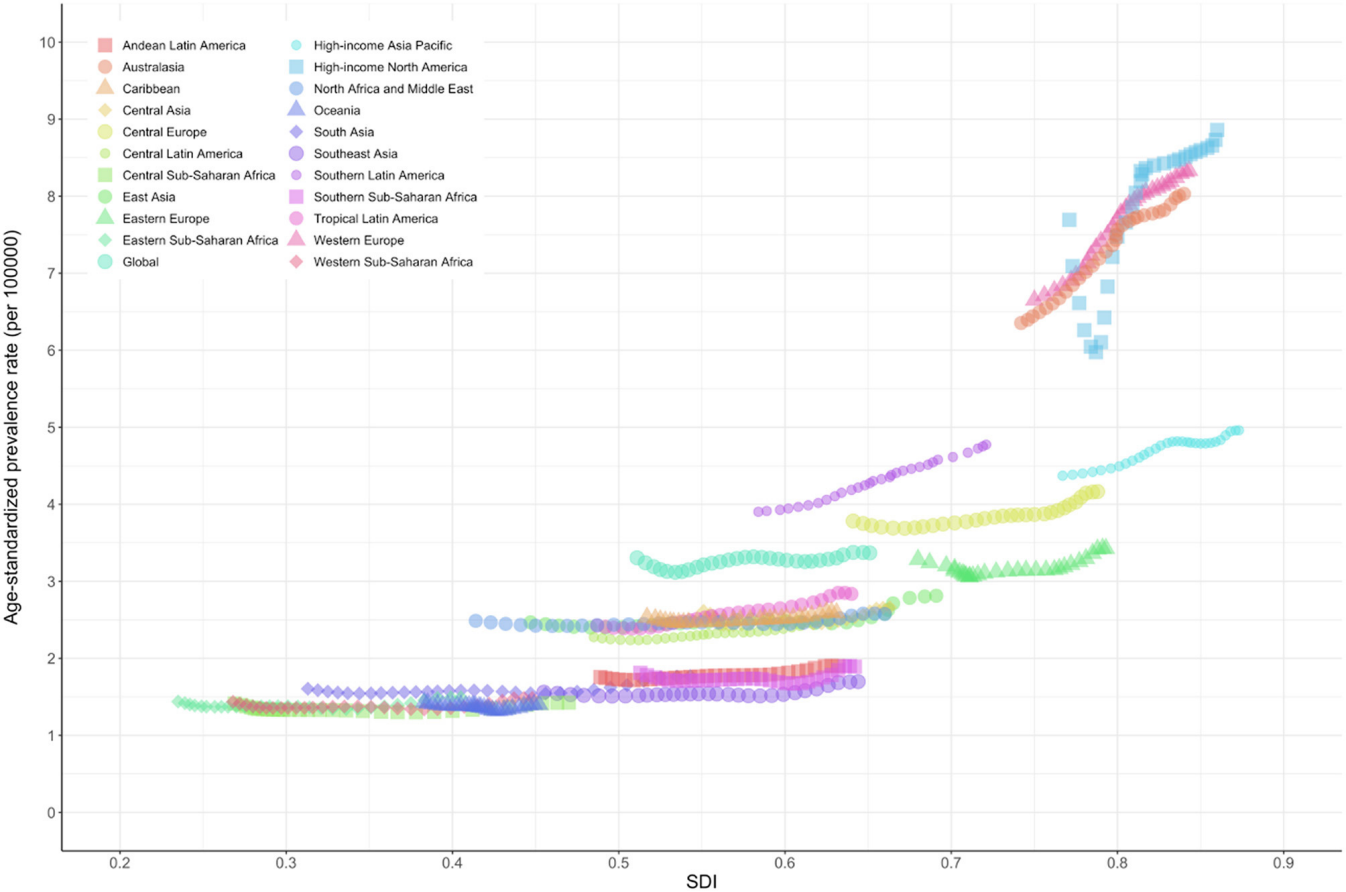
Age-standardized prevalence rates of motor neuron diseases by Global Burden of Disease regions by sociodemographic index, 1990–2019. Age-standardized prevalence rates are presented as number affected by motor neuron diseases per 100,000 population. SDI, sociodemographic index.

**Figure 4:**
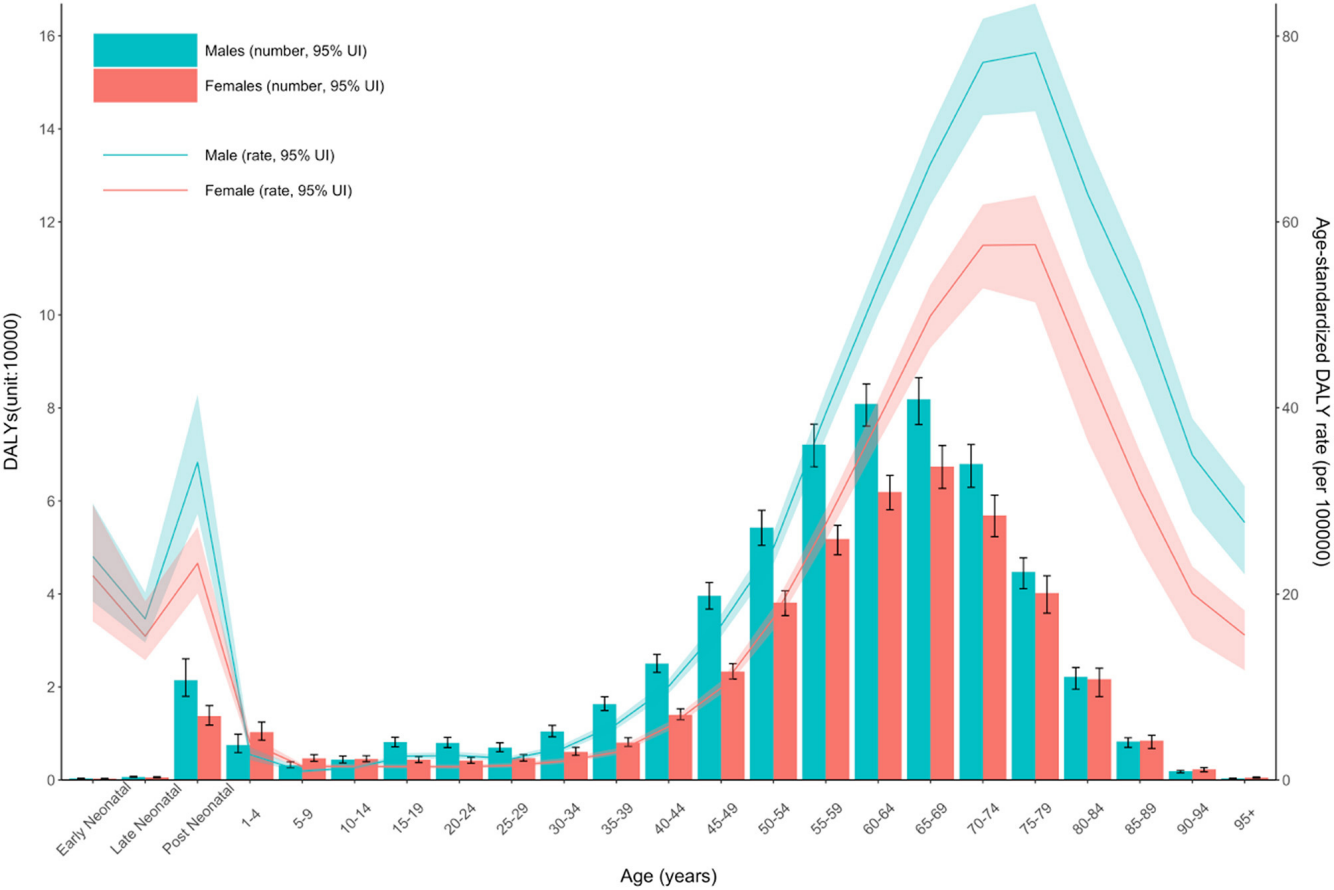
Age-standardized DALY rate of motor neuron diseases by Global Burden of Disease regions by sociodemographic index, 1990–2019. Age-standardized DALY rates are presented as rates per 100,000 population. DALY, disability-adjusted life-years; SDI, sociodemographic index.

**Figure 5:**
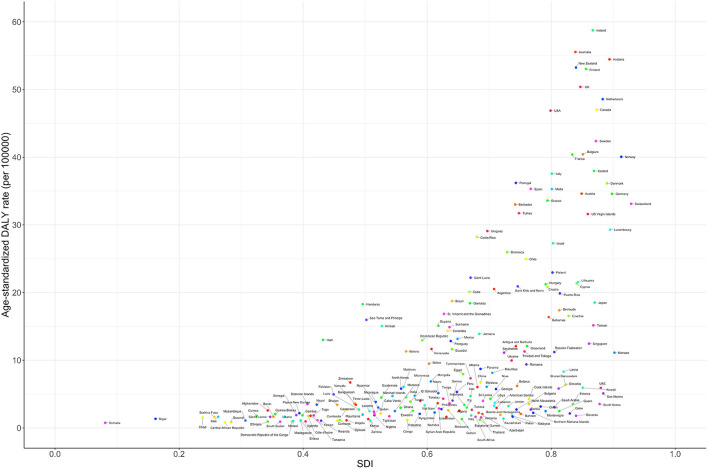
Age-standardized DALY rate of motor neuron diseases according to country-specific sociodemographic index. Age-standardized DALY rates are presented as rates per 100,000 population. DALY, disability-adjusted life-years; SDI, sociodemographic index.

The age-standardized prevalence rate (per 100,000) was relatively high in high-income North America, Western Europe, Australasia, and high-income Asia-Pacific regions with high SDI levels but was low in the sub-Saharan African region (Figure 3). The age-standardized DALY rate (per 100,000) for MNDs was also relatively high in Australasia, high-income North America, Western Europe, and high-income Asia-Pacific regions. In contrast, the age-standardized DALY rate of the sub-Saharan African region was the lowest among all countries (Figure 4). Figure 5 shows the age-standardized DALY rate for each country according to SDI. Ireland, Australia, New Zealand, Finland, United Kingdom, the Netherlands, United States, and Canada showed relatively high age-standardized DALY rates and SDIs (Figure 5).

## Discussion

We evaluated the burden of MNDs (estimated as incidence, prevalence, and DALYs) worldwide in 204 countries and territories from 1990 to 2019 using spatial Bayesian analyses. According to 2019 GBD estimates, age-standardized prevalence were 3.37 (95% UI, 2.9–3.87) per 100,000 population and age-standardized incidence were 0.79 (95% UI, 0.72–0.88) per 100,000 person-years for MND worldwide. In 2019, age-standardized DALY rate were 12.66 (95% UI, 11.98–13.29) per 100,000 population and age-standardized death rate were 0.48 (95% UI, 0.45–0.51) per 100,000 person-years associated with MND around the world. Global prevalence and deaths related to MNDs increased every year without significant changes in incidence. More than half of the prevalence and deaths due to MNDs occurred in three high-income regions (North America, Western Europe, and Australasia). In general, the prevalence, incidence, and DALYs value of MND were high in regions with high SDI, except in high-income East Asia where these values were relatively low despite similar SDI. These findings might suggest that not only sociodemographic development but also the genetic background might be responsible for the MND burden. Compared with the previous 2016 GBD MND results (9), our results showed that the global prevalence and DALYs of MNDs continued to increase similar to those in 2016, and the regional change of prevalence, DALYs showed a similar patterns as in 2016.

The age-standardized prevalence of MND seems to be increasing globally, a phenomenon that is more obvious in high-income countries. In contrast, the global age-standardized incidence of MND did not seem to increase to the same extent. However, when categorized by subcontinent in 2019, most of the age-standardized incidence increased significantly in the middle, high-middle, and high SDI regions. In the low and low-middle SDI regions, the age-standardized incidence either decreased or did not change significantly from 2009 to 2019. This phenomenon could be affected by whether accurate or early diagnosis is possible in the area where the incidence is analyzed. Since the El Escorial criteria were established in 1994 (14), the ALS diagnostic criteria were revised in 2000 (revised El Escorial criteria) and in 2008 (Awaji criteria) for early diagnosis and inclusion of more harmonized patients suitable for clinical trials (18, 19). The application of the latest diagnostic criteria for more accurate case ascertainment and access to specialists or medical institutions are largely affected by regional income levels. Similar geographical differences in MND incidence according to socioeconomic status or access to healthcare systems were also reported in the United States and Europe (20, 21). The non-significant changes in age-standardized incidence of MNDs in the low and low-middle SDI regions may indicate that the incidence is actually small in these region, but may be an underestimated number. The remarkable growth in prevalence with relatively stable or mild increment in incidence in the above regions could have been influenced by the increase in survival due to the development of therapies, such as the universal use of noninvasive ventilators in ALS or application of novel drugs (e.g., nusinersen for spinal muscular atrophy) in clinical practice (22, 23).

Racial diversity and geographic gradients regarding MND incidence were reported in several epidemiology studies. One study performed in New Jersey showed that the risk of ALS was higher in White patients than in Black and Asian patients (23). Mortality due to ALS, which is a surrogate marker of incidence, was the lowest in people of mixed ancestry compared to Black and White patients in Cuba (24). Because Cuba offers free national healthcare to all citizens, socioeconomic status was not the main factor for this discrepancy. In the meta-analysis pooled from 45 geographic areas, ALS incidence rates of populations with European ancestry (North America, Europe, New Zealand) showed homogeneous rates [1.81 (1.66–1.97)/100,000 person-years], which are higher than those of the populations of East Asia and South Asia [0.83 (0.42–1.24)/100,000 person-years; 0.73 (0.58–0.89)/100,000 person-years, respectively] (8). As observed in the previous 2016 GBD Study, our results showed that these geographical heterogeneities were independent of SDI in high-income East Asia, which supports the risk associated with genetic background and ancestry (18). *C9ORF72*, the most common causative gene for ALS (40% of familial ALS and 8% of sporadic), may be one possible reason for the high ALS incidence in North American and European populations (25). The frequency of *C9ORF72* mutation was much lower in South and East Asia (5.9% in familial and 1.6% in sporadic ALS in Iran; <4% in Japan and Korea) (26–28). However, as we observed in Guam and Kii Peninsula cases, where the extremely high ALS incidence rates dropped rapidly with westernization, both genetic and environmental factors might influence ALS incidence (29).

We analyzed the GBD project-specific measurement “DALY” to summarize the overall burden of a disease. The patterns of DALY and MND-associated death showed somewhat similar trend to that of prevalence in each continents: high age-standardized DALY number and rate in high SDI regions (except in the high-income Asia-Pacific region) and relatively low age-standardized DALY number and rate in middle and low SDI regions. The exception of reduced DALY in the high-income Asia-Pacific region might be partially due to different frequencies of ALS subtypes, different ratio of familial ALS and the coincidence of non-motor phenotypes (e.g., frontotemporal dementia) compared to other subcontinents. Bulbar-onset ALS, which is well-known for its poor prognosis compared to limb-onset ALS, is more common in regions of European ancestry than in Asia (30). Similarly, frontotemporal dementia, highly connected with the presence of *C9ORF72* mutations, is more common in regions of European ancestry and might be the cause of high disability and death (25–28).

The number of DALY and age-standardized rates were consistently higher in males than in females in all age groups between 1990 and 2019. Because the effects of sex on survival were not dominant, this male preponderance of DALY might explain by the difference in prevalence between the sexes. The male preponderance in MND, especially in limb-onset ALS, was consistent with previous reports (8, 9, 31). Possible causes of the difference between males and females include the differences in exposure to environmental risk factors, response to exogenous toxins, and the nervous system structure and damage correction ability (31).

The age-standardized rate of DALYs of MND dramatically increased after age of 50, with a peak at 70–79 years followed by a rapid decline in both males and females. Given that ALS, which accounts for the largest proportion of MND, has a very short mean or median survival of 24–50 months from symptom onset, the changes in DALYs according to age in our results are consistent with the previous results showing the highest incidence between 70 and 74 years of age (20, 32). The rapid decline in DALYs and prevalence after the age of 80 requires caution in interpreting the phenomenon in that diagnostic ascertainment is not easy in the elderly. In elderly patients, it is generally more difficult to differentiate ALS mimic syndromes, and other comorbidities that can cause death are common. Moreover, elderly patients are less frequently referred to tertiary centers (because their weakness is more easily considered as due to aging and not pathological). The higher frequency of the bulbar-onset ALS with poor prognosis in older patients than in young patients may also be other causes of the rapid decline in DALYs and prevalence in people over 80 years of age. Another small peak of DALY occurs in the postneonatal period and this rate/number decreases until age 4. The high DALY rate in early childhood is considered to be a phenomenon from MNDs other than ALS—occurring mainly in childhood such as spinal muscular atrophy and hereditary spastic paraplegia—are included in the analysis.

This study has the same general limitations that inevitably occur in the design of GBD studies (33). First, although global epidemiology data were analyzed, relatively less data from regions other than Europe or North America were included in this study. The relatively small number of epidemiology studies in South and Central Asia, sub-Saharan Africa, and Latin America, and the lack of access to medical facilities for diagnosing MNDs in these regions might be factors contributing to the relative low prevalence or incidence of MNDs in these regions. Second, the ICD version used for evaluating the death rate was changed from ICD-9 to ICD-10 during the study period. This evolution in the classification may have influenced the results. Third, the diagnosis of MND is known to be clinically challenging, and there is a possibility that certain categories, especially older individuals or ethnic minorities, may be underdiagnosed. In addition, the diagnostic criteria for MNDs have changed between 1990 and 2019, leading to differences in diagnostic sensitivity. However, the systematic bias of GBD estimates due to changes in diagnostic criteria during this study period was unclear (9). Fourth, because prevalence and DALYs are values related to incidence, disease duration, and survival, they are affected by the treatment methods or abilities of each region. Prevalence and DALYs may be high in high-income regions as access to and quality of treatment provision is high, and survival and disease duration are increased. In addition, treatment is affected not only by income level but also by the experience and preference of the local medical staff or the social climate for allowing treatment. This difference in treatment affects disease duration and survival. For example, in Japan, the rate of tracheostomy is 30%, whereas it is only 0–10% in Europe and the United States (34, 35). In contrast, non-invasive ventilators are used in 15–35% of patients in the United States, which is much higher than in Japan or Europe (36). Fifth, the prevalence rate confirmed in this study is slightly lower than the rates for ALS or early-childhood-onset MND (spinal muscular atrophy, hereditary spastic paraplegia) analyzed in other regional or meta-analysis studies. This is because this study analyzed diverse MNDs as one disease group and included the estimates of various races and regions.

In conclusion, the GBD of MND provides information on worldwide epidemiology, social influence, and risk factors of MNDs by using a standardized protocol. The global burden of MNDs is continuously increasing, especially in middle- and high-income areas. Because the number of epidemiology studies conducted in South and Central Asia, sub-Saharan Africa, and Latin America is small, and there is a high possibility that MNDs are underdiagnosed in the local system, the actual burden is expected to be higher than the presented results. In addition, the aging of the global population is expected to increase the share of the social burden of, for example, ALS, a neurodegenerative disease that mainly occurs in old age. The results of our analysis of the 2019 GBD 2019 Study may offer objective, recent information for resource allocation and healthcare planning related to MNDs at global and national levels.

## Data Availability Statement

Publicly available datasets were analyzed in this study. This data can be found at: the Institute for Health Metrics and Evaluation (IHME) Global Health Data Exchange (GHDx), http://ghdx.healthdata.org/gbd-results-tool.

## Ethics Statement

Ethical review and approval was not required for the study on human participants in accordance with the local legislation and institutional requirements. Written informed consent from the patients/participants or patients/participants' legal guardian/next of kin was not required to participate in this study in accordance with the national legislation and the institutional requirements.

## Author Contributions

T-JS: concept and design, statistical analysis, administrative, technical, or material support, final approval of the version to be published, and had full access to all the data in the study and takes responsibility for the integrity of the data and the accuracy of the data analysis. JP, J-EK, and T-JS: analysis and/or interpretation of data, drafting of the manuscript, critical writing or revising the intellectual content. All authors contributed to the article and approved the submitted version.

## Funding

This work was supported by the Basic Science Research Program through the National Research Foundation of Korea (NRF) funded by the Ministry of Education (NRF-2021R1F1A1048113 to T-JS). The funders had no role in study design, data collection and analysis, the decision to publish, or preparation of the manuscript.

## Conflict of Interest

The authors declare that the research was conducted in the absence of any commercial or financial relationships that could be construed as a potential conflict of interest.

## Publisher's Note

All claims expressed in this article are solely those of the authors and do not necessarily represent those of their affiliated organizations, or those of the publisher, the editors and the reviewers. Any product that may be evaluated in this article, or claim that may be made by its manufacturer, is not guaranteed or endorsed by the publisher.
