# Supplementary material for: The Global Burden of Motor Neuron Disease: An Analysis of the 2019 Global Burden of Disease Study
Source: Front Neurol. 2022 Apr 21;13:864339. doi: 10.3389/fneur.2022.864339 (PMC9068990; doi:10.3389/fneur.2022.864339)
Supplement: Supplementary file 1 [file Table_1.docx]

Supplementary Material

# Supplementary Table. Prevalence, Incidence, DALYs, YLL, YLD and Death of MNZ in counts and age-standardized rate for both sexes combined in 1990 and 2019, with percentage change between 1990 and 2019 by the nation

|  | **1990** | | **2019** | | **Percentage change in  age-standardized rates  between 1990 and 2019 (%)** |
| --- | --- | --- | --- | --- | --- |
|  | **Counts (95% UI)** | **Age-standardized Rate (per 100k)** | **Counts (95% UI)** | **Age-standardized Rate (per 100k)** |  |
| **Prevalence** | | | | | |
| **Afghanistan** | 192.62 (156.46, 238.47) | 1.93 (1.56, 2.38) | 651.81 (521.21, 814.52) | 1.93 (1.58, 2.37) | 0.16 (-4.02, 4.17) |
| **Albania** | 95.99 (77.56, 116.61) | 2.96 (2.42, 3.58) | 90.05 (74.56, 107.6) | 3.18 (2.61, 3.82) | 7.22 (3.15, 11.9) |
| **Algeria** | 525.27 (418.11, 648.41) | 2.31 (1.88, 2.82) | 997.15 (798.54, 1217.09) | 2.36 (1.91, 2.85) | 2 (-1.59, 5.67) |
| **American Samoa** | 0.91 (0.72, 1.13) | 2.05 (1.69, 2.45) | 1.07 (0.86, 1.31) | 1.95 (1.58, 2.36) | -4.93 (-9.53, -0.35) |
| **Andorra** | 5.81 (4.95, 6.72) | 10.15 (8.68, 11.66) | 14.42 (12.28, 16.7) | 11.71 (10.07, 13.44) | 15.43 (10.04, 21.05) |
| **Angola** | 137.38 (108.95, 169.55) | 1.64 (1.33, 1.99) | 420.63 (332.5, 525.75) | 1.71 (1.39, 2.09) | 4.51 (0.57, 8.45) |
| **Antigua and Barbuda** | 1.55 (1.27, 1.88) | 2.58 (2.13, 3.1) | 2.53 (2.09, 3.01) | 2.75 (2.28, 3.28) | 6.68 (1.74, 11.11) |
| **Argentina** | 1201.19 (1011.61, 1408.92) | 3.66 (3.08, 4.29) | 2133.38 (1815.24, 2477.2) | 4.45 (3.78, 5.18) | 21.82 (16.65, 27.33) |
| **Armenia** | 93.57 (75.28, 113.95) | 2.76 (2.25, 3.33) | 88.52 (72.49, 106.94) | 2.86 (2.32, 3.46) | 3.37 (-0.68, 8.03) |
| **Australia** | 1167.04 (1019.55, 1346.4) | 6.24 (5.45, 7.18) | 2806.83 (2422.99, 3239.36) | 7.98 (6.94, 9.09) | 27.9 (20.25, 35.55) |
| **Austria** | 552.03 (478.53, 634.64) | 5.85 (5.08, 6.72) | 1008.67 (860.84, 1163.78) | 7.75 (6.72, 8.86) | 32.41 (24.3, 41.63) |
| **Azerbaijan** | 184.27 (147.51, 226.76) | 2.57 (2.09, 3.14) | 279.78 (226.13, 339.98) | 2.66 (2.16, 3.19) | 3.38 (-0.97, 7.86) |
| **Bahamas** | 7.56 (6.22, 9.11) | 3.05 (2.58, 3.61) | 11.72 (9.85, 13.87) | 3.05 (2.55, 3.61) | 0.04 (-4.43, 4.81) |
| **Bahrain** | 13.69 (10.92, 17.16) | 2.68 (2.18, 3.27) | 45.89 (36.28, 58.11) | 2.78 (2.25, 3.41) | 3.69 (-0.46, 7.9) |
| **Bangladesh** | 1632.07 (1287.95, 2066.21) | 1.64 (1.32, 2.02) | 2858.41 (2290.99, 3534.34) | 1.78 (1.44, 2.18) | 8.38 (4.25, 12.92) |
| **Barbados** | 8.44 (7.16, 9.87) | 3.22 (2.73, 3.77) | 12.07 (10.33, 13.97) | 3.43 (2.93, 3.97) | 6.53 (1.96, 11.24) |
| **Belarus** | 347.65 (285.57, 418.85) | 3.33 (2.7, 4) | 325.16 (271.45, 388.34) | 3.46 (2.83, 4.16) | 3.92 (-0.53, 8.31) |
| **Belgium** | 851.52 (732.29, 986.82) | 6.73 (5.83, 7.73) | 1488.97 (1275.02, 1727.35) | 8.66 (7.51, 9.97) | 28.74 (20.98, 37.94) |
| **Belize** | 3.85 (3.07, 4.68) | 2.2 (1.82, 2.63) | 9.6 (7.82, 11.56) | 2.38 (1.97, 2.85) | 8.25 (3.71, 13.5) |
| **Benin** | 57.04 (45.04, 70.85) | 1.42 (1.15, 1.73) | 157.08 (123.26, 197.75) | 1.46 (1.18, 1.77) | 3.05 (-0.63, 7.13) |
| **Bermuda** | 3.03 (2.59, 3.52) | 4.94 (4.23, 5.7) | 3.73 (3.22, 4.3) | 4.67 (4, 5.37) | -5.49 (-9.65, -1.22) |
| **Bhutan** | 10.31 (8.15, 12.98) | 1.81 (1.47, 2.22) | 15.36 (12.38, 18.94) | 2 (1.63, 2.45) | 10.53 (5.85, 15.88) |
| **Bolivia (Plurinational State of)** | 105.51 (85.32, 129.88) | 1.83 (1.52, 2.2) | 231.69 (190.88, 280.17) | 2.04 (1.7, 2.44) | 11.39 (6.82, 15.87) |
| **Bosnia and Herzegovina** | 159.49 (131.27, 191.33) | 3.46 (2.86, 4.13) | 125.19 (103.4, 149.81) | 3.71 (3.04, 4.46) | 7.32 (2.65, 12.44) |
| **Botswana** | 20.68 (16.36, 25.62) | 1.72 (1.41, 2.07) | 40.94 (32.88, 50.41) | 1.83 (1.5, 2.2) | 6.12 (1.83, 10.82) |
| **Brazil** | 3480.04 (2781.75, 4259.75) | 2.42 (1.98, 2.91) | 6384.91 (5389.57, 7596.14) | 2.84 (2.39, 3.37) | 17.13 (13.21, 21.71) |
| **Brunei Darussalam** | 4.63 (3.74, 5.8) | 2.24 (1.83, 2.72) | 9.88 (8.03, 12.01) | 2.39 (1.95, 2.87) | 6.81 (2.79, 10.84) |
| **Bulgaria** | 279.08 (231.51, 334.58) | 3.22 (2.65, 3.86) | 239.03 (199.25, 286.41) | 3.41 (2.8, 4.07) | 5.71 (0.78, 11.24) |
| **Burkina Faso** | 109 (86.11, 135.39) | 1.37 (1.12, 1.67) | 275.5 (217.13, 342.79) | 1.43 (1.16, 1.75) | 5.06 (1.1, 9.09) |
| **Burundi** | 58.61 (46.3, 73.52) | 1.28 (1.03, 1.56) | 126.43 (98.71, 158.37) | 1.27 (1.03, 1.55) | -0.16 (-3.6, 3.82) |
| **Cabo Verde** | 5.93 (4.77, 7.31) | 1.91 (1.55, 2.31) | 11.08 (9, 13.56) | 2.01 (1.65, 2.44) | 5.18 (1.44, 9.11) |
| **Cambodia** | 128.67 (100.57, 162.41) | 1.38 (1.12, 1.67) | 243.79 (195.93, 300.66) | 1.51 (1.23, 1.83) | 9.24 (4.91, 13.75) |
| **Cameroon** | 119.05 (94.54, 149.93) | 1.34 (1.08, 1.64) | 347.95 (274.04, 437.02) | 1.35 (1.09, 1.65) | 0.38 (-2.99, 4.18) |
| **Canada** | 3047.99 (2659.44, 3435.08) | 10.28 (9.03, 11.55) | 6200.54 (5349.61, 7148.85) | 11.98 (10.54, 13.61) | 16.5 (10.02, 23.58) |
| **Central African Republic** | 29.49 (23.15, 36.74) | 1.28 (1.05, 1.57) | 56.99 (44.96, 71.13) | 1.27 (1.03, 1.54) | -1.49 (-5.25, 2.58) |
| **Chad** | 74.01 (58.68, 91.32) | 1.48 (1.21, 1.81) | 204.64 (161.57, 255.28) | 1.54 (1.26, 1.89) | 4.46 (0.9, 8.5) |
| **Chile** | 556.18 (469.13, 660.13) | 4.42 (3.77, 5.18) | 1098.3 (943.99, 1267.48) | 5.43 (4.64, 6.29) | 22.79 (15.76, 29.11) |
| **China** | 29265.08 (23322.16, 36174.77) | 2.45 (1.99, 3.01) | 41753.76 (33980.99, 51200.48) | 2.81 (2.27, 3.41) | 14.34 (12.17, 17.02) |
| **Colombia** | 623.86 (497.82, 771.29) | 1.99 (1.63, 2.39) | 1121.16 (930.87, 1336.82) | 2.29 (1.9, 2.74) | 15.08 (9.23, 21.48) |
| **Comoros** | 6.63 (5.29, 8.24) | 1.71 (1.37, 2.08) | 11.45 (9.23, 14.09) | 1.71 (1.39, 2.08) | 0.22 (-3.25, 3.68) |
| **Congo** | 28.47 (22.59, 35.58) | 1.37 (1.12, 1.67) | 65.7 (52.17, 82.56) | 1.4 (1.13, 1.71) | 1.52 (-2.22, 5.55) |
| **Cook Islands** | 0.4 (0.32, 0.49) | 2.16 (1.78, 2.59) | 0.41 (0.34, 0.49) | 2.23 (1.84, 2.69) | 3.27 (-0.62, 8.42) |
| **Costa Rica** | 73.92 (61.15, 88.77) | 2.73 (2.3, 3.2) | 171.32 (146.76, 197.26) | 3.46 (2.96, 4) | 26.93 (20.89, 33.93) |
| **Croatia** | 218.02 (184.41, 255.8) | 4.39 (3.7, 5.18) | 211.99 (180.47, 247.65) | 4.65 (3.9, 5.51) | 5.72 (-1.37, 13.02) |
| **Cuba** | 330.46 (277.83, 392.88) | 3.03 (2.57, 3.57) | 406.85 (346.01, 472.93) | 3.22 (2.74, 3.78) | 6.35 (1.94, 11.66) |
| **Cyprus** | 37.88 (32.1, 43.99) | 4.64 (3.93, 5.38) | 91.67 (77.18, 107.98) | 5.53 (4.67, 6.42) | 19.2 (13.9, 24.8) |
| **Czechia** | 473.59 (396.28, 557.98) | 4.55 (3.8, 5.4) | 543.14 (462.55, 634.5) | 4.79 (4.03, 5.69) | 5.35 (-1.45, 12.97) |
| **Côte d'Ivoire** | 142.8 (112.2, 181.05) | 1.38 (1.11, 1.68) | 322.22 (252.06, 406.14) | 1.39 (1.12, 1.69) | 0.75 (-3.03, 4.23) |
| **Democratic People's Republic of Korea** | 542.77 (444.5, 651.13) | 2.69 (2.24, 3.21) | 751.25 (628.33, 896.06) | 2.75 (2.3, 3.28) | 2.16 (-2.3, 7.05) |
| **Democratic Republic of the Congo** | 426.92 (337.87, 540.18) | 1.36 (1.1, 1.68) | 987.15 (775.59, 1242.93) | 1.34 (1.08, 1.64) | -1.84 (-5.25, 1.82) |
| **Denmark** | 546.41 (470.14, 633.57) | 8.12 (7.07, 9.35) | 817.35 (701.9, 956.48) | 9.22 (8.01, 10.66) | 13.53 (7.21, 20.06) |
| **Djibouti** | 6.85 (5.38, 8.7) | 1.73 (1.4, 2.11) | 18.88 (15.07, 23.61) | 1.74 (1.41, 2.12) | 0.12 (-3.14, 3.53) |
| **Dominica** | 1.8 (1.5, 2.15) | 2.45 (2.07, 2.9) | 2.18 (1.86, 2.53) | 2.97 (2.51, 3.47) | 21.24 (14.46, 28.95) |
| **Dominican Republic** | 148.51 (118.65, 185.84) | 2.2 (1.8, 2.68) | 256.13 (208.55, 308.94) | 2.37 (1.95, 2.85) | 7.73 (2.84, 12.51) |
| **Ecuador** | 148.94 (119.63, 183.44) | 1.6 (1.3, 1.93) | 315.67 (259.93, 382.7) | 1.83 (1.52, 2.2) | 14.61 (8.93, 20.34) |
| **Egypt** | 1087.04 (861.15, 1349.84) | 2.12 (1.71, 2.61) | 2135.07 (1704.63, 2641.05) | 2.21 (1.78, 2.71) | 4.21 (0.57, 8.21) |
| **El Salvador** | 98.55 (78.13, 121.13) | 1.95 (1.59, 2.36) | 129.4 (104.59, 155.7) | 2.06 (1.67, 2.48) | 5.44 (1.32, 9.93) |
| **Equatorial Guinea** | 4.68 (3.72, 5.79) | 1.32 (1.07, 1.62) | 19.13 (15.05, 24.17) | 1.59 (1.29, 1.93) | 20.19 (14.96, 25.51) |
| **Eritrea** | 39.05 (30.84, 48.79) | 1.57 (1.29, 1.92) | 96.04 (76.39, 119.77) | 1.64 (1.34, 2) | 4.27 (0.33, 8.55) |
| **Estonia** | 60.17 (50.39, 71.45) | 3.83 (3.17, 4.57) | 52.77 (44, 62.31) | 3.98 (3.26, 4.75) | 3.88 (-1.43, 9.37) |
| **Eswatini** | 12.56 (9.85, 15.74) | 1.74 (1.43, 2.11) | 19.45 (15.61, 24.15) | 1.81 (1.48, 2.19) | 4.27 (0.26, 8.44) |
| **Ethiopia** | 596.85 (461.19, 758.13) | 1.39 (1.11, 1.74) | 1391.89 (1065.31, 1782.88) | 1.5 (1.19, 1.86) | 7.47 (5.08, 10.15) |
| **Fiji** | 12.97 (10.37, 16.23) | 1.76 (1.44, 2.12) | 16.57 (13.45, 20.1) | 1.84 (1.49, 2.21) | 4.3 (0.46, 8.07) |
| **Finland** | 633.34 (546.33, 733.74) | 10.05 (8.7, 11.56) | 1069.7 (912.58, 1255.16) | 11.66 (10.01, 13.45) | 15.94 (9.17, 23.69) |
| **France** | 5521.23 (4801.43, 6228.33) | 7.69 (6.72, 8.71) | 9248.22 (8001.38, 10585.27) | 9.07 (7.94, 10.3) | 18 (10.89, 25.89) |
| **Gabon** | 13.35 (10.75, 16.69) | 1.55 (1.26, 1.89) | 25.1 (20.05, 31.23) | 1.55 (1.25, 1.9) | 0.1 (-3.93, 3.89) |
| **Gambia** | 13.26 (10.55, 16.67) | 1.57 (1.27, 1.93) | 31.62 (24.99, 39.79) | 1.57 (1.27, 1.91) | -0.12 (-3.82, 4.03) |
| **Georgia** | 154.66 (127.95, 185.75) | 2.81 (2.31, 3.37) | 105.22 (87.11, 126.13) | 2.84 (2.32, 3.44) | 1.15 (-3.34, 5.58) |
| **Germany** | 6185.9 (5354.22, 7077.26) | 6.07 (5.26, 6.93) | 10681.08 (8992.54, 12374.68) | 7.79 (6.69, 8.94) | 28.41 (20.87, 36.54) |
| **Ghana** | 181.63 (142.63, 229.89) | 1.39 (1.12, 1.7) | 426.07 (337.61, 536.09) | 1.46 (1.19, 1.78) | 5.25 (1.08, 9.55) |
| **Greece** | 495.63 (415.2, 578.28) | 4.06 (3.41, 4.73) | 789.55 (671.12, 920.63) | 5.3 (4.54, 6.14) | 30.64 (21.37, 39.78) |
| **Greenland** | 4.13 (3.52, 4.78) | 8.5 (7.32, 9.76) | 5.79 (4.87, 6.81) | 9.48 (8.09, 10.93) | 11.63 (6.34, 16.69) |
| **Grenada** | 1.84 (1.49, 2.24) | 2.21 (1.83, 2.65) | 2.72 (2.28, 3.21) | 2.54 (2.12, 3.02) | 15.03 (9.42, 21.17) |
| **Guam** | 3.34 (2.77, 4.01) | 2.8 (2.36, 3.28) | 3.75 (3.08, 4.54) | 2.17 (1.78, 2.62) | -22.67 (-28.05, -16.77) |
| **Guatemala** | 136.87 (108.02, 168.31) | 1.85 (1.52, 2.26) | 334.3 (269.27, 413.42) | 1.92 (1.57, 2.33) | 3.46 (-0.45, 7.59) |
| **Guinea** | 73.43 (58.6, 90.84) | 1.39 (1.13, 1.69) | 152 (120.29, 190.83) | 1.41 (1.14, 1.71) | 0.92 (-2.83, 4.7) |
| **Guinea-Bissau** | 11.71 (9.13, 14.75) | 1.36 (1.1, 1.67) | 23.09 (18.17, 29) | 1.38 (1.12, 1.68) | 1.49 (-2.51, 5.42) |
| **Guyana** | 12.09 (9.5, 14.98) | 1.61 (1.32, 1.94) | 13.59 (11.14, 16.5) | 1.77 (1.46, 2.12) | 9.6 (4.63, 14.5) |
| **Haiti** | 99.24 (80.29, 121.02) | 1.71 (1.43, 2.06) | 205.85 (167.91, 250.1) | 1.77 (1.49, 2.12) | 3.84 (-0.54, 8.16) |
| **Honduras** | 85.35 (67.5, 106.16) | 1.93 (1.58, 2.31) | 196.47 (159.75, 240.31) | 2.06 (1.71, 2.48) | 7.1 (2.65, 12.12) |
| **Hungary** | 426.5 (365.47, 493.82) | 4.07 (3.47, 4.71) | 458.1 (391.4, 532.95) | 4.55 (3.83, 5.44) | 11.92 (2.62, 24.6) |
| **Iceland** | 24.21 (21.08, 28.12) | 8.99 (7.82, 10.43) | 38.13 (32.87, 43.98) | 8.79 (7.61, 10.08) | -2.22 (-7.96, 3.84) |
| **India** | 13132.68 (10289.18, 16693.52) | 1.58 (1.26, 1.99) | 25038.15 (19861.73, 31544.86) | 1.76 (1.4, 2.2) | 11.15 (9.45, 13.21) |
| **Indonesia** | 2532.17 (1954.91, 3259.28) | 1.41 (1.12, 1.75) | 3980.43 (3122.26, 4981.99) | 1.51 (1.2, 1.86) | 7.02 (5.4, 8.75) |
| **Iran (Islamic Republic of)** | 1362.21 (1069.19, 1695.78) | 2.67 (2.14, 3.29) | 2545.74 (2012.18, 3187) | 2.85 (2.29, 3.5) | 6.57 (4.88, 8.41) |
| **Iraq** | 378.55 (302.92, 470.01) | 2.45 (2, 3.02) | 1024.67 (821.8, 1264.44) | 2.51 (2.05, 3.08) | 2.62 (-1.23, 6.4) |
| **Ireland** | 328.23 (286.31, 373.48) | 8.42 (7.36, 9.55) | 713.46 (614.49, 821.26) | 10.96 (9.5, 12.56) | 30.09 (22.13, 37.2) |
| **Israel** | 248.29 (214.1, 284.51) | 5.11 (4.39, 5.83) | 565.98 (488.85, 648.87) | 5.58 (4.8, 6.4) | 9.25 (3.93, 14.11) |
| **Italy** | 4118.68 (3524.75, 4804.97) | 5.57 (4.78, 6.46) | 7785.12 (6656.5, 8995.95) | 7.6 (6.58, 8.8) | 36.47 (30.12, 44.47) |
| **Jamaica** | 60.69 (49.54, 73.11) | 2.62 (2.18, 3.13) | 80.5 (67.3, 95.14) | 2.79 (2.34, 3.3) | 6.59 (1.93, 11.97) |
| **Japan** | 6705.02 (5720.96, 7786.32) | 4.67 (3.95, 5.43) | 10676.26 (9166.58, 12400) | 5.32 (4.52, 6.15) | 13.81 (10.24, 17.54) |
| **Jordan** | 87.47 (68.96, 108.2) | 2.6 (2.11, 3.16) | 307.92 (247.86, 379.93) | 2.68 (2.18, 3.25) | 2.99 (-1.65, 7.19) |
| **Kazakhstan** | 465.27 (377.09, 566.23) | 2.85 (2.33, 3.44) | 542.39 (446.72, 661) | 2.97 (2.43, 3.58) | 4.02 (0.17, 8.43) |
| **Kenya** | 276.89 (212.64, 361.65) | 1.39 (1.1, 1.74) | 659.52 (509.23, 854.47) | 1.43 (1.14, 1.79) | 2.95 (2.11, 3.85) |
| **Kiribati** | 0.8 (0.63, 1.01) | 1.15 (0.93, 1.39) | 1.32 (1.05, 1.65) | 1.17 (0.95, 1.42) | 1.66 (-2.5, 5.7) |
| **Kuwait** | 56.84 (45.65, 69.35) | 3.26 (2.67, 3.88) | 151.59 (120.6, 189.62) | 3.12 (2.55, 3.79) | -4.17 (-9.8, 1.14) |
| **Kyrgyzstan** | 109.52 (88.12, 132.9) | 2.51 (2.05, 3.02) | 162.78 (131.97, 198.94) | 2.51 (2.07, 3.04) | 0.04 (-3.97, 3.98) |
| **Lao People's Democratic Republic** | 56.16 (44.75, 69.89) | 1.49 (1.22, 1.81) | 113.84 (90.81, 139.33) | 1.66 (1.35, 2) | 10.98 (6.6, 16.43) |
| **Latvia** | 96.31 (79.41, 115.79) | 3.64 (2.97, 4.37) | 76.66 (64.56, 90.24) | 3.88 (3.21, 4.62) | 6.62 (1.91, 12.22) |
| **Lebanon** | 83.41 (67.09, 101.17) | 2.73 (2.22, 3.3) | 147.79 (120.58, 180.82) | 2.81 (2.28, 3.42) | 2.83 (-0.99, 6.92) |
| **Lesotho** | 27.95 (22.39, 34.29) | 1.66 (1.37, 2) | 35.29 (28.61, 43.68) | 1.75 (1.45, 2.12) | 5.64 (1.3, 10.19) |
| **Liberia** | 22.59 (17.88, 27.99) | 1.33 (1.06, 1.64) | 56.7 (44.74, 71.43) | 1.32 (1.08, 1.62) | -0.61 (-4.35, 3.26) |
| **Libya** | 90.35 (71.81, 110.01) | 2.42 (1.97, 2.94) | 164.18 (132.34, 203.84) | 2.3 (1.87, 2.79) | -4.89 (-8.63, -0.95) |
| **Lithuania** | 135.88 (113.05, 160.57) | 3.67 (3.04, 4.37) | 145.78 (125.43, 167.49) | 4.57 (3.86, 5.3) | 24.38 (16.34, 32.48) |
| **Luxembourg** | 32.03 (27.75, 37.21) | 6.81 (5.93, 7.88) | 69.11 (59.16, 80.06) | 8.43 (7.24, 9.77) | 23.69 (17.19, 30.27) |
| **Madagascar** | 177.99 (142.75, 218.6) | 1.82 (1.49, 2.22) | 413.26 (332.11, 510.25) | 1.83 (1.5, 2.22) | 0.4 (-3.1, 3.69) |
| **Malawi** | 116.22 (92.19, 144.53) | 1.48 (1.2, 1.79) | 236.7 (186.99, 294.83) | 1.52 (1.23, 1.85) | 2.25 (-1.75, 6.34) |
| **Malaysia** | 270.65 (215.7, 340.5) | 1.62 (1.32, 1.97) | 552.47 (444.36, 677.27) | 1.75 (1.42, 2.14) | 8.03 (3.76, 12.16) |
| **Maldives** | 2.92 (2.28, 3.78) | 1.53 (1.23, 1.87) | 8.5 (6.81, 10.57) | 1.67 (1.36, 2.04) | 9.6 (5.61, 14.3) |
| **Mali** | 110.72 (87.71, 137.46) | 1.53 (1.25, 1.87) | 290.56 (231.21, 364.57) | 1.6 (1.3, 1.95) | 4.73 (1.12, 8.83) |
| **Malta** | 22 (18.87, 25.57) | 5.41 (4.66, 6.25) | 48.76 (41.74, 57.26) | 7.02 (6.1, 8.12) | 29.8 (23.75, 37.52) |
| **Marshall Islands** | 0.62 (0.48, 0.79) | 1.45 (1.18, 1.78) | 0.82 (0.66, 1.01) | 1.49 (1.22, 1.8) | 2.24 (-1.38, 5.92) |
| **Mauritania** | 32.76 (26.22, 40.41) | 1.83 (1.48, 2.22) | 67.95 (54.43, 84.74) | 1.87 (1.53, 2.28) | 2.46 (-1.46, 6.41) |
| **Mauritius** | 22.63 (18.36, 27.88) | 2.06 (1.68, 2.49) | 30.36 (25.21, 36.27) | 2.26 (1.87, 2.71) | 9.94 (5.17, 15.09) |
| **Mexico** | 2080.7 (1635.74, 2573.49) | 2.51 (2.04, 3.05) | 3461.89 (2835.97, 4184.88) | 2.74 (2.26, 3.31) | 9.38 (6.94, 12.05) |
| **Micronesia (Federated States of)** | 1.37 (1.08, 1.74) | 1.39 (1.14, 1.68) | 1.43 (1.15, 1.79) | 1.42 (1.15, 1.74) | 2.05 (-2.68, 6.81) |
| **Monaco** | 4.02 (3.41, 4.68) | 7.83 (6.75, 8.98) | 6.86 (5.85, 8.15) | 9.72 (8.4, 11.32) | 24.25 (15.51, 33.9) |
| **Mongolia** | 52.86 (42.38, 64.99) | 2.54 (2.09, 3.1) | 90.78 (73.86, 111.88) | 2.7 (2.22, 3.27) | 6.29 (1.6, 10.94) |
| **Montenegro** | 21.81 (17.76, 26.05) | 3.45 (2.81, 4.12) | 22.41 (18.59, 26.83) | 3.55 (2.91, 4.28) | 2.79 (-1.58, 7.44) |
| **Morocco** | 531.84 (425.96, 663.11) | 2.27 (1.85, 2.79) | 863.04 (701.25, 1066.94) | 2.36 (1.93, 2.89) | 3.96 (-0.01, 7.97) |
| **Mozambique** | 166 (133, 205.59) | 1.53 (1.25, 1.87) | 393.45 (313.41, 490.23) | 1.63 (1.33, 1.98) | 6.9 (2.9, 11.4) |
| **Myanmar** | 583.15 (469.43, 720.21) | 1.51 (1.23, 1.82) | 927.05 (754.96, 1129.31) | 1.71 (1.4, 2.06) | 13.41 (8.59, 18.2) |
| **Namibia** | 22.99 (18.41, 28.72) | 1.77 (1.45, 2.14) | 42.06 (33.97, 51.87) | 1.85 (1.52, 2.22) | 4.49 (0.73, 8.62) |
| **Nauru** | 0.12 (0.1, 0.16) | 1.34 (1.09, 1.64) | 0.13 (0.1, 0.16) | 1.3 (1.06, 1.59) | -3.09 (-6.82, 0.92) |
| **Nepal** | 309.7 (247.22, 384.93) | 1.72 (1.4, 2.12) | 552.41 (443.53, 676.42) | 1.83 (1.49, 2.23) | 6.32 (1.96, 10.62) |
| **Netherlands** | 1491.35 (1290.82, 1730.64) | 8.27 (7.2, 9.55) | 2741.46 (2345.83, 3194.92) | 10.11 (8.81, 11.66) | 22.24 (14, 31.31) |
| **New Zealand** | 259.91 (220.67, 300.45) | 6.94 (5.89, 8.03) | 534.19 (454.46, 621.63) | 8.34 (7.17, 9.61) | 20.22 (13.74, 27.29) |
| **Nicaragua** | 71.01 (56.05, 88.26) | 2.01 (1.62, 2.42) | 132.78 (107.07, 162.07) | 2.07 (1.69, 2.5) | 3.03 (-1.44, 7.42) |
| **Niger** | 99.1 (78.92, 123.43) | 1.54 (1.26, 1.87) | 287.31 (225.01, 355.43) | 1.56 (1.28, 1.91) | 1.42 (-2, 5.3) |
| **Nigeria** | 1143.75 (891.56, 1457.14) | 1.44 (1.14, 1.81) | 2861.93 (2203.8, 3679.65) | 1.5 (1.2, 1.88) | 4.54 (3.43, 5.64) |
| **Niue** | 0.04 (0.04, 0.05) | 1.93 (1.58, 2.31) | 0.03 (0.03, 0.04) | 1.97 (1.62, 2.38) | 2.42 (-1.73, 6.97) |
| **North Macedonia** | 68.86 (56.2, 82.72) | 3.39 (2.77, 4.06) | 73.47 (60.74, 88.73) | 3.31 (2.72, 3.99) | -2.14 (-8.85, 5.03) |
| **Northern Mariana Islands** | 0.93 (0.74, 1.16) | 2.11 (1.72, 2.53) | 0.91 (0.74, 1.1) | 2.1 (1.71, 2.52) | -0.47 (-4.42, 3.8) |
| **Norway** | 490.82 (420.8, 575.54) | 8.69 (7.52, 10.09) | 789.36 (678.37, 927.93) | 10.2 (8.81, 11.83) | 17.41 (14.56, 20.38) |
| **Oman** | 40.87 (32.3, 51.07) | 2.25 (1.81, 2.78) | 115.53 (91.14, 145.1) | 2.31 (1.87, 2.83) | 2.73 (-1.33, 6.85) |
| **Pakistan** | 1804.82 (1415.71, 2295.38) | 1.74 (1.38, 2.18) | 3959.36 (3093.03, 5030.83) | 1.82 (1.45, 2.28) | 5.05 (2.25, 8.06) |
| **Palau** | 0.24 (0.19, 0.3) | 1.59 (1.3, 1.94) | 0.3 (0.24, 0.36) | 1.61 (1.31, 1.94) | 1.26 (-2.87, 5.62) |
| **Palestine** | 43.49 (34.35, 53.94) | 2.47 (2.02, 3.01) | 119.7 (95.41, 149.94) | 2.57 (2.08, 3.16) | 4.01 (0.03, 7.74) |
| **Panama** | 48.6 (39.34, 59.82) | 2.14 (1.76, 2.55) | 97.85 (80.05, 116.48) | 2.34 (1.92, 2.78) | 9.69 (5.11, 14.78) |
| **Papua New Guinea** | 48.58 (38.47, 61.49) | 1.28 (1.04, 1.56) | 121.85 (96.76, 152.64) | 1.31 (1.07, 1.59) | 3.07 (-0.72, 6.99) |
| **Paraguay** | 98.67 (79.42, 120.44) | 2.62 (2.17, 3.15) | 192.96 (160.07, 231.47) | 2.82 (2.36, 3.35) | 7.6 (2.55, 13.14) |
| **Peru** | 364.06 (291.76, 452.65) | 1.81 (1.48, 2.19) | 648.39 (530.95, 782.96) | 1.9 (1.56, 2.29) | 4.72 (0.1, 8.86) |
| **Philippines** | 960.51 (751.67, 1222.43) | 1.6 (1.27, 1.97) | 1865.32 (1469.88, 2345.71) | 1.69 (1.35, 2.08) | 5.87 (4.83, 7.05) |
| **Poland** | 1627.6 (1354.36, 1934.56) | 4.16 (3.44, 4.94) | 2067.17 (1756.42, 2426.23) | 4.75 (3.98, 5.59) | 14.24 (11.51, 17.26) |
| **Portugal** | 550.85 (472.3, 637.07) | 4.65 (3.99, 5.35) | 1046.21 (898.19, 1216.53) | 6.41 (5.58, 7.37) | 38.09 (29.56, 47.99) |
| **Puerto Rico** | 108.13 (89.89, 128.45) | 2.97 (2.48, 3.53) | 142.75 (121.6, 165.89) | 3.47 (2.91, 4.12) | 16.93 (11.31, 23.41) |
| **Qatar** | 12.74 (10.1, 16.01) | 2.73 (2.24, 3.32) | 93.2 (72.9, 118.59) | 2.84 (2.29, 3.48) | 4.17 (-0.32, 8.66) |
| **Republic of Korea** | 1396.3 (1149.41, 1676.91) | 3.34 (2.78, 3.96) | 2794.75 (2272.37, 3390) | 4.2 (3.45, 4.99) | 25.9 (19.81, 32.84) |
| **Republic of Moldova** | 128.39 (105.32, 156.23) | 2.89 (2.37, 3.51) | 107.92 (88.92, 129.8) | 2.9 (2.37, 3.49) | 0.49 (-4.16, 5.93) |
| **Romania** | 758.72 (623.51, 913.18) | 3.22 (2.63, 3.86) | 670.4 (563.07, 793.31) | 3.39 (2.8, 4.07) | 5.29 (0.87, 9.99) |
| **Russian Federation** | 5035.41 (4101.45, 6135.21) | 3.35 (2.71, 4.05) | 5213.43 (4349.26, 6284.71) | 3.52 (2.89, 4.22) | 5.36 (3.52, 7.56) |
| **Rwanda** | 76.42 (59.78, 96.31) | 1.29 (1.04, 1.58) | 151.01 (119.94, 188.14) | 1.35 (1.1, 1.65) | 4.68 (0.92, 9.16) |
| **Saint Kitts and Nevis** | 1.02 (0.84, 1.23) | 2.47 (2.06, 2.94) | 1.69 (1.42, 2) | 2.74 (2.3, 3.25) | 11.11 (6.38, 16.56) |
| **Saint Lucia** | 3.24 (2.66, 3.93) | 2.48 (2.08, 2.96) | 5.19 (4.39, 6.06) | 2.79 (2.36, 3.27) | 12.19 (6.9, 17.75) |
| **Saint Vincent and the Grenadines** | 2.45 (1.98, 2.98) | 2.3 (1.9, 2.73) | 3.02 (2.56, 3.57) | 2.56 (2.17, 3.04) | 11.21 (6.45, 16.53) |
| **Samoa** | 2.7 (2.15, 3.4) | 1.72 (1.41, 2.09) | 3.64 (2.89, 4.5) | 1.76 (1.42, 2.13) | 2.16 (-1.72, 6.38) |
| **San Marino** | 2.21 (1.9, 2.53) | 7.55 (6.58, 8.6) | 4.49 (3.85, 5.17) | 9.02 (7.82, 10.37) | 19.49 (11.72, 28.41) |
| **Sao Tome and Principe** | 1.43 (1.14, 1.8) | 1.38 (1.11, 1.69) | 2.66 (2.1, 3.36) | 1.4 (1.14, 1.73) | 2 (-1.62, 5.9) |
| **Saudi Arabia** | 347.4 (274.75, 431.09) | 2.35 (1.92, 2.87) | 927.36 (738.38, 1158.5) | 2.42 (1.99, 2.92) | 2.85 (-0.9, 6.81) |
| **Senegal** | 102.98 (81.17, 129.29) | 1.6 (1.29, 1.96) | 221.92 (175.93, 276.6) | 1.64 (1.33, 2.01) | 2.5 (-1.72, 6.74) |
| **Serbia** | 316.59 (263.24, 379.06) | 3.32 (2.72, 3.98) | 307.31 (254.37, 368.13) | 3.45 (2.82, 4.16) | 3.74 (-1.01, 8.96) |
| **Seychelles** | 1.22 (0.99, 1.52) | 1.7 (1.39, 2.07) | 1.89 (1.55, 2.28) | 1.81 (1.48, 2.2) | 5.98 (1.97, 9.95) |
| **Sierra Leone** | 42.57 (33.91, 52.74) | 1.35 (1.1, 1.66) | 98.38 (76.81, 123.06) | 1.35 (1.1, 1.65) | 0.27 (-3.25, 4) |
| **Singapore** | 78.66 (64.5, 94.81) | 2.7 (2.25, 3.21) | 204.23 (170.3, 242.48) | 3.14 (2.61, 3.72) | 16.29 (8.96, 24.98) |
| **Slovakia** | 197.65 (162.31, 236.55) | 3.69 (3.02, 4.41) | 219.27 (182.15, 258.68) | 3.95 (3.24, 4.69) | 6.85 (2.08, 12.17) |
| **Slovenia** | 88.29 (75.31, 102.42) | 4.38 (3.74, 5.09) | 87.35 (72.17, 103.58) | 4.05 (3.32, 4.82) | -7.39 (-16.47, 1.14) |
| **Solomon Islands** | 4.07 (3.16, 5.15) | 1.25 (1.02, 1.51) | 8.09 (6.36, 10.05) | 1.29 (1.04, 1.55) | 2.74 (-1.51, 7.09) |
| **Somalia** | 72.89 (56.98, 91.54) | 1.25 (1.01, 1.53) | 202.39 (159.01, 254.71) | 1.22 (0.98, 1.5) | -2.12 (-5.75, 1.98) |
| **South Africa** | 675.5 (534.97, 852.65) | 1.9 (1.53, 2.34) | 1111.24 (889.39, 1378.93) | 2 (1.61, 2.46) | 5.66 (4.06, 7.41) |
| **South Sudan** | 80.66 (64.06, 101.18) | 1.7 (1.39, 2.07) | 129.07 (102.26, 161.68) | 1.71 (1.38, 2.07) | 0.25 (-3.13, 4.21) |
| **Spain** | 2615.43 (2255.49, 3019.86) | 5.54 (4.79, 6.32) | 4976.3 (4252.1, 5738.74) | 7.06 (6.08, 8.13) | 27.48 (19.64, 36.71) |
| **Sri Lanka** | 277.62 (222.84, 345.31) | 1.65 (1.34, 2.01) | 408.36 (331.62, 490.09) | 1.82 (1.48, 2.2) | 10.16 (5.1, 14.82) |
| **Sudan** | 285.11 (223.17, 356.28) | 1.62 (1.3, 2.01) | 637.04 (501.65, 799.36) | 1.68 (1.37, 2.07) | 4.05 (0.39, 8.21) |
| **Suriname** | 7.07 (5.78, 8.64) | 1.89 (1.57, 2.26) | 12.02 (10.1, 14.22) | 2.05 (1.72, 2.43) | 8.81 (3.95, 13.54) |
| **Sweden** | 1225.77 (1054.51, 1428.61) | 9.88 (8.56, 11.48) | 1747.17 (1501.74, 2031.12) | 10.95 (9.4, 12.73) | 10.78 (5.45, 16.96) |
| **Switzerland** | 712.03 (611.86, 821.18) | 7.98 (6.91, 9.17) | 1185.26 (1013.67, 1376.11) | 8.79 (7.57, 10.11) | 10.24 (4.36, 16.48) |
| **Syrian Arab Republic** | 286.02 (226.29, 354.63) | 2.53 (2.07, 3.1) | 366.95 (297.19, 452.09) | 2.54 (2.07, 3.12) | 0.43 (-3.38, 4.57) |
| **Taiwan (Province of China)** | 608.57 (503.05, 731.11) | 2.98 (2.47, 3.54) | 863.35 (725.14, 1004.11) | 3.3 (2.75, 3.88) | 10.54 (4.93, 16.11) |
| **Tajikistan** | 119.94 (95.64, 148.29) | 2.39 (1.94, 2.91) | 218.93 (174.95, 270.79) | 2.35 (1.91, 2.87) | -1.7 (-5.97, 2.35) |
| **Thailand** | 1006.1 (812.21, 1248.7) | 1.81 (1.48, 2.2) | 1515.01 (1230.91, 1847.14) | 1.99 (1.62, 2.4) | 9.89 (4.99, 14.59) |
| **Timor-Leste** | 9.84 (7.77, 12.42) | 1.42 (1.14, 1.73) | 18.85 (15.09, 23.48) | 1.52 (1.24, 1.84) | 7.08 (2.89, 11.38) |
| **Togo** | 42.56 (33.38, 53.68) | 1.38 (1.11, 1.67) | 98.98 (78.04, 123.78) | 1.39 (1.12, 1.7) | 1.09 (-2.74, 5.08) |
| **Tokelau** | 0.03 (0.02, 0.03) | 1.57 (1.28, 1.92) | 0.02 (0.02, 0.03) | 1.66 (1.35, 2.01) | 5.35 (0.41, 10.35) |
| **Tonga** | 1.78 (1.41, 2.19) | 1.93 (1.57, 2.31) | 1.99 (1.6, 2.43) | 2 (1.63, 2.42) | 3.68 (-0.3, 8.1) |
| **Trinidad and Tobago** | 26.51 (21.63, 32.06) | 2.27 (1.88, 2.72) | 36.63 (31.05, 43.09) | 2.48 (2.07, 2.94) | 9.01 (4.19, 14.87) |
| **Tunisia** | 206.32 (164.3, 252.95) | 2.63 (2.14, 3.19) | 332.41 (268.6, 408.11) | 2.74 (2.21, 3.35) | 4.09 (-0.53, 8.05) |
| **Turkey** | 1784.89 (1466.94, 2128.1) | 3.26 (2.7, 3.85) | 3163.21 (2636.72, 3746.44) | 3.6 (3.02, 4.22) | 10.22 (6.21, 14.49) |
| **Turkmenistan** | 85.35 (68.37, 104.74) | 2.41 (1.97, 2.9) | 128.14 (104.32, 155.82) | 2.54 (2.08, 3.06) | 5.4 (1.09, 9.61) |
| **Tuvalu** | 0.12 (0.1, 0.15) | 1.34 (1.09, 1.63) | 0.17 (0.13, 0.2) | 1.41 (1.15, 1.72) | 5.38 (0.75, 10.07) |
| **Uganda** | 174.27 (135.76, 219.9) | 1.24 (1.01, 1.51) | 442.41 (347.7, 561.38) | 1.31 (1.06, 1.61) | 5.4 (1.12, 9.58) |
| **Ukraine** | 1590.31 (1292.4, 1934.66) | 3.07 (2.49, 3.72) | 1304.85 (1081, 1575.04) | 3.02 (2.46, 3.65) | -1.72 (-6.23, 2.76) |
| **United Arab Emirates** | 51.99 (41.16, 65.54) | 2.68 (2.19, 3.28) | 297.48 (228.9, 383.57) | 2.67 (2.19, 3.21) | -0.54 (-4.43, 4.52) |
| **United Kingdom** | 5824 (5014.62, 6790.41) | 7.6 (6.58, 8.77) | 9864.69 (8485.61, 11431.62) | 9.66 (8.34, 11.18) | 27.18 (23.94, 30.52) |
| **United Republic of Tanzania** | 306.43 (242.87, 383.2) | 1.44 (1.17, 1.75) | 717.72 (565.01, 893.23) | 1.5 (1.21, 1.84) | 4.36 (0.53, 8.52) |
| **United States of America** | 21215.81 (18514.75, 24107.5) | 7.42 (6.5, 8.41) | 37732.59 (35136.67, 40389.84) | 8.5 (7.91, 9.09) | 14.6 (4.92, 26.42) |
| **United States Virgin Islands** | 3.28 (2.8, 3.84) | 3.24 (2.78, 3.77) | 5.33 (4.54, 6.15) | 4 (3.43, 4.6) | 23.65 (17.76, 29.99) |
| **Uruguay** | 145.57 (124.99, 166.95) | 4.3 (3.71, 4.94) | 214.7 (184.82, 244.58) | 5.21 (4.47, 5.96) | 21.33 (15.44, 27.02) |
| **Uzbekistan** | 498.44 (399.01, 608.31) | 2.48 (2.03, 3.01) | 866.2 (686.9, 1065.85) | 2.58 (2.09, 3.13) | 4.16 (-0.02, 8.75) |
| **Vanuatu** | 2.27 (1.78, 2.84) | 1.61 (1.31, 1.94) | 4.65 (3.74, 5.79) | 1.63 (1.34, 1.97) | 1.73 (-2.23, 6.25) |
| **Venezuela (Bolivarian Republic of)** | 363.44 (290.91, 449.65) | 2.06 (1.69, 2.46) | 613.9 (509.6, 733.37) | 2.15 (1.78, 2.57) | 4.47 (0.28, 8.78) |
| **Viet Nam** | 1115.3 (892.25, 1377.31) | 1.72 (1.41, 2.09) | 1872.01 (1530.38, 2269.95) | 1.9 (1.56, 2.29) | 10.34 (6.11, 15) |
| **Yemen** | 185.87 (145.77, 232.83) | 1.63 (1.32, 2.01) | 470.37 (372.48, 588.04) | 1.62 (1.3, 2) | -0.76 (-4.46, 3.15) |
| **Zambia** | 101.22 (79.9, 127.12) | 1.54 (1.26, 1.88) | 249.34 (197.27, 312.79) | 1.61 (1.31, 1.97) | 4.42 (1.15, 8.28) |
| **Zimbabwe** | 142.79 (112.47, 178.89) | 1.51 (1.24, 1.83) | 206.92 (164.21, 261.41) | 1.47 (1.21, 1.78) | -2.86 (-6.47, 1.32) |
| **Incidence** | | | | | |
| **Afghanistan** | 74.54 (60.39, 90.87) | 0.68 (0.57, 0.82) | 226.12 (182.49, 278.09) | 0.69 (0.57, 0.84) | 2.14 (-1, 5.84) |
| **Albania** | 14.16 (11.87, 16.63) | 0.49 (0.41, 0.58) | 15.35 (12.91, 18.5) | 0.48 (0.41, 0.57) | -0.95 (-4.01, 3.09) |
| **Algeria** | 113.63 (92.65, 136.91) | 0.52 (0.43, 0.62) | 210.88 (174.24, 254.38) | 0.52 (0.43, 0.62) | 0.8 (-2.78, 4.51) |
| **American Samoa** | 0.16 (0.13, 0.18) | 0.46 (0.4, 0.54) | 0.21 (0.17, 0.25) | 0.41 (0.34, 0.49) | -11.85 (-16.92, -7.18) |
| **Andorra** | 1.21 (1.09, 1.31) | 2.2 (2.01, 2.37) | 3.16 (2.87, 3.45) | 2.46 (2.24, 2.66) | 11.86 (8.58, 15.22) |
| **Angola** | 44.2 (36.17, 53.8) | 0.67 (0.55, 0.84) | 123.76 (100.69, 150.19) | 0.65 (0.53, 0.82) | -2.37 (-5.53, 1.18) |
| **Antigua and Barbuda** | 0.28 (0.25, 0.33) | 0.51 (0.44, 0.6) | 0.5 (0.43, 0.6) | 0.54 (0.47, 0.63) | 5 (1.23, 8.66) |
| **Argentina** | 253.7 (221.94, 285.71) | 0.77 (0.68, 0.87) | 448.08 (401.08, 495.81) | 0.9 (0.8, 1) | 16.18 (11.55, 20.88) |
| **Armenia** | 14.64 (12.04, 17.75) | 0.47 (0.39, 0.57) | 15.41 (12.68, 18.79) | 0.45 (0.38, 0.54) | -2.99 (-6.56, 0.53) |
| **Australia** | 388.29 (369.78, 408.1) | 2.07 (1.97, 2.18) | 936.93 (894.34, 975.95) | 2.48 (2.37, 2.59) | 19.97 (16.94, 22.98) |
| **Austria** | 115.13 (105.54, 125.63) | 1.2 (1.09, 1.31) | 222.21 (207.36, 236.45) | 1.57 (1.46, 1.68) | 31.17 (25.16, 36.66) |
| **Azerbaijan** | 30.42 (25.24, 36.64) | 0.47 (0.4, 0.57) | 46.16 (37.28, 57.63) | 0.46 (0.38, 0.56) | -2.24 (-5.69, 1.49) |
| **Bahamas** | 1.38 (1.2, 1.56) | 0.7 (0.62, 0.79) | 2.63 (2.3, 3.02) | 0.71 (0.62, 0.8) | 0.38 (-2.66, 3.55) |
| **Bahrain** | 1.93 (1.59, 2.36) | 0.46 (0.39, 0.56) | 6.85 (5.38, 8.89) | 0.48 (0.4, 0.59) | 4.35 (0.56, 8.32) |
| **Bangladesh** | 371.1 (306.51, 443.54) | 0.44 (0.36, 0.54) | 626.39 (515.51, 767) | 0.43 (0.36, 0.52) | -1.84 (-4.86, 1.92) |
| **Barbados** | 2.48 (2.28, 2.68) | 0.95 (0.86, 1.03) | 4.37 (4, 4.72) | 1.03 (0.95, 1.12) | 9.37 (6.53, 12.68) |
| **Belarus** | 58.82 (48.75, 71.07) | 0.53 (0.45, 0.63) | 60.41 (50.68, 73.18) | 0.52 (0.44, 0.62) | -1.28 (-4.6, 2.25) |
| **Belgium** | 195.4 (182.47, 208.41) | 1.48 (1.37, 1.59) | 353.96 (333.67, 372.77) | 1.88 (1.76, 1.99) | 27.06 (22.55, 31.5) |
| **Belize** | 0.83 (0.69, 0.97) | 0.55 (0.47, 0.65) | 2.01 (1.72, 2.33) | 0.59 (0.51, 0.68) | 7.5 (2.74, 12.19) |
| **Benin** | 18.03 (14.78, 21.66) | 0.53 (0.44, 0.65) | 45.31 (37.01, 53.97) | 0.53 (0.44, 0.65) | 0.31 (-3.45, 3.68) |
| **Bermuda** | 0.67 (0.61, 0.73) | 1.11 (1.01, 1.2) | 1.03 (0.94, 1.13) | 1 (0.9, 1.09) | -9.65 (-12.33, -6.73) |
| **Bhutan** | 2.06 (1.68, 2.47) | 0.44 (0.37, 0.54) | 2.88 (2.37, 3.54) | 0.43 (0.36, 0.52) | -2.98 (-6.11, 0.29) |
| **Bolivia (Plurinational State of)** | 26.19 (22, 30.11) | 0.52 (0.44, 0.6) | 61.76 (53.14, 70.28) | 0.59 (0.51, 0.67) | 13.53 (9.16, 17.6) |
| **Bosnia and Herzegovina** | 24.19 (19.82, 29.8) | 0.56 (0.47, 0.69) | 23.2 (18.88, 29.55) | 0.54 (0.44, 0.66) | -4.57 (-7.88, -0.84) |
| **Botswana** | 5 (4.12, 5.99) | 0.57 (0.47, 0.69) | 10.21 (8.29, 12.68) | 0.54 (0.45, 0.66) | -3.67 (-6.77, -0.42) |
| **Brazil** | 760.4 (655.71, 875.53) | 0.62 (0.54, 0.71) | 1877.33 (1672.09, 2089) | 0.83 (0.74, 0.92) | 33.47 (27.61, 39.46) |
| **Brunei Darussalam** | 0.72 (0.58, 0.88) | 0.48 (0.39, 0.6) | 1.7 (1.37, 2.13) | 0.48 (0.39, 0.6) | 0.4 (-3.17, 4.03) |
| **Bulgaria** | 52.55 (43.65, 64.02) | 0.55 (0.46, 0.65) | 50.87 (41.85, 62.99) | 0.56 (0.47, 0.67) | 2.03 (-2.26, 6.29) |
| **Burkina Faso** | 39.59 (32.6, 47.49) | 0.57 (0.48, 0.71) | 89.3 (73.25, 106.83) | 0.57 (0.47, 0.7) | -0.44 (-3.47, 3.1) |
| **Burundi** | 23.49 (19.32, 28.48) | 0.65 (0.53, 0.81) | 50.64 (41.15, 61.76) | 0.68 (0.56, 0.85) | 4.93 (1.09, 8.95) |
| **Cabo Verde** | 1.44 (1.21, 1.72) | 0.53 (0.44, 0.65) | 2.44 (2.03, 3) | 0.5 (0.42, 0.62) | -5.43 (-8.98, -2.07) |
| **Cambodia** | 35.95 (29.39, 42.98) | 0.47 (0.39, 0.57) | 65.43 (53.93, 79.7) | 0.46 (0.38, 0.56) | -1.82 (-5.03, 1.77) |
| **Cameroon** | 36.14 (29.37, 43.42) | 0.49 (0.41, 0.6) | 97.8 (80.61, 118.79) | 0.5 (0.41, 0.61) | 1.25 (-1.81, 4.67) |
| **Canada** | 609.76 (574.53, 647.88) | 1.98 (1.87, 2.1) | 1365.37 (1291.94, 1437.05) | 2.26 (2.13, 2.39) | 13.88 (10.86, 17.29) |
| **Central African Republic** | 12.74 (10.42, 15.59) | 0.71 (0.58, 0.88) | 25.12 (20.46, 31.07) | 0.74 (0.6, 0.93) | 4.26 (0.77, 8.15) |
| **Chad** | 26.52 (21.9, 31.58) | 0.6 (0.5, 0.74) | 64.18 (52.47, 77.18) | 0.6 (0.49, 0.73) | -1.17 (-4.55, 2.06) |
| **Chile** | 107.77 (95.84, 119.95) | 0.95 (0.85, 1.05) | 228.84 (207.96, 249.8) | 1.05 (0.95, 1.14) | 10.42 (6.49, 14.62) |
| **China** | 6043.49 (5021.73, 7303.41) | 0.59 (0.5, 0.72) | 9015.39 (7453.57, 11205.53) | 0.54 (0.45, 0.64) | -9.63 (-11.63, -7.56) |
| **Colombia** | 129.53 (109.69, 150.25) | 0.47 (0.4, 0.54) | 285.31 (251.43, 323.67) | 0.58 (0.51, 0.66) | 23.78 (16.86, 30.73) |
| **Comoros** | 2.04 (1.67, 2.48) | 0.65 (0.53, 0.82) | 3.82 (3.14, 4.72) | 0.67 (0.55, 0.83) | 2.75 (-0.61, 6.64) |
| **Congo** | 8.91 (7.27, 10.81) | 0.56 (0.46, 0.7) | 21.4 (17.37, 26.71) | 0.56 (0.46, 0.7) | 0.27 (-3.22, 3.99) |
| **Cook Islands** | 0.07 (0.06, 0.08) | 0.45 (0.38, 0.53) | 0.09 (0.08, 0.11) | 0.42 (0.36, 0.5) | -6.38 (-9.76, -2.94) |
| **Costa Rica** | 17.22 (15.34, 19.22) | 0.76 (0.69, 0.84) | 55.06 (50.81, 59.18) | 1.11 (1.03, 1.19) | 45.48 (39.81, 51.69) |
| **Croatia** | 36.6 (31.72, 41.74) | 0.7 (0.61, 0.79) | 46.41 (41.53, 51.7) | 0.76 (0.68, 0.85) | 9.07 (4.66, 13.76) |
| **Cuba** | 71.78 (63.31, 80.64) | 0.71 (0.63, 0.8) | 121.84 (109.02, 135.49) | 0.79 (0.71, 0.87) | 10.67 (7.25, 14.34) |
| **Cyprus** | 7.91 (6.93, 8.94) | 0.97 (0.86, 1.1) | 19.09 (17.04, 21.22) | 1.1 (0.98, 1.21) | 12.81 (8.11, 16.87) |
| **Czechia** | 72.62 (63.34, 83.45) | 0.66 (0.58, 0.76) | 106.68 (93.83, 120.23) | 0.71 (0.62, 0.8) | 6.68 (2.59, 11.41) |
| **Côte d'Ivoire** | 41.38 (33.18, 49.87) | 0.52 (0.43, 0.63) | 95.21 (77.34, 115.42) | 0.52 (0.43, 0.64) | 1.59 (-1.96, 5.17) |
| **Democratic People's Republic of Korea** | 140.07 (117.2, 167.29) | 0.72 (0.61, 0.86) | 229.13 (190.6, 284.69) | 0.78 (0.66, 0.95) | 8.68 (4.99, 12.64) |
| **Democratic Republic of the Congo** | 154.17 (125.24, 188.54) | 0.62 (0.5, 0.77) | 357.88 (292.19, 438.73) | 0.65 (0.53, 0.81) | 5.14 (1.27, 8.69) |
| **Denmark** | 133.33 (124.6, 142.29) | 1.87 (1.73, 2) | 195.51 (182.36, 207.95) | 1.97 (1.83, 2.1) | 5.44 (2.38, 8.57) |
| **Djibouti** | 1.78 (1.46, 2.16) | 0.63 (0.52, 0.78) | 5.58 (4.53, 6.95) | 0.63 (0.52, 0.79) | 0.12 (-3.38, 3.8) |
| **Dominica** | 0.49 (0.44, 0.54) | 0.69 (0.61, 0.77) | 0.73 (0.66, 0.8) | 0.95 (0.86, 1.04) | 37.41 (30.15, 44.27) |
| **Dominican Republic** | 31.98 (26.45, 37.81) | 0.54 (0.45, 0.65) | 54.77 (46.24, 64.44) | 0.54 (0.46, 0.63) | -0.58 (-6, 4.04) |
| **Ecuador** | 28.96 (24.03, 34.08) | 0.37 (0.31, 0.44) | 75.02 (64.64, 86.34) | 0.47 (0.4, 0.53) | 25.94 (19.13, 34.04) |
| **Egypt** | 279.61 (229.27, 338.85) | 0.56 (0.46, 0.67) | 482.15 (399.82, 580.37) | 0.55 (0.46, 0.66) | -1.81 (-5.17, 1.96) |
| **El Salvador** | 20.96 (17.19, 24.82) | 0.48 (0.39, 0.57) | 27.22 (22.77, 32.44) | 0.46 (0.39, 0.55) | -3.08 (-6.82, 0.02) |
| **Equatorial Guinea** | 1.81 (1.47, 2.2) | 0.61 (0.5, 0.76) | 4.26 (3.48, 5.21) | 0.52 (0.42, 0.64) | -15.16 (-18.53, -11.2) |
| **Eritrea** | 13.18 (10.85, 15.99) | 0.74 (0.61, 0.92) | 30.84 (25.14, 37.86) | 0.73 (0.59, 0.91) | -1.54 (-5.13, 1.88) |
| **Estonia** | 9.38 (7.95, 11.1) | 0.55 (0.47, 0.64) | 8.67 (7.33, 10.4) | 0.53 (0.44, 0.63) | -3.29 (-6.94, -0.14) |
| **Eswatini** | 3.25 (2.67, 3.91) | 0.6 (0.5, 0.73) | 4.98 (4.15, 6.04) | 0.59 (0.5, 0.73) | -0.49 (-4.4, 3.06) |
| **Ethiopia** | 224.33 (183.41, 275.58) | 0.67 (0.55, 0.85) | 454.32 (373.25, 560.78) | 0.66 (0.54, 0.84) | -1.03 (-2.83, 0.78) |
| **Fiji** | 2.45 (2.02, 2.96) | 0.44 (0.37, 0.54) | 3.75 (3.05, 4.57) | 0.44 (0.37, 0.54) | 0.45 (-2.48, 4.1) |
| **Finland** | 155.77 (144.28, 167.77) | 2.35 (2.18, 2.53) | 255.36 (238.51, 273.22) | 2.52 (2.35, 2.69) | 6.96 (3.83, 10.48) |
| **France** | 1347.96 (1278.6, 1416.68) | 1.85 (1.75, 1.95) | 2189.67 (2072.81, 2302.62) | 2 (1.87, 2.12) | 8.14 (4.53, 11.85) |
| **Gabon** | 3.65 (3, 4.49) | 0.5 (0.41, 0.64) | 6.79 (5.47, 8.47) | 0.51 (0.42, 0.64) | 0.49 (-3.32, 4.81) |
| **Gambia** | 3.59 (2.9, 4.31) | 0.55 (0.45, 0.68) | 8.31 (6.85, 9.98) | 0.55 (0.46, 0.68) | 0.17 (-3.05, 3.6) |
| **Georgia** | 25.52 (21.16, 31.15) | 0.45 (0.38, 0.55) | 20.6 (17.26, 24.98) | 0.46 (0.39, 0.56) | 2.11 (-0.93, 5.15) |
| **Germany** | 1394.35 (1294.99, 1489.18) | 1.28 (1.18, 1.38) | 2462.02 (2320.46, 2593.32) | 1.61 (1.51, 1.72) | 25.87 (21.67, 30.18) |
| **Ghana** | 52.26 (42.75, 62.91) | 0.5 (0.42, 0.61) | 112.85 (92.98, 138.13) | 0.49 (0.41, 0.6) | -2.85 (-7, 1.31) |
| **Greece** | 94.36 (81.82, 108.93) | 0.77 (0.67, 0.89) | 167.24 (152.83, 183.99) | 1.06 (0.95, 1.16) | 36.99 (27.49, 46.06) |
| **Greenland** | 0.69 (0.58, 0.82) | 1.68 (1.43, 1.98) | 1.12 (0.94, 1.36) | 1.69 (1.45, 2.02) | 0.83 (-3.08, 4.6) |
| **Grenada** | 0.43 (0.38, 0.49) | 0.57 (0.5, 0.66) | 0.74 (0.65, 0.83) | 0.68 (0.6, 0.76) | 18.68 (13.32, 24.16) |
| **Guam** | 0.73 (0.64, 0.82) | 0.76 (0.68, 0.85) | 0.73 (0.61, 0.86) | 0.39 (0.33, 0.47) | -48.47 (-53.23, -43.55) |
| **Guatemala** | 35.66 (28.93, 43.07) | 0.51 (0.42, 0.61) | 74.68 (61.95, 88.72) | 0.5 (0.42, 0.61) | -0.98 (-4.86, 3.4) |
| **Guinea** | 26.35 (21.64, 31.81) | 0.55 (0.46, 0.68) | 48.52 (39.94, 57.67) | 0.55 (0.46, 0.68) | 0.46 (-3.31, 4.82) |
| **Guinea-Bissau** | 3.97 (3.27, 4.8) | 0.58 (0.48, 0.72) | 7.31 (6.06, 8.82) | 0.59 (0.49, 0.72) | 1.9 (-1.6, 5.69) |
| **Guyana** | 3.14 (2.62, 3.67) | 0.53 (0.45, 0.62) | 3.9 (3.33, 4.51) | 0.56 (0.48, 0.64) | 4.87 (0.99, 8.77) |
| **Haiti** | 37.85 (32.2, 43.39) | 0.68 (0.59, 0.79) | 75.85 (65.43, 87) | 0.75 (0.66, 0.85) | 9.95 (6.34, 13.84) |
| **Honduras** | 20.47 (17.01, 24.36) | 0.5 (0.43, 0.59) | 44.58 (38.08, 51.55) | 0.55 (0.47, 0.63) | 8.69 (4.26, 14.03) |
| **Hungary** | 82.83 (72.84, 94.18) | 0.74 (0.67, 0.84) | 101.51 (90.1, 113.83) | 0.77 (0.68, 0.86) | 2.83 (-1.66, 8.5) |
| **Iceland** | 4.78 (4.47, 5.11) | 1.78 (1.66, 1.91) | 6.46 (5.89, 7.01) | 1.41 (1.28, 1.54) | -20.8 (-23.96, -17.45) |
| **India** | 3027.7 (2473.16, 3707.18) | 0.42 (0.34, 0.52) | 5280.13 (4312.28, 6482.6) | 0.41 (0.34, 0.5) | -2.21 (-3.85, -0.46) |
| **Indonesia** | 541.29 (440.85, 656.89) | 0.37 (0.31, 0.46) | 897.74 (717.56, 1119.83) | 0.37 (0.3, 0.45) | -1.73 (-2.89, -0.34) |
| **Iran (Islamic Republic of)** | 288.82 (237.83, 343.13) | 0.57 (0.48, 0.69) | 490.34 (409.3, 596.04) | 0.6 (0.51, 0.71) | 4.1 (2.19, 6.15) |
| **Iraq** | 92.55 (74.86, 111.58) | 0.58 (0.48, 0.71) | 215.07 (177.87, 260.45) | 0.6 (0.5, 0.73) | 4.14 (0.66, 7.67) |
| **Ireland** | 82.73 (78.53, 86.94) | 2.12 (2.01, 2.22) | 173.21 (163.81, 182.55) | 2.53 (2.4, 2.67) | 19.76 (16.31, 23.15) |
| **Israel** | 55.19 (50.52, 59.76) | 1.14 (1.04, 1.24) | 117.42 (107.65, 126.75) | 1.11 (1.01, 1.2) | -3.25 (-6.12, -0.34) |
| **Italy** | 978.58 (908.69, 1052.24) | 1.34 (1.24, 1.43) | 1900.75 (1798.3, 1999.04) | 1.69 (1.57, 1.79) | 26.1 (22.15, 30.61) |
| **Jamaica** | 12.46 (10.7, 14.26) | 0.6 (0.52, 0.7) | 17.4 (15.1, 19.88) | 0.64 (0.56, 0.73) | 5.92 (1.87, 10.67) |
| **Japan** | 1321.51 (1191.89, 1454.82) | 0.84 (0.76, 0.92) | 2520.94 (2338.78, 2711.13) | 0.93 (0.85, 1.01) | 10.26 (8.43, 12.27) |
| **Jordan** | 17.05 (13.76, 20.76) | 0.53 (0.44, 0.64) | 54.74 (45.63, 66.1) | 0.54 (0.46, 0.65) | 2.42 (-1.92, 6.62) |
| **Kazakhstan** | 76.56 (64.01, 91.79) | 0.51 (0.43, 0.61) | 92.43 (76.95, 112.46) | 0.5 (0.42, 0.6) | -1.77 (-4.64, 1.12) |
| **Kenya** | 79.45 (64.26, 97.49) | 0.55 (0.45, 0.69) | 191.27 (153.83, 238.82) | 0.56 (0.46, 0.71) | 1.83 (1.04, 2.66) |
| **Kiribati** | 0.24 (0.2, 0.28) | 0.43 (0.36, 0.51) | 0.42 (0.35, 0.5) | 0.45 (0.38, 0.53) | 3.68 (0.25, 7.03) |
| **Kuwait** | 7.81 (6.55, 9.31) | 0.54 (0.46, 0.63) | 18.93 (15.12, 23.94) | 0.47 (0.4, 0.57) | -11.96 (-16.45, -7.43) |
| **Kyrgyzstan** | 19.07 (16.06, 22.44) | 0.48 (0.4, 0.58) | 29.27 (24.32, 35.27) | 0.5 (0.41, 0.6) | 3.33 (0.23, 6.9) |
| **Lao People's Democratic Republic** | 16.04 (13.25, 19.17) | 0.5 (0.42, 0.61) | 28.16 (23.3, 33.98) | 0.49 (0.41, 0.59) | -2.65 (-5.59, 1.2) |
| **Latvia** | 15.58 (13, 18.91) | 0.53 (0.44, 0.64) | 15.21 (13.05, 17.76) | 0.59 (0.5, 0.68) | 10.62 (4.28, 17.94) |
| **Lebanon** | 16.95 (13.94, 20.1) | 0.53 (0.45, 0.64) | 27.75 (23.07, 33.48) | 0.53 (0.44, 0.64) | -0.55 (-3.8, 2.8) |
| **Lesotho** | 9.2 (7.68, 10.96) | 0.69 (0.57, 0.83) | 10.71 (8.85, 13.22) | 0.66 (0.55, 0.81) | -4.11 (-7.36, -1.07) |
| **Liberia** | 7.91 (6.49, 9.57) | 0.52 (0.43, 0.64) | 17.94 (14.72, 21.97) | 0.54 (0.45, 0.66) | 3.8 (0.58, 6.98) |
| **Libya** | 17.55 (14.38, 20.99) | 0.47 (0.4, 0.56) | 28.97 (24.06, 35.4) | 0.48 (0.4, 0.57) | 1.41 (-2.72, 5.53) |
| **Lithuania** | 21.49 (18.19, 25.34) | 0.55 (0.46, 0.64) | 36.56 (33.18, 40.33) | 0.86 (0.77, 0.95) | 57.88 (45.08, 70.86) |
| **Luxembourg** | 7.05 (6.52, 7.6) | 1.45 (1.34, 1.57) | 14.25 (13.29, 15.26) | 1.65 (1.53, 1.76) | 13.24 (9.8, 16.68) |
| **Madagascar** | 58.02 (48.08, 69.9) | 0.75 (0.61, 0.93) | 130.36 (107.02, 160.49) | 0.76 (0.63, 0.97) | 2.52 (-1.16, 6.34) |
| **Malawi** | 45.93 (37.68, 55.84) | 0.75 (0.62, 0.94) | 84.96 (69.94, 104.62) | 0.76 (0.62, 0.95) | 1.02 (-2.39, 4.39) |
| **Malaysia** | 45.67 (37.41, 55.41) | 0.35 (0.29, 0.43) | 101.04 (82.75, 125.39) | 0.35 (0.29, 0.43) | 0.13 (-3.06, 3.15) |
| **Maldives** | 0.58 (0.46, 0.71) | 0.37 (0.31, 0.46) | 1.5 (1.21, 1.87) | 0.37 (0.3, 0.45) | -1.38 (-4.78, 2.33) |
| **Mali** | 39.06 (32.45, 47.15) | 0.61 (0.51, 0.75) | 89.81 (74.64, 107.54) | 0.61 (0.5, 0.75) | -0.09 (-2.88, 3.15) |
| **Malta** | 5.33 (4.92, 5.77) | 1.3 (1.2, 1.41) | 12.35 (11.54, 13.18) | 1.59 (1.47, 1.7) | 22.05 (18.04, 26.04) |
| **Marshall Islands** | 0.14 (0.11, 0.16) | 0.47 (0.4, 0.55) | 0.21 (0.18, 0.25) | 0.46 (0.39, 0.55) | -1.01 (-3.98, 2.23) |
| **Mauritania** | 8.5 (7.04, 10.11) | 0.56 (0.47, 0.69) | 16.26 (13.57, 19.62) | 0.56 (0.47, 0.69) | 0.05 (-3.09, 3.38) |
| **Mauritius** | 3.78 (3.13, 4.52) | 0.42 (0.35, 0.5) | 6.72 (5.62, 8.14) | 0.43 (0.37, 0.52) | 2.61 (-1.47, 6.55) |
| **Mexico** | 389.29 (328.94, 453.23) | 0.53 (0.45, 0.62) | 732.59 (630.8, 843.85) | 0.61 (0.53, 0.7) | 14.7 (11.14, 18.03) |
| **Micronesia (Federated States of)** | 0.33 (0.27, 0.39) | 0.45 (0.38, 0.53) | 0.38 (0.31, 0.46) | 0.44 (0.37, 0.53) | -1.67 (-5.28, 2.02) |
| **Monaco** | 0.91 (0.85, 0.96) | 1.66 (1.55, 1.76) | 1.52 (1.44, 1.6) | 1.96 (1.84, 2.09) | 18.2 (14.32, 22.38) |
| **Mongolia** | 9.91 (8.23, 11.73) | 0.57 (0.48, 0.7) | 17.58 (14.5, 21.85) | 0.55 (0.46, 0.67) | -3.19 (-6.34, 0.33) |
| **Montenegro** | 3.04 (2.52, 3.7) | 0.49 (0.41, 0.6) | 3.85 (3.19, 4.81) | 0.51 (0.43, 0.62) | 3.75 (0.18, 7.75) |
| **Morocco** | 130.62 (106.55, 158.1) | 0.58 (0.48, 0.71) | 195.19 (160.78, 239.05) | 0.57 (0.48, 0.69) | -2.05 (-5.8, 1.62) |
| **Mozambique** | 72.08 (59.11, 88.31) | 0.81 (0.66, 1.02) | 140.33 (115.33, 169.85) | 0.78 (0.64, 0.97) | -4.17 (-7.48, -0.89) |
| **Myanmar** | 165.66 (137.71, 199.49) | 0.51 (0.43, 0.62) | 248.56 (205.89, 304.88) | 0.49 (0.41, 0.59) | -4.73 (-7.89, -1.39) |
| **Namibia** | 5.52 (4.6, 6.58) | 0.54 (0.45, 0.66) | 10.1 (8.31, 12.29) | 0.54 (0.45, 0.66) | 0.35 (-3.04, 4.11) |
| **Nauru** | 0.03 (0.02, 0.03) | 0.4 (0.34, 0.47) | 0.03 (0.02, 0.04) | 0.39 (0.33, 0.47) | -2.28 (-6.24, 2.44) |
| **Nepal** | 76.41 (62.68, 91.42) | 0.48 (0.39, 0.58) | 123.77 (102.19, 149.94) | 0.47 (0.39, 0.57) | -1.75 (-5.15, 2.35) |
| **Netherlands** | 366.03 (346.59, 384.67) | 2.01 (1.91, 2.11) | 673.79 (638.74, 707.83) | 2.27 (2.15, 2.4) | 12.9 (9.26, 16.38) |
| **New Zealand** | 83.68 (79.13, 88.11) | 2.2 (2.08, 2.32) | 171.6 (162.09, 180.65) | 2.44 (2.29, 2.57) | 10.86 (8.05, 13.93) |
| **Nicaragua** | 14.52 (11.78, 17.64) | 0.46 (0.38, 0.55) | 25.76 (21.29, 30.68) | 0.46 (0.38, 0.55) | 0.4 (-2.93, 4) |
| **Niger** | 33.29 (27.1, 39.98) | 0.61 (0.51, 0.75) | 95.19 (78.1, 114.7) | 0.63 (0.52, 0.78) | 3.09 (-0.36, 6.32) |
| **Nigeria** | 326.45 (268.54, 394.43) | 0.47 (0.39, 0.59) | 722.67 (592.01, 875.29) | 0.47 (0.39, 0.58) | -1.42 (-2.19, -0.53) |
| **Niue** | 0.01 (0.01, 0.01) | 0.47 (0.4, 0.55) | 0.01 (0.01, 0.01) | 0.45 (0.38, 0.53) | -3.63 (-7.29, 0.18) |
| **North Macedonia** | 10.8 (9.07, 12.86) | 0.56 (0.47, 0.66) | 13.18 (10.88, 16.52) | 0.52 (0.43, 0.62) | -7.63 (-13.02, -2.21) |
| **Northern Mariana Islands** | 0.13 (0.1, 0.16) | 0.41 (0.34, 0.48) | 0.21 (0.17, 0.26) | 0.41 (0.34, 0.5) | 0.13 (-3.66, 4.76) |
| **Norway** | 117.16 (110.27, 123.94) | 1.98 (1.85, 2.11) | 174.56 (163.29, 184.98) | 2.11 (1.96, 2.23) | 6.21 (4.8, 7.58) |
| **Oman** | 7.82 (6.34, 9.43) | 0.47 (0.39, 0.57) | 17.83 (14.19, 22.36) | 0.47 (0.39, 0.57) | 0.57 (-2.98, 4.1) |
| **Pakistan** | 426.64 (349.93, 509.97) | 0.45 (0.37, 0.55) | 822.06 (679.13, 992.47) | 0.44 (0.36, 0.54) | -1.61 (-3.8, 0.83) |
| **Palau** | 0.05 (0.04, 0.06) | 0.42 (0.36, 0.5) | 0.09 (0.07, 0.11) | 0.41 (0.34, 0.49) | -3.48 (-6.66, 0.06) |
| **Palestine** | 11.01 (8.77, 13.69) | 0.58 (0.48, 0.7) | 24.17 (19.86, 28.94) | 0.58 (0.48, 0.7) | 0.49 (-2.79, 4.12) |
| **Panama** | 8.89 (7.55, 10.34) | 0.46 (0.39, 0.53) | 19.25 (16.5, 22.13) | 0.47 (0.4, 0.54) | 2.73 (-0.85, 6.43) |
| **Papua New Guinea** | 13.11 (10.85, 15.55) | 0.43 (0.36, 0.51) | 32.7 (26.8, 39.26) | 0.43 (0.36, 0.52) | -0.64 (-3.53, 2.82) |
| **Paraguay** | 19.3 (16, 22.62) | 0.59 (0.49, 0.7) | 38.28 (32.97, 44.1) | 0.63 (0.54, 0.72) | 7.04 (-0.41, 14.58) |
| **Peru** | 70.63 (58.93, 83.34) | 0.41 (0.34, 0.48) | 131.26 (109.49, 156.56) | 0.4 (0.33, 0.47) | -2.3 (-5.57, 1.37) |
| **Philippines** | 190.86 (156.26, 228.79) | 0.39 (0.32, 0.47) | 379.11 (308.74, 462.82) | 0.38 (0.32, 0.47) | -0.54 (-1.36, 0.24) |
| **Poland** | 303.1 (265.5, 342.25) | 0.74 (0.65, 0.84) | 499.97 (450.38, 552.68) | 0.89 (0.8, 0.99) | 20.46 (17.18, 24.19) |
| **Portugal** | 123.01 (110.72, 136.32) | 1.06 (0.97, 1.17) | 251.31 (235.34, 267.72) | 1.43 (1.33, 1.53) | 34.76 (27.79, 42.11) |
| **Puerto Rico** | 21.19 (18.63, 23.97) | 0.59 (0.52, 0.67) | 38.61 (34.69, 42.53) | 0.74 (0.66, 0.82) | 24.22 (19.77, 29.46) |
| **Qatar** | 1.68 (1.34, 2.11) | 0.46 (0.38, 0.55) | 10.77 (8.3, 13.93) | 0.46 (0.38, 0.56) | 1.33 (-2.61, 5.47) |
| **Republic of Korea** | 211.38 (177.38, 252.04) | 0.62 (0.52, 0.74) | 555.53 (448.55, 713.14) | 0.71 (0.58, 0.88) | 14.21 (7.59, 20.99) |
| **Republic of Moldova** | 22.17 (18.41, 26.9) | 0.5 (0.42, 0.6) | 22.34 (18.69, 27.33) | 0.52 (0.44, 0.62) | 3.63 (-0.13, 7.75) |
| **Romania** | 129.06 (107.64, 155.78) | 0.52 (0.44, 0.63) | 128.78 (109.38, 153.83) | 0.52 (0.45, 0.61) | 0.08 (-4.19, 4.33) |
| **Russian Federation** | 822.55 (685.11, 989.63) | 0.53 (0.44, 0.63) | 1080.85 (924.8, 1265.12) | 0.6 (0.52, 0.7) | 14.25 (10.38, 18.05) |
| **Rwanda** | 27.9 (22.75, 33.79) | 0.61 (0.5, 0.76) | 52.05 (42.09, 64.54) | 0.61 (0.5, 0.77) | -0.38 (-3.56, 3.51) |
| **Saint Kitts and Nevis** | 0.25 (0.22, 0.28) | 0.65 (0.57, 0.74) | 0.47 (0.42, 0.54) | 0.74 (0.66, 0.83) | 14.47 (10.5, 18.95) |
| **Saint Lucia** | 0.72 (0.63, 0.82) | 0.65 (0.57, 0.73) | 1.54 (1.37, 1.72) | 0.79 (0.71, 0.88) | 22.84 (17.42, 27.86) |
| **Saint Vincent and the Grenadines** | 0.57 (0.5, 0.65) | 0.64 (0.56, 0.73) | 0.89 (0.79, 1) | 0.72 (0.64, 0.81) | 12.21 (8.08, 16.91) |
| **Samoa** | 0.54 (0.46, 0.63) | 0.47 (0.4, 0.55) | 0.76 (0.63, 0.9) | 0.44 (0.37, 0.52) | -5.42 (-8.87, -1.62) |
| **San Marino** | 0.48 (0.45, 0.51) | 1.63 (1.52, 1.74) | 1.01 (0.96, 1.06) | 1.9 (1.78, 2.01) | 16.66 (13.16, 20.78) |
| **Sao Tome and Principe** | 0.39 (0.32, 0.47) | 0.45 (0.37, 0.55) | 0.65 (0.53, 0.79) | 0.44 (0.37, 0.54) | -1.56 (-5.45, 2.14) |
| **Saudi Arabia** | 65.63 (53.84, 78.79) | 0.48 (0.4, 0.58) | 149.04 (118.96, 186.14) | 0.5 (0.42, 0.59) | 3.12 (-1.26, 7.16) |
| **Senegal** | 28.66 (23.46, 34.2) | 0.54 (0.45, 0.66) | 58.61 (48.82, 71.28) | 0.54 (0.45, 0.66) | 0.57 (-2.21, 3.91) |
| **Serbia** | 55.74 (46.06, 67.48) | 0.54 (0.46, 0.64) | 59.04 (48.74, 72.96) | 0.53 (0.45, 0.64) | -1.41 (-6.35, 3.97) |
| **Seychelles** | 0.22 (0.19, 0.27) | 0.36 (0.3, 0.43) | 0.4 (0.32, 0.49) | 0.36 (0.3, 0.44) | 0.22 (-2.77, 3.16) |
| **Sierra Leone** | 15.03 (12.32, 18.12) | 0.54 (0.45, 0.67) | 31.33 (25.81, 37.96) | 0.55 (0.46, 0.68) | 1.25 (-2.33, 4.79) |
| **Singapore** | 13.35 (11.53, 15.44) | 0.55 (0.48, 0.63) | 36.1 (31.09, 41.59) | 0.51 (0.44, 0.58) | -7.79 (-11.37, -4.14) |
| **Slovakia** | 28.05 (23.53, 34.38) | 0.52 (0.44, 0.63) | 35.48 (29.91, 42.82) | 0.53 (0.45, 0.62) | 1.57 (-2.91, 5.89) |
| **Slovenia** | 13.94 (12.17, 16.04) | 0.69 (0.62, 0.78) | 14.58 (12, 18.25) | 0.51 (0.43, 0.63) | -25.67 (-32.27, -17.69) |
| **Solomon Islands** | 1.13 (0.93, 1.33) | 0.47 (0.39, 0.55) | 2.31 (1.88, 2.76) | 0.46 (0.38, 0.56) | -0.25 (-3.54, 3.57) |
| **Somalia** | 32.21 (26.32, 39.52) | 0.73 (0.6, 0.92) | 90.05 (73.86, 109.66) | 0.76 (0.63, 0.96) | 4.26 (0.39, 8.27) |
| **South Africa** | 152.34 (126.79, 185.24) | 0.53 (0.44, 0.64) | 281.3 (231.11, 351.53) | 0.55 (0.46, 0.68) | 5.22 (3.56, 6.94) |
| **South Sudan** | 21.45 (17.62, 25.89) | 0.57 (0.47, 0.72) | 35.48 (29.06, 43.26) | 0.58 (0.48, 0.73) | 1.49 (-2.63, 5.49) |
| **Spain** | 578.89 (535.37, 623.72) | 1.21 (1.12, 1.31) | 1165.98 (1096.39, 1231.69) | 1.53 (1.43, 1.63) | 25.98 (21.83, 30.62) |
| **Sri Lanka** | 53.44 (44.48, 64.02) | 0.39 (0.32, 0.46) | 90.1 (74.98, 109.35) | 0.38 (0.32, 0.46) | -2.19 (-4.86, 0.77) |
| **Sudan** | 100.4 (79.78, 122.62) | 0.53 (0.44, 0.64) | 181.64 (147.18, 219.57) | 0.52 (0.43, 0.63) | -1.66 (-5.23, 2.6) |
| **Suriname** | 1.78 (1.52, 2.07) | 0.54 (0.46, 0.63) | 3.44 (2.98, 3.94) | 0.59 (0.52, 0.67) | 10.69 (5.89, 15.58) |
| **Sweden** | 264.71 (249.42, 279.1) | 2 (1.86, 2.13) | 407.92 (385.68, 430.02) | 2.27 (2.13, 2.4) | 13.28 (10.65, 16.21) |
| **Switzerland** | 160 (149.95, 170.15) | 1.71 (1.6, 1.83) | 261.77 (246.11, 275.91) | 1.77 (1.65, 1.88) | 3.34 (0.25, 6.36) |
| **Syrian Arab Republic** | 66.41 (54.04, 80.78) | 0.58 (0.49, 0.71) | 78.53 (64.49, 96.32) | 0.58 (0.49, 0.7) | 0.02 (-3.64, 4.19) |
| **Taiwan (Province of China)** | 94.55 (80.65, 110.92) | 0.54 (0.46, 0.63) | 188.14 (162.07, 217.33) | 0.57 (0.5, 0.65) | 6.05 (1.12, 10.39) |
| **Tajikistan** | 22.54 (18.73, 26.78) | 0.49 (0.41, 0.6) | 40.38 (33.31, 48.78) | 0.5 (0.42, 0.61) | 2.58 (-1.31, 6.41) |
| **Thailand** | 186.36 (152.67, 225.95) | 0.42 (0.34, 0.51) | 372.5 (301.86, 472.14) | 0.42 (0.35, 0.52) | 1.52 (-2.71, 5.59) |
| **Timor-Leste** | 2.4 (1.95, 2.91) | 0.42 (0.35, 0.52) | 4.38 (3.63, 5.25) | 0.41 (0.35, 0.5) | -1.78 (-5.17, 2.18) |
| **Togo** | 12.55 (10.21, 15) | 0.53 (0.44, 0.65) | 30.42 (25, 37.21) | 0.54 (0.45, 0.67) | 2.23 (-1.59, 6.06) |
| **Tokelau** | 0.01 (0.01, 0.01) | 0.41 (0.35, 0.5) | 0.01 (0, 0.01) | 0.4 (0.34, 0.48) | -2.95 (-6.52, 1.39) |
| **Tonga** | 0.34 (0.28, 0.4) | 0.46 (0.38, 0.54) | 0.41 (0.34, 0.48) | 0.45 (0.38, 0.54) | -0.67 (-3.94, 2.58) |
| **Trinidad and Tobago** | 5.78 (4.99, 6.58) | 0.58 (0.5, 0.66) | 10.28 (9, 11.72) | 0.63 (0.56, 0.71) | 8.91 (5.33, 13.31) |
| **Tunisia** | 41.7 (34.32, 49.63) | 0.55 (0.46, 0.67) | 64.07 (52.99, 78.2) | 0.54 (0.45, 0.66) | -1.85 (-5.18, 1.92) |
| **Turkey** | 450.43 (404.92, 497.63) | 0.85 (0.75, 0.95) | 737.15 (640.33, 839.55) | 0.92 (0.81, 1.03) | 7.91 (3.85, 12) |
| **Turkmenistan** | 14.57 (12.07, 17.13) | 0.46 (0.39, 0.55) | 21.79 (18.05, 26.28) | 0.46 (0.38, 0.54) | -1.92 (-5.64, 1.73) |
| **Tuvalu** | 0.04 (0.03, 0.04) | 0.43 (0.36, 0.51) | 0.05 (0.04, 0.06) | 0.42 (0.35, 0.51) | -1.73 (-5.24, 1.91) |
| **Uganda** | 68.54 (55.43, 83.29) | 0.64 (0.52, 0.8) | 149.98 (121.59, 181.49) | 0.62 (0.51, 0.77) | -3.14 (-6.43, 0.5) |
| **Ukraine** | 289.65 (242.83, 351.56) | 0.51 (0.43, 0.61) | 275.06 (231.56, 332.03) | 0.52 (0.44, 0.62) | 1.9 (-1.67, 5.47) |
| **United Arab Emirates** | 6.84 (5.44, 8.48) | 0.46 (0.38, 0.55) | 42.14 (30.96, 55.67) | 0.49 (0.42, 0.59) | 8.46 (4.15, 12.81) |
| **United Kingdom** | 1590.5 (1504.24, 1673.09) | 1.99 (1.88, 2.1) | 2754.41 (2596.62, 2902.89) | 2.47 (2.32, 2.61) | 24.3 (22.38, 26.42) |
| **United Republic of Tanzania** | 107.15 (87.48, 130.07) | 0.64 (0.52, 0.81) | 235.41 (191.12, 288.6) | 0.63 (0.51, 0.79) | -1.71 (-4.85, 1.47) |
| **United States of America** | 5074.89 (4788.52, 5377.6) | 1.72 (1.62, 1.83) | 9956.12 (9522.38, 10425.38) | 1.94 (1.85, 2.03) | 12.49 (9.17, 16.17) |
| **United States Virgin Islands** | 0.87 (0.78, 0.96) | 0.93 (0.84, 1.01) | 2.08 (1.93, 2.22) | 1.24 (1.15, 1.33) | 34 (29.25, 38.99) |
| **Uruguay** | 41.14 (37.99, 44.4) | 1.12 (1.03, 1.22) | 57.91 (53.89, 62.04) | 1.24 (1.15, 1.34) | 10.7 (7.3, 14.12) |
| **Uzbekistan** | 90.46 (75.88, 106.84) | 0.51 (0.43, 0.62) | 147.95 (120.63, 180.81) | 0.5 (0.42, 0.61) | -1.9 (-4.9, 1.73) |
| **Vanuatu** | 0.55 (0.45, 0.64) | 0.51 (0.43, 0.59) | 1.15 (0.96, 1.35) | 0.5 (0.42, 0.59) | -1.2 (-4.59, 2.15) |
| **Venezuela (Bolivarian Republic of)** | 68.41 (57.06, 79.96) | 0.47 (0.4, 0.55) | 150.4 (129.69, 173.68) | 0.52 (0.45, 0.6) | 10.33 (6.87, 14.42) |
| **Viet Nam** | 231.11 (192.3, 275.34) | 0.44 (0.37, 0.54) | 411.77 (334.67, 507.63) | 0.43 (0.35, 0.51) | -3.31 (-6.49, 0.08) |
| **Yemen** | 67.95 (53.5, 85.7) | 0.53 (0.44, 0.65) | 143.58 (116.06, 172.65) | 0.54 (0.45, 0.66) | 1.03 (-2.66, 5.26) |
| **Zambia** | 34.02 (27.85, 41.04) | 0.7 (0.57, 0.87) | 78.88 (64.41, 96.35) | 0.7 (0.57, 0.88) | 0.61 (-2.64, 4.46) |
| **Zimbabwe** | 38.12 (31.23, 45.38) | 0.54 (0.45, 0.65) | 59.62 (49.16, 71.79) | 0.55 (0.46, 0.66) | 0.86 (-2.85, 4.08) |
| **DALYs** | | | | | |
| **Afghanistan** | 256.92 (182.74, 393.16) | 2.77 (1.88, 4.35) | 628.03 (483.69, 826.81) | 2.58 (1.84, 3.62) | -6.94 (-34.52, 34.05) |
| **Albania** | 143.11 (127.82, 159.86) | 5.15 (4.64, 5.69) | 198.48 (149.11, 257.69) | 6.04 (4.62, 7.67) | 17.35 (-10.38, 50.84) |
| **Algeria** | 473.82 (370.76, 616.55) | 2.59 (1.94, 3.5) | 1023.08 (805.57, 1284.6) | 2.54 (1.99, 3.21) | -2.03 (-28.3, 36.31) |
| **American Samoa** | 2.51 (1.92, 3.22) | 8.73 (6.56, 11.27) | 1.62 (1.25, 2.04) | 2.96 (2.28, 3.69) | -66.15 (-76.49, -50.85) |
| **Andorra** | 26.93 (16.96, 41.03) | 51.06 (32.52, 77.03) | 69.28 (44.22, 104.63) | 54.46 (35.07, 81.63) | 6.64 (-31.56, 67.33) |
| **Angola** | 94.33 (70.16, 128.18) | 1.09 (0.78, 1.51) | 282.53 (220.9, 347.84) | 1.12 (0.86, 1.41) | 3.41 (-23.64, 36.27) |
| **Antigua and Barbuda** | 2.55 (2.32, 2.8) | 4.94 (4.5, 5.43) | 11.99 (9.72, 14.53) | 12.07 (9.8, 14.76) | 144.14 (92.71, 205.63) |
| **Argentina** | 2002.42 (1862.12, 2152.05) | 6.1 (5.69, 6.56) | 10274.17 (9277.76, 11050) | 20.51 (18.53, 22.15) | 236.11 (204.47, 268.91) |
| **Armenia** | 86.1 (72.85, 107.49) | 2.73 (2.32, 3.41) | 76.9 (64.25, 90.34) | 2.04 (1.71, 2.41) | -25.41 (-42.77, -10) |
| **Australia** | 8333.33 (7910.71, 8732) | 46.17 (43.58, 48.7) | 19496.41 (17556.73, 21406.66) | 55.54 (49.92, 61.27) | 20.3 (6.69, 34.12) |
| **Austria** | 2248.53 (2108.13, 2392.43) | 26.15 (24, 28.54) | 4550.22 (4178.06, 4911.51) | 34.61 (31.59, 37.72) | 32.38 (17.65, 49.39) |
| **Azerbaijan** | 123.74 (101.93, 152.11) | 1.89 (1.54, 2.33) | 252.04 (201.24, 317.06) | 2.23 (1.82, 2.74) | 17.87 (-6.85, 50.59) |
| **Bahamas** | 14.95 (13.35, 16.87) | 7.53 (6.73, 8.45) | 66.39 (52, 84.41) | 16.37 (12.82, 20.77) | 117.42 (65.95, 185.45) |
| **Bahrain** | 15.22 (12.97, 17.84) | 4.5 (3.81, 5.3) | 32.64 (26.77, 39.9) | 2.07 (1.72, 2.5) | -54.11 (-64.12, -42.57) |
| **Bangladesh** | 2603.17 (1814.66, 3771.38) | 3.1 (2.07, 4.48) | 5238.86 (3331.87, 7953.66) | 3.6 (2.26, 5.51) | 15.96 (-27, 69.34) |
| **Barbados** | 15.47 (14.35, 16.71) | 6.46 (5.99, 7.03) | 138.2 (105.1, 175.24) | 33.01 (24.85, 42.17) | 411.19 (284.75, 547.36) |
| **Belarus** | 353.3 (288.86, 480.34) | 3.07 (2.54, 4.07) | 844.35 (640, 1124.18) | 6.19 (4.73, 8.19) | 101.45 (48.12, 168.64) |
| **Belgium** | 7269.76 (6909.67, 7631.2) | 55.52 (52.52, 58.62) | 7424.66 (6690.43, 8194.91) | 40.43 (36.58, 44.61) | -27.18 (-34.83, -19.14) |
| **Belize** | 7.34 (6.42, 8.36) | 5.42 (4.86, 6.02) | 32.73 (27.09, 38.43) | 9.49 (7.86, 11.19) | 75.14 (40.85, 111.5) |
| **Benin** | 82.92 (62.16, 114.3) | 2.58 (1.87, 3.53) | 151.25 (111.96, 196.25) | 1.67 (1.22, 2.18) | -35.35 (-56.72, -3.74) |
| **Bermuda** | 5.23 (4.47, 6.42) | 8.5 (7.21, 10.52) | 17.84 (14.25, 22.05) | 17.37 (13.9, 21.33) | 104.46 (53.12, 167.55) |
| **Bhutan** | 11.37 (6.44, 18.09) | 2.45 (1.4, 4.07) | 22.36 (12.78, 36.59) | 3.41 (1.92, 5.72) | 39.33 (-10, 117.23) |
| **Bolivia (Plurinational State of)** | 390.73 (293.97, 522.48) | 8.6 (6.57, 11.59) | 1110.13 (766.66, 1599.8) | 11.3 (7.79, 16.3) | 31.47 (-12.36, 91.63) |
| **Bosnia and Herzegovina** | 655.17 (552.54, 769.58) | 14.32 (12.11, 16.87) | 79.96 (63.38, 101.3) | 1.91 (1.54, 2.36) | -86.63 (-89.83, -82.84) |
| **Botswana** | 22.34 (15.57, 31.12) | 2.61 (1.69, 3.85) | 49.96 (35.17, 69.09) | 2.59 (1.79, 3.61) | -0.85 (-38.18, 56.66) |
| **Brazil** | 14226.08 (13344.38, 15302.53) | 11.7 (11.11, 12.42) | 43968.36 (40197.57, 46922.63) | 18.74 (17.14, 20.03) | 60.16 (39.93, 75.79) |
| **Brunei Darussalam** | 6.76 (4.79, 11.21) | 3.11 (2.32, 4.71) | 23.79 (17.9, 33.67) | 6.39 (4.69, 9.39) | 105.38 (19.22, 245.14) |
| **Bulgaria** | 914.42 (850.2, 987.58) | 9.44 (8.74, 10.27) | 332.54 (263.96, 411.74) | 3.53 (2.84, 4.32) | -62.64 (-70.33, -52.99) |
| **Burkina Faso** | 155.93 (109.49, 223.37) | 2.33 (1.52, 3.5) | 276.3 (210.28, 355.68) | 1.61 (1.16, 2.19) | -30.95 (-50.64, -2.34) |
| **Burundi** | 46.69 (35.73, 62.08) | 0.93 (0.7, 1.35) | 95.74 (72.17, 126.59) | 0.86 (0.62, 1.17) | -8.25 (-28.55, 18.61) |
| **Cabo Verde** | 3.89 (3.26, 4.59) | 1.36 (1.12, 1.66) | 10.61 (8.57, 13.12) | 2.11 (1.71, 2.63) | 55.08 (17.29, 102.57) |
| **Cambodia** | 208.99 (161.36, 276.65) | 2.76 (2.07, 3.61) | 394.02 (282.61, 527.66) | 2.69 (1.91, 3.63) | -2.43 (-29.76, 34.37) |
| **Cameroon** | 235.85 (166.25, 316.8) | 3.61 (2.46, 5.02) | 500 (342.9, 748.93) | 2.65 (1.72, 4.22) | -26.8 (-56.6, 17.83) |
| **Canada** | 12006.23 (11441.59, 12560.14) | 40.24 (38.19, 42.3) | 27696.61 (25426.7, 30052.54) | 47 (43.37, 50.81) | 16.8 (7.08, 26.75) |
| **Central African Republic** | 23.56 (17.66, 33.04) | 1.02 (0.74, 1.51) | 37.8 (29.31, 49.04) | 0.84 (0.62, 1.13) | -17.72 (-39.27, 7.55) |
| **Chad** | 92.12 (64.49, 134.72) | 2.21 (1.44, 3.56) | 174.67 (135.57, 220.54) | 1.55 (1.15, 2.07) | -29.89 (-52.17, 0.29) |
| **Chile** | 1673.87 (1551.04, 1820.82) | 14.43 (13.43, 15.59) | 5559.76 (4987.63, 6130.87) | 24.94 (22.35, 27.65) | 72.81 (50.99, 94.21) |
| **China** | 147940.08 (130847.44, 165917.21) | 13.13 (11.63, 14.69) | 106779.05 (92230.18, 121988.77) | 6.7 (5.88, 7.59) | -48.97 (-57.43, -38.86) |
| **Colombia** | 1876.79 (1739.48, 2033.75) | 7.48 (7.02, 7.98) | 7278.11 (5472.74, 9428.55) | 14.34 (10.78, 18.55) | 91.75 (42.62, 149.88) |
| **Comoros** | 4.2 (2.3, 5.73) | 1.01 (0.58, 1.44) | 7.17 (5.25, 9.15) | 1.07 (0.78, 1.38) | 5.32 (-23.17, 77.14) |
| **Congo** | 24.09 (18.44, 31.96) | 1.24 (0.94, 1.68) | 51.08 (38.15, 67.6) | 1.18 (0.86, 1.58) | -5.29 (-35.64, 32.66) |
| **Cook Islands** | 1.32 (0.99, 1.66) | 8.98 (6.72, 11.33) | 1.02 (0.74, 1.38) | 4.24 (3.12, 5.66) | -52.85 (-66.69, -32.53) |
| **Costa Rica** | 136.42 (125.09, 150.46) | 6.04 (5.58, 6.53) | 1470.17 (1108.57, 1889) | 28.22 (21.23, 36.4) | 367.3 (247.61, 513.46) |
| **Croatia** | 216.79 (154.26, 327.11) | 19.45 (17.7, 21.3) | 328.54 (240.77, 430.16) | 20.81 (15.93, 26.51) | 6.98 (-20.16, 38.56) |
| **Cuba** | 1011.97 (929.42, 1102.37) | 6.43 (5.97, 6.91) | 1220.28 (935.73, 1557.29) | 20.11 (16.28, 24.54) | 212.67 (148.48, 287.23) |
| **Cyprus** | 654.91 (607.09, 703.46) | 16.63 (13.62, 20.76) | 3201.88 (2567.87, 3946.32) | 21.34 (17.72, 25.56) | 28.33 (-5.33, 76.23) |
| **Czechia** | 138.8 (112.83, 173.3) | 18.05 (16.47, 20) | 392.01 (325.52, 471.02) | 16.54 (13.39, 20.3) | -8.41 (-26.77, 14.16) |
| **Côte d'Ivoire** | 1951.53 (1816.41, 2103.82) | 2.97 (2.02, 4.52) | 2531.1 (2060.92, 3075.95) | 1.77 (1.27, 2.39) | -40.17 (-62.07, -7.82) |
| **Democratic People's Republic of Korea** | 2377.25 (1409.15, 4167.92) | 9.34 (6.02, 14.65) | 1706.82 (1144.71, 2584.46) | 5.81 (3.9, 8.54) | -37.82 (-64.73, 2.69) |
| **Democratic Republic of the Congo** | 346.81 (256.5, 505.63) | 1.02 (0.76, 1.57) | 662.79 (520.17, 865.63) | 0.92 (0.7, 1.26) | -9.62 (-33.48, 17.11) |
| **Denmark** | 2494.41 (2315.23, 2688.58) | 34.4 (32.06, 36.9) | 3733.11 (3373.62, 4137.73) | 36.13 (32.83, 39.82) | 5.05 (-7.84, 18.86) |
| **Djibouti** | 4.33 (3.19, 5.97) | 1.06 (0.73, 1.5) | 12.83 (9.1, 17.09) | 1.16 (0.81, 1.61) | 9.55 (-18.64, 47.93) |
| **Dominica** | 4.65 (3.97, 5.42) | 7.18 (6.1, 8.41) | 19.49 (14.22, 25.5) | 25.97 (18.71, 34.38) | 261.46 (147.29, 410.82) |
| **Dominican Republic** | 425.2 (337.58, 531.44) | 7.18 (5.91, 8.54) | 1314.85 (819.11, 2058.83) | 12.95 (8.07, 20.1) | 80.38 (10.6, 187.33) |
| **Ecuador** | 382.68 (347.4, 420.58) | 5.31 (4.85, 5.8) | 1864.06 (1420.73, 2431.31) | 11.63 (8.87, 15.18) | 119.24 (64.94, 191.44) |
| **Egypt** | 2017.32 (1689.81, 2437.63) | 4.68 (3.95, 5.49) | 6482.73 (3806.9, 10065.94) | 7.94 (4.57, 12.38) | 69.44 (-4.41, 176.85) |
| **El Salvador** | 183.37 (162.75, 204.88) | 4.57 (4.09, 5.08) | 227.48 (167.84, 294.85) | 3.87 (2.85, 5.04) | -15.22 (-38.69, 14.07) |
| **Equatorial Guinea** | 3.5 (2.58, 5.15) | 0.93 (0.67, 1.39) | 11.88 (8.11, 16.6) | 1.2 (0.8, 1.75) | 29.07 (-19.47, 104.72) |
| **Eritrea** | 24.06 (18.4, 31.92) | 0.97 (0.71, 1.42) | 65.17 (49.75, 84.12) | 1.11 (0.82, 1.47) | 13.84 (-12.15, 48.93) |
| **Estonia** | 100.31 (89.39, 119.4) | 5.33 (4.76, 6.29) | 66.42 (53.46, 82.64) | 3.51 (2.83, 4.33) | -34.21 (-48.32, -18.68) |
| **Eswatini** | 13.25 (9.98, 17.68) | 2.68 (1.88, 3.77) | 21.5 (15.63, 29.44) | 2.54 (1.79, 3.55) | -5.32 (-40.77, 60.15) |
| **Ethiopia** | 488.16 (379.09, 682.14) | 1.07 (0.77, 1.65) | 899.58 (730.79, 1097.73) | 0.94 (0.71, 1.24) | -12.64 (-32.53, 9.15) |
| **Fiji** | 15.86 (12.37, 19.94) | 2.98 (2.25, 3.85) | 26.89 (19.63, 35.62) | 3 (2.19, 3.97) | 0.61 (-34.13, 51.51) |
| **Finland** | 3212.36 (3029.28, 3408.49) | 49.43 (46.49, 52.67) | 5339.29 (4645.26, 6043.77) | 53.03 (46.88, 59.36) | 7.3 (-6.37, 23.47) |
| **France** | 26612.4 (25417.49, 27903.48) | 39.66 (37.39, 42.06) | 43385.42 (38660.81, 48067.02) | 40.39 (36.07, 44.67) | 1.84 (-10.3, 14.59) |
| **Gabon** | 11.36 (8.31, 17.85) | 1.33 (0.95, 2.08) | 19.41 (14.28, 26.1) | 1.29 (0.94, 1.77) | -3.1 (-38.33, 36.35) |
| **Gambia** | 14.3 (9.68, 20.59) | 2.3 (1.49, 3.49) | 29.35 (22.59, 36.32) | 1.86 (1.39, 2.4) | -18.76 (-48.92, 28.61) |
| **Georgia** | 192.86 (153.93, 288.98) | 3.14 (2.51, 4.64) | 198.04 (166.11, 235.53) | 4.28 (3.59, 5.15) | 36.29 (-6.49, 79.81) |
| **Germany** | 35514.76 (34069.61, 37024.09) | 36.15 (34.3, 38.38) | 50814.88 (47073.69, 54563.52) | 34.58 (32.25, 36.99) | -4.33 (-12.86, 3.88) |
| **Ghana** | 252.52 (194.32, 322.14) | 2.62 (1.98, 3.43) | 676.78 (506.14, 895.71) | 2.98 (2.2, 4.03) | 13.8 (-26.14, 74.67) |
| **Greece** | 1158.22 (1058.93, 1300.71) | 10.27 (9.02, 12.88) | 4868.5 (4272.19, 5503.05) | 33.58 (28.75, 39.47) | 227.13 (172.15, 288.78) |
| **Greenland** | 5.64 (4.61, 6.97) | 12.64 (10.48, 15.32) | 8.42 (6.68, 10.3) | 12.09 (9.66, 14.98) | -4.35 (-27.6, 27.28) |
| **Grenada** | 4.04 (3.57, 4.54) | 5.89 (5.25, 6.57) | 20.96 (17.89, 23.87) | 18.42 (15.85, 20.96) | 212.89 (158.82, 266.81) |
| **Guam** | 22.01 (17.3, 27.45) | 24.22 (19, 30.11) | 4.8 (3.78, 6.08) | 2.48 (1.98, 3.11) | -89.75 (-92.58, -85.68) |
| **Guatemala** | 311.25 (273.52, 353.63) | 5.24 (4.61, 5.92) | 566.11 (444.37, 716.69) | 4.48 (3.49, 5.72) | -14.57 (-34.17, 12.58) |
| **Guinea** | 119.63 (84.09, 168.68) | 2.55 (1.71, 3.7) | 162.31 (124.13, 210.12) | 1.77 (1.31, 2.36) | -30.63 (-52.93, 3.13) |
| **Guinea-Bissau** | 20.67 (15.15, 29.01) | 3.35 (2.34, 4.76) | 25.34 (19.66, 32.47) | 2.04 (1.52, 2.72) | -39.12 (-59.99, -7.8) |
| **Guyana** | 29.29 (24.99, 33.67) | 5.35 (4.59, 6.09) | 109.52 (82.76, 142.15) | 15.09 (11.42, 19.5) | 181.96 (108.1, 275.57) |
| **Haiti** | 699.99 (394.99, 1230.24) | 10.39 (7.32, 15.35) | 1385.96 (893.92, 2206.94) | 13 (8.6, 18.81) | 25.12 (-24.85, 100.93) |
| **Honduras** | 500.79 (366.86, 700.75) | 12.13 (9.44, 15.43) | 1357.02 (840.32, 1989.78) | 18.27 (11.09, 26.97) | 50.61 (-8.39, 130.14) |
| **Hungary** | 2466.89 (2302.16, 2648.15) | 23.78 (21.55, 26.23) | 2746.99 (2222.97, 3379.72) | 21.22 (17.2, 26.12) | -10.74 (-30.08, 10.79) |
| **Iceland** | 95.05 (87.95, 102.2) | 35.89 (33.25, 38.68) | 178.91 (156.11, 203.2) | 37.97 (32.9, 43.56) | 5.78 (-9.36, 23.18) |
| **India** | 21308.78 (16526.05, 26559.45) | 3.09 (2.33, 4) | 57933.02 (46259.85, 70688.82) | 4.53 (3.6, 5.54) | 46.31 (15.93, 81.04) |
| **Indonesia** | 4587.42 (3623.52, 5998.5) | 3.09 (2.41, 3.97) | 8799.34 (6191.88, 12072.9) | 3.4 (2.41, 4.64) | 10 (-13.47, 36.48) |
| **Iran (Islamic Republic of)** | 1418.8 (1120.36, 1935.73) | 3.27 (2.59, 4.41) | 3832.14 (3358.66, 4363.1) | 4.7 (4.12, 5.36) | 43.7 (1.01, 90.31) |
| **Iraq** | 222.22 (177.53, 284.85) | 1.66 (1.27, 2.23) | 639.93 (494.79, 796.23) | 1.84 (1.41, 2.32) | 10.58 (-26.99, 58.44) |
| **Ireland** | 1610.88 (1511.98, 1718.6) | 42.92 (40.17, 46.05) | 3938.48 (3496.64, 4340.21) | 58.75 (52.03, 64.85) | 36.87 (19.2, 54.17) |
| **Israel** | 1426.72 (1288.51, 1598.78) | 29.41 (26.62, 32.84) | 2836.77 (2572.29, 3101.59) | 27.27 (24.75, 29.86) | -7.26 (-19.82, 6.87) |
| **Italy** | 20880.75 (20279.16, 21583.4) | 33.07 (31.49, 35.41) | 38876.33 (34851.99, 42306.26) | 37.56 (33.92, 40.9) | 13.58 (2.1, 25.69) |
| **Jamaica** | 110.52 (100.66, 121.9) | 5.81 (5.35, 6.3) | 383.16 (284.78, 495.85) | 13.89 (10.14, 18.22) | 139.04 (77.04, 214.18) |
| **Japan** | 27465.91 (26608.08, 28235.75) | 17.19 (16.65, 17.68) | 49797.5 (44610.02, 53954.08) | 18.51 (16.97, 19.8) | 7.7 (-0.01, 14.54) |
| **Jordan** | 64.56 (53.05, 80.4) | 2.37 (1.91, 3.01) | 338.32 (264.9, 446.35) | 3.22 (2.52, 4.19) | 35.75 (-4.53, 97.02) |
| **Kazakhstan** | 160.53 (125.08, 200.14) | 1.05 (0.83, 1.29) | 263.02 (219.07, 311.5) | 1.36 (1.13, 1.62) | 30.22 (11.77, 51.51) |
| **Kenya** | 184.85 (150.42, 222.63) | 0.93 (0.73, 1.23) | 448.81 (367.72, 552.66) | 1.07 (0.86, 1.34) | 14.35 (-8.77, 41.45) |
| **Kiribati** | 7.04 (5.35, 9.1) | 15.49 (11.65, 20.21) | 12.33 (8.63, 17.87) | 15.05 (10.41, 21.99) | -2.83 (-37.87, 57.28) |
| **Kuwait** | 243.9 (192.82, 317.71) | 15.49 (12.56, 19.7) | 209.74 (154.56, 262.36) | 5.93 (4.29, 7.51) | -61.73 (-72.5, -48.22) |
| **Kyrgyzstan** | 92.23 (74.53, 132.45) | 2.55 (2.05, 3.74) | 206.28 (179.68, 234.56) | 3.34 (2.91, 3.8) | 30.86 (-12.35, 70.04) |
| **Lao People's Democratic Republic** | 102.32 (75.63, 149.65) | 3.06 (2.24, 4.35) | 162.07 (109.89, 225.71) | 2.72 (1.84, 3.82) | -11.03 (-40.13, 29.23) |
| **Latvia** | 81.23 (70.74, 101.13) | 2.57 (2.21, 3.13) | 251.45 (207.56, 303.97) | 8.3 (6.93, 10.03) | 223.48 (150.98, 307.19) |
| **Lebanon** | 87.93 (65.19, 130.53) | 3.14 (2.3, 4.74) | 169.36 (112.27, 260.82) | 3.28 (2.16, 5.08) | 4.49 (-32.14, 55.64) |
| **Lesotho** | 28.44 (19.91, 41.5) | 2.12 (1.41, 3.25) | 41.28 (29.76, 56.91) | 2.48 (1.75, 3.48) | 16.67 (-27.9, 90.49) |
| **Liberia** | 41.47 (30.64, 54.87) | 2.75 (1.97, 3.68) | 56.82 (41.59, 77.59) | 1.65 (1.16, 2.38) | -40.05 (-61.7, -7.67) |
| **Libya** | 77.12 (59.86, 104.36) | 2.66 (1.96, 3.74) | 243.39 (148.8, 362.81) | 3.71 (2.3, 5.43) | 39.49 (-25.61, 142.03) |
| **Lithuania** | 287.89 (251.71, 358.08) | 7.03 (6.16, 8.7) | 935.68 (760.32, 1143.87) | 21.52 (17.79, 25.97) | 205.94 (132.43, 295.36) |
| **Luxembourg** | 159.6 (148.03, 171.23) | 34.75 (31.97, 37.63) | 249.53 (214.99, 287.96) | 29.29 (24.87, 34.39) | -15.7 (-29.24, 0.51) |
| **Madagascar** | 126.9 (100.09, 167.68) | 1.17 (0.88, 1.55) | 235.16 (189.58, 282.77) | 1.03 (0.81, 1.28) | -12.11 (-31.09, 8.94) |
| **Malawi** | 90 (68.06, 121.48) | 1.02 (0.75, 1.4) | 167.8 (131.77, 211.56) | 1.02 (0.77, 1.31) | 0.06 (-22.25, 29.39) |
| **Malaysia** | 266.48 (222.08, 320.06) | 2.02 (1.64, 2.46) | 560.39 (436.75, 721.78) | 1.82 (1.42, 2.35) | -9.74 (-34.84, 23.64) |
| **Maldives** | 9.53 (5.2, 18.42) | 4.83 (3.22, 7.82) | 24.64 (17.89, 34.94) | 6.06 (4.31, 8.7) | 25.47 (-32.72, 125.48) |
| **Mali** | 164.91 (117.46, 232.19) | 2.6 (1.73, 3.87) | 261.37 (194.55, 341.32) | 1.64 (1.16, 2.25) | -36.82 (-55.48, -11.84) |
| **Malta** | 114.77 (105.92, 124.11) | 28.88 (26.44, 31.55) | 240.99 (206.02, 277.45) | 35.28 (29.52, 41.91) | 22.18 (0.81, 48.14) |
| **Marshall Islands** | 1.53 (1.15, 2.04) | 7.34 (5.29, 9.95) | 2.14 (1.56, 2.89) | 4.69 (3.42, 6.36) | -36.09 (-58.49, -1.48) |
| **Mauritania** | 48.73 (38.21, 61.74) | 3.67 (2.79, 4.82) | 57.79 (40.7, 78.4) | 1.95 (1.35, 2.71) | -46.81 (-64.8, -22.38) |
| **Mauritius** | 60.67 (55.8, 66.18) | 6.57 (6.07, 7.1) | 128.9 (102.35, 160.62) | 8.13 (6.38, 10.18) | 23.75 (-2.07, 55.39) |
| **Mexico** | 5289.14 (4953.8, 5647.52) | 7.99 (7.66, 8.37) | 16337.12 (13686.49, 18901.04) | 13.16 (11.06, 15.23) | 64.59 (36.37, 91.74) |
| **Micronesia (Federated States of)** | 4.2 (3.04, 5.63) | 7.28 (5.05, 9.96) | 4.36 (2.92, 6.1) | 4.75 (3.18, 6.64) | -34.7 (-60.39, 5.89) |
| **Monaco** | 4.74 (3.69, 6.07) | 10.92 (8.45, 14.42) | 7.81 (5.97, 10.12) | 11.11 (8.6, 14.39) | 1.81 (-26.33, 39.61) |
| **Mongolia** | 145.04 (95.72, 234.91) | 7.01 (5.28, 10.13) | 217.31 (155.52, 313.35) | 6.86 (5.01, 9.6) | -2.1 (-40.08, 52.28) |
| **Montenegro** | 15.6 (13.16, 18.35) | 2.43 (2.05, 2.85) | 19.64 (16.21, 23.64) | 2.47 (2.04, 2.96) | 1.74 (-16.47, 24.39) |
| **Morocco** | 391.79 (312.19, 492.48) | 2.01 (1.57, 2.63) | 902.97 (687.31, 1184.69) | 2.48 (1.9, 3.23) | 23.82 (-11.83, 67.99) |
| **Mozambique** | 116.93 (88.58, 162.16) | 0.96 (0.69, 1.41) | 295.47 (227.44, 376.32) | 1.09 (0.82, 1.47) | 13.54 (-13.38, 47.9) |
| **Myanmar** | 1096.69 (799.22, 1540.94) | 3.23 (2.36, 4.47) | 1614.48 (1141.04, 2218.68) | 3.02 (2.13, 4.12) | -6.67 (-37.82, 39.5) |
| **Namibia** | 24.47 (17.88, 32.08) | 2.56 (1.83, 3.45) | 42.91 (32.12, 57.19) | 2.26 (1.7, 3.05) | -11.65 (-40.75, 38.01) |
| **Nauru** | 0.49 (0.34, 0.76) | 8.99 (6.08, 14.18) | 0.34 (0.24, 0.48) | 5.13 (3.61, 7.12) | -42.86 (-63.66, -15.77) |
| **Nepal** | 400.08 (270.06, 600.4) | 2.49 (1.56, 4.03) | 904.44 (582.77, 1407.88) | 3.43 (2.17, 5.46) | 37.81 (-6.1, 94.86) |
| **Netherlands** | 7820.66 (7406.04, 8250.72) | 46.31 (43.38, 49.65) | 13710.09 (12481.07, 14951.32) | 48.58 (43.94, 53.14) | 4.9 (-6.05, 16.53) |
| **New Zealand** | 1740.16 (1643.72, 1839.81) | 46.67 (44.02, 49.28) | 3617.38 (3314.9, 3917.28) | 53.25 (49.11, 57.39) | 14.09 (4.15, 25.97) |
| **Nicaragua** | 96.69 (82.45, 114.63) | 3.9 (3.24, 4.69) | 201.38 (156.01, 258.82) | 3.84 (2.96, 4.95) | -1.58 (-29.35, 33.09) |
| **Niger** | 121.58 (82.93, 182.88) | 2.32 (1.41, 3.75) | 226.09 (165.01, 300.5) | 1.34 (0.9, 1.98) | -42.27 (-57.83, -17.13) |
| **Nigeria** | 1560.91 (1172.8, 2019.51) | 2.42 (1.77, 3.18) | 2783.49 (2098.03, 3670.54) | 1.88 (1.38, 2.54) | -21.98 (-49.1, 15.94) |
| **Niue** | 0.2 (0.14, 0.27) | 9.43 (6.61, 13.28) | 0.12 (0.09, 0.17) | 5.74 (4.27, 7.68) | -39.11 (-60.83, -1.99) |
| **North Macedonia** | 250.22 (194.25, 340.3) | 13.29 (10.03, 18.69) | 74.83 (58.44, 93.02) | 2.72 (2.14, 3.34) | -79.57 (-86.23, -71.08) |
| **Northern Mariana Islands** | 1.38 (1.06, 1.82) | 4.63 (3.58, 6.06) | 1.38 (1.06, 1.76) | 2.23 (1.76, 2.78) | -51.84 (-66.42, -34.1) |
| **Norway** | 2417.16 (2336.25, 2495.65) | 43.51 (41.98, 44.97) | 3329.73 (3047.9, 3611.33) | 40.05 (36.76, 43.16) | -7.94 (-16.35, 0.57) |
| **Oman** | 32.47 (24.54, 54.31) | 2.68 (1.88, 4.96) | 100.3 (68.03, 130.33) | 3.15 (1.89, 4.36) | 17.5 (-32.77, 84.63) |
| **Pakistan** | 2690.19 (1874.37, 3868.49) | 3.02 (2.03, 4.32) | 8525.49 (6712.35, 11077.38) | 4.94 (3.86, 6.39) | 63.74 (16.83, 135.81) |
| **Palau** | 0.2 (0.15, 0.25) | 1.56 (1.18, 2.05) | 0.4 (0.29, 0.53) | 1.66 (1.28, 2.14) | 6.01 (-26.49, 53.18) |
| **Palestine** | 26.6 (20.49, 35.42) | 1.57 (1.18, 2.16) | 84.29 (69.47, 102.23) | 2.07 (1.7, 2.47) | 31.66 (-8.32, 85.92) |
| **Panama** | 123.27 (111.74, 135.93) | 6.56 (5.99, 7.15) | 361.98 (273.78, 467.03) | 8.71 (6.59, 11.25) | 32.73 (-0.2, 71.8) |
| **Papua New Guinea** | 88.05 (50.58, 136.34) | 3.28 (1.72, 5.36) | 155.48 (97.59, 236.5) | 2.17 (1.25, 3.49) | -33.83 (-53.62, -4.41) |
| **Paraguay** | 144.68 (122.11, 171.98) | 4.74 (3.99, 5.6) | 782.88 (563.02, 1088.87) | 12.84 (9.23, 17.81) | 170.69 (82.33, 304.42) |
| **Peru** | 851.31 (704.58, 1020.72) | 5.25 (4.32, 6.32) | 1749.55 (1226.12, 2428.04) | 5.31 (3.73, 7.35) | 1.1 (-33.24, 49.07) |
| **Philippines** | 1229.49 (1067.21, 1388.37) | 2.48 (2.09, 2.84) | 2787.67 (2363.82, 3257.46) | 2.78 (2.35, 3.26) | 11.94 (-11.47, 40.81) |
| **Poland** | 4533.61 (4307.9, 4837.89) | 12.53 (11.79, 13.59) | 12003.07 (10110.62, 14081.55) | 22.96 (19.39, 26.84) | 83.28 (54.22, 117.01) |
| **Portugal** | 2288.75 (2133.78, 2475.31) | 22.66 (20.54, 25.5) | 6057.82 (5419.16, 6684.12) | 36.2 (31.96, 40.56) | 59.75 (34.3, 87.19) |
| **Puerto Rico** | 245.56 (227.66, 264.73) | 6.97 (6.45, 7.53) | 1031 (782.33, 1336.82) | 19.87 (15.11, 25.99) | 185.3 (115.91, 271.85) |
| **Qatar** | 6.92 (5.5, 8.68) | 2.24 (1.72, 2.97) | 49.83 (38.78, 63.54) | 2.13 (1.65, 2.73) | -4.75 (-34.32, 34.57) |
| **Republic of Korea** | 8291.93 (6729, 10276.89) | 23.89 (19.1, 29.91) | 2842.12 (2481.9, 3229.91) | 3.53 (3.08, 3.97) | -85.22 (-88.63, -81.2) |
| **Republic of Moldova** | 409.96 (364.84, 475.1) | 9.27 (8.23, 10.78) | 264.02 (227.41, 302.5) | 6.07 (5.28, 6.91) | -34.5 (-46.45, -20.86) |
| **Romania** | 3843.33 (3434.14, 4352.82) | 19.27 (16.71, 22.4) | 2085.61 (1718.19, 2507.03) | 9.38 (7.87, 11.17) | -51.34 (-61.33, -39.48) |
| **Russian Federation** | 7611.71 (6645.03, 10006.71) | 4.71 (4.13, 6.11) | 22677.76 (19611.08, 25859.79) | 11.2 (9.8, 12.68) | 137.74 (76.81, 189.93) |
| **Rwanda** | 67.64 (51.2, 93.09) | 1.11 (0.8, 1.58) | 121.79 (93.54, 157.31) | 1.06 (0.78, 1.46) | -4.86 (-28.27, 23.6) |
| **Saint Kitts and Nevis** | 2.22 (2.01, 2.46) | 6.43 (5.84, 7.1) | 14.92 (10.91, 18.84) | 20.91 (15.42, 26.21) | 225.01 (136.77, 317.82) |
| **Saint Lucia** | 6.74 (6.11, 7.46) | 6.42 (5.9, 7.01) | 45.05 (36.39, 54.94) | 22.19 (17.9, 27.41) | 245.64 (173.96, 331.55) |
| **Saint Vincent and the Grenadines** | 5.83 (5.27, 6.55) | 6.91 (6.27, 7.65) | 21.76 (18.21, 25.94) | 16.85 (14, 20.25) | 143.97 (97.49, 199.02) |
| **Samoa** | 7.91 (5.09, 11.7) | 7.66 (4.83, 11.56) | 6.98 (4.79, 9.63) | 4.04 (2.77, 5.55) | -47.27 (-65.07, -19.4) |
| **San Marino** | 1.36 (1.12, 1.64) | 4.91 (4.03, 5.9) | 2.46 (1.76, 3.33) | 5.09 (3.67, 6.86) | 3.73 (-26.11, 45.72) |
| **Sao Tome and Principe** | 9.53 (6.58, 13.68) | 9.29 (6.62, 12.35) | 23.42 (13.57, 33.8) | 15.97 (9.25, 23.18) | 71.83 (-2.62, 175.01) |
| **Saudi Arabia** | 288.11 (196.56, 436.14) | 2.59 (1.6, 4.1) | 941.09 (675.91, 1306.39) | 3.08 (2.25, 4.17) | 18.68 (-34.99, 115.97) |
| **Senegal** | 145.24 (101.82, 202.27) | 2.87 (1.93, 4.18) | 216.87 (159.42, 277.98) | 1.89 (1.36, 2.5) | -34.16 (-57.02, -1.83) |
| **Serbia** | 314.9 (266.37, 371.75) | 2.87 (2.44, 3.34) | 324.81 (255.83, 403.11) | 2.71 (2.16, 3.32) | -5.49 (-26.26, 18.83) |
| **Seychelles** | 5.42 (4.46, 6.59) | 8.96 (7.35, 10.9) | 12.97 (8.07, 19.99) | 11.12 (7.05, 16.68) | 24.13 (-20.02, 84.36) |
| **Sierra Leone** | 65.61 (46.95, 91.76) | 2.35 (1.64, 3.28) | 100.13 (75.23, 129.65) | 1.63 (1.19, 2.19) | -30.78 (-53.4, 1.39) |
| **Singapore** | 366.89 (332.11, 412.33) | 14.41 (12.98, 16.22) | 826.73 (703.39, 947.3) | 12.45 (10.41, 14.58) | -13.62 (-30.48, 4.88) |
| **Slovakia** | 243.11 (214.33, 279.21) | 4.34 (3.84, 5) | 456.58 (350.66, 584.47) | 5.82 (4.54, 7.32) | 33.89 (3.32, 74.47) |
| **Slovenia** | 378.58 (302.58, 472.86) | 19.65 (16.17, 24.26) | 50.45 (40.13, 63.34) | 1.79 (1.42, 2.22) | -90.88 (-93.24, -87.91) |
| **Solomon Islands** | 10.61 (6.97, 16.17) | 5.54 (3.44, 8.76) | 15.62 (11.34, 20.87) | 3.52 (2.46, 4.84) | -36.37 (-57.35, -1.41) |
| **Somalia** | 48.92 (36.38, 67.08) | 0.8 (0.56, 1.19) | 141.03 (106.77, 183.92) | 0.75 (0.54, 1.05) | -6.07 (-24.44, 17.31) |
| **South Africa** | 635.01 (557.25, 717.63) | 2.17 (1.9, 2.46) | 874.07 (775.77, 982.64) | 1.64 (1.46, 1.84) | -24.55 (-33.74, -13.59) |
| **South Sudan** | 56.61 (39.05, 114.56) | 1.07 (0.74, 1.85) | 77.33 (58.86, 102.76) | 0.92 (0.67, 1.24) | -14.39 (-42.93, 11) |
| **Spain** | 13065.67 (12509.91, 13673.04) | 30.18 (28.45, 32.36) | 26304.5 (23472.06, 29130.52) | 35.32 (31.62, 38.99) | 17.01 (3.9, 30.78) |
| **Sri Lanka** | 1362.07 (1089.72, 1761.85) | 9.48 (7.65, 11.87) | 970.87 (685.99, 1337.46) | 3.99 (2.79, 5.6) | -57.89 (-72.08, -38.03) |
| **Sudan** | 254.34 (191.3, 358.23) | 1.76 (1.26, 2.63) | 607.73 (449.05, 804.2) | 2.06 (1.47, 2.84) | 17.25 (-23.45, 74.87) |
| **Suriname** | 20.37 (16.82, 23.84) | 6.35 (5.32, 7.34) | 88.74 (66.3, 116.79) | 14.88 (11.01, 19.83) | 134.21 (69.81, 221.18) |
| **Sweden** | 4450.45 (4217.87, 4671.71) | 34.73 (33.04, 36.37) | 7485.07 (6887.97, 8050.99) | 42.38 (39.39, 45.18) | 22.03 (12.87, 31.49) |
| **Switzerland** | 3417.32 (3151.15, 3687.17) | 37.37 (34.4, 40.24) | 4864.31 (4441.29, 5304.71) | 33.11 (30.38, 36.04) | -11.39 (-21.66, -0.39) |
| **Syrian Arab Republic** | 205.56 (164.48, 259.56) | 2.31 (1.77, 3.05) | 423.8 (277.93, 650.09) | 2.86 (1.88, 4.36) | 23.93 (-21.22, 93.23) |
| **Taiwan (Province of China)** | 1968.66 (1850.54, 2108.59) | 10.32 (9.71, 11.04) | 4594.86 (3482.25, 6021.46) | 15.15 (11.71, 19.39) | 46.74 (11.72, 90.68) |
| **Tajikistan** | 65.23 (53.33, 79.62) | 1.45 (1.16, 1.83) | 142.63 (116.59, 173.68) | 1.72 (1.39, 2.12) | 18.34 (-9.34, 53.8) |
| **Thailand** | 963.32 (815.9, 1151.01) | 2.02 (1.69, 2.41) | 1390.55 (1042.11, 1834.74) | 1.55 (1.21, 1.97) | -23.18 (-42.47, 1.67) |
| **Timor-Leste** | 13.1 (8.61, 22.76) | 2.09 (1.33, 3.09) | 25.39 (15.23, 35.22) | 2.4 (1.4, 3.47) | 14.79 (-30.03, 68.94) |
| **Togo** | 59.03 (46.4, 74.04) | 2.72 (2.06, 3.58) | 108.04 (79.7, 142.44) | 1.84 (1.31, 2.49) | -32.53 (-55.34, 2.5) |
| **Tokelau** | 0.1 (0.07, 0.14) | 7.26 (4.83, 10.28) | 0.06 (0.04, 0.09) | 4.3 (3.03, 5.91) | -40.74 (-61.52, -9.79) |
| **Tonga** | 3.7 (2.49, 5.46) | 5.5 (3.59, 8.22) | 3.11 (2.11, 4.45) | 3.62 (2.4, 5.23) | -34.2 (-54.93, -3.34) |
| **Trinidad and Tobago** | 53.37 (49.17, 58.05) | 5.48 (5.07, 5.93) | 208.51 (150.04, 277.66) | 11.3 (8.18, 15.05) | 106.17 (46.87, 177.74) |
| **Tunisia** | 160.75 (128.72, 202.69) | 2.3 (1.81, 2.99) | 345.7 (249.14, 472.81) | 2.67 (1.95, 3.64) | 15.91 (-24.02, 76.4) |
| **Turkey** | 27531.72 (15586.73, 49965.95) | 42.3 (26.97, 71.7) | 23574.32 (18647.03, 29384.9) | 31.73 (24.56, 40.41) | -25 (-57.96, 20.86) |
| **Turkmenistan** | 126.27 (111.36, 142.78) | 4.47 (4, 4.97) | 353.76 (257.82, 473.4) | 7.33 (5.4, 9.75) | 63.88 (21.54, 109.26) |
| **Tuvalu** | 0.59 (0.4, 0.8) | 7.35 (4.99, 9.96) | 0.45 (0.31, 0.64) | 3.99 (2.75, 5.65) | -45.7 (-62.68, -18.4) |
| **Uganda** | 125.59 (90.33, 185.92) | 0.84 (0.56, 1.31) | 343.64 (266.89, 441.61) | 0.96 (0.72, 1.3) | 14.19 (-16.54, 53.39) |
| **Ukraine** | 3108.1 (2605.95, 4189.45) | 5.14 (4.36, 6.79) | 5531.11 (4632.02, 6533.08) | 9.96 (8.39, 11.64) | 93.97 (36.74, 145.45) |
| **United Arab Emirates** | 52.84 (37.59, 111.69) | 4.84 (2.9, 11.49) | 561 (257.76, 1065.17) | 5.9 (2.56, 11.17) | 21.9 (-44.37, 124.63) |
| **United Kingdom** | 32791.41 (31832.18, 33579.25) | 44.14 (42.7, 45.29) | 54243.27 (50826.1, 57086.35) | 50.38 (47.59, 52.92) | 14.14 (7.78, 21.05) |
| **United Republic of Tanzania** | 260.17 (200.3, 357.17) | 1.06 (0.79, 1.47) | 608.57 (487.97, 759.18) | 1.13 (0.88, 1.43) | 5.87 (-18.27, 33.74) |
| **United States of America** | 103663.77 (100545.64, 106801.93) | 37.02 (35.88, 38.17) | 224864.74 (216809.12, 231546.99) | 46.88 (45.26, 48.33) | 26.64 (22.11, 31.23) |
| **United States Virgin Islands** | 12.43 (9.93, 15.47) | 12.51 (10.05, 15.68) | 52.13 (37.76, 67.27) | 31.6 (23.36, 40) | 152.58 (74.94, 251.99) |
| **Uruguay** | 279.9 (260.91, 299.78) | 8.02 (7.45, 8.6) | 1352.59 (1235.51, 1467.54) | 29.09 (26.59, 31.51) | 262.8 (224.21, 300.9) |
| **Uzbekistan** | 195.29 (156.81, 243.49) | 1.2 (0.98, 1.48) | 497.83 (411.35, 595.14) | 1.62 (1.35, 1.93) | 35.96 (6.98, 60.43) |
| **Vanuatu** | 4.26 (2.6, 6.88) | 4.97 (2.86, 8.44) | 7.37 (4.97, 10.98) | 3.42 (2.22, 5.21) | -31.11 (-53.72, 6.39) |
| **Venezuela (Bolivarian Republic of)** | 784.34 (724.23, 855.29) | 5.68 (5.28, 6.14) | 3533.75 (2532.59, 4713.96) | 11.66 (8.39, 15.56) | 105.21 (45.37, 175.32) |
| **Viet Nam** | 1537.73 (1132.6, 2091.4) | 3 (2.15, 4.2) | 3792.5 (2801.61, 5009.75) | 3.64 (2.72, 4.77) | 21.33 (-21.56, 83.72) |
| **Yemen** | 133.44 (96, 193.27) | 1.5 (1, 2.37) | 395.64 (299.7, 514.48) | 1.79 (1.33, 2.46) | 19.88 (-23.64, 83.13) |
| **Zambia** | 93.43 (71.27, 133.47) | 1.27 (1, 1.65) | 202.84 (162.64, 250.49) | 1.31 (1.04, 1.63) | 2.58 (-21.77, 32.68) |
| **Zimbabwe** | 298.39 (239.8, 368.36) | 5.03 (3.98, 6.23) | 657.67 (425.88, 1000.09) | 6.71 (4.18, 10.46) | 33.52 (-22.1, 128.2) |
| **YLD** | | | | | |
| **Afghanistan** | 40.91 (27.66, 57.5) | 0.41 (0.28, 0.57) | 138.42 (94.2, 194.59) | 0.41 (0.28, 0.57) | 0.15 (-4.02, 4.17) |
| **Albania** | 20.39 (13.87, 28.09) | 0.63 (0.43, 0.87) | 19.13 (13.16, 26.46) | 0.67 (0.47, 0.93) | 7.24 (3.15, 11.9) |
| **Algeria** | 111.57 (76.73, 155.87) | 0.49 (0.34, 0.68) | 211.84 (144.73, 295.83) | 0.5 (0.34, 0.7) | 2.02 (-1.59, 5.67) |
| **American Samoa** | 0.19 (0.13, 0.27) | 0.44 (0.3, 0.59) | 0.23 (0.15, 0.31) | 0.41 (0.28, 0.57) | -4.96 (-9.53, -0.35) |
| **Andorra** | 1.23 (0.85, 1.65) | 2.15 (1.5, 2.86) | 3.03 (2.07, 4.07) | 2.46 (1.71, 3.27) | 14.4 (8.2, 21.23) |
| **Angola** | 29.18 (20, 40.48) | 0.35 (0.24, 0.48) | 89.36 (59.89, 124.51) | 0.36 (0.25, 0.51) | 4.52 (0.57, 8.45) |
| **Antigua and Barbuda** | 0.33 (0.22, 0.45) | 0.55 (0.37, 0.76) | 0.54 (0.37, 0.73) | 0.59 (0.4, 0.8) | 6.67 (1.74, 11.11) |
| **Argentina** | 255.28 (174.65, 343.81) | 0.78 (0.53, 1.05) | 453.39 (314.21, 607.97) | 0.95 (0.65, 1.28) | 21.82 (16.65, 27.33) |
| **Armenia** | 19.88 (13.67, 27.38) | 0.59 (0.41, 0.81) | 18.81 (13, 26.15) | 0.61 (0.42, 0.84) | 3.39 (-0.68, 8.03) |
| **Australia** | 248.08 (170.6, 332.48) | 1.33 (0.92, 1.77) | 596.03 (411.45, 791.2) | 1.69 (1.18, 2.25) | 27.79 (20.02, 35.42) |
| **Austria** | 117.32 (82.26, 156.88) | 1.24 (0.87, 1.66) | 214.39 (148.75, 284.58) | 1.65 (1.14, 2.18) | 32.42 (24.3, 41.63) |
| **Azerbaijan** | 39.15 (26.95, 54.15) | 0.55 (0.38, 0.75) | 59.44 (40.58, 82.61) | 0.56 (0.39, 0.77) | 3.39 (-0.97, 7.86) |
| **Bahamas** | 1.61 (1.09, 2.17) | 0.65 (0.45, 0.87) | 2.49 (1.73, 3.38) | 0.65 (0.45, 0.87) | 0.03 (-4.43, 4.81) |
| **Bahrain** | 2.91 (1.98, 4.06) | 0.57 (0.39, 0.79) | 9.75 (6.67, 13.9) | 0.59 (0.4, 0.82) | 3.68 (-0.46, 7.9) |
| **Bangladesh** | 346.75 (234.36, 484.72) | 0.35 (0.24, 0.49) | 607.36 (414.82, 848.62) | 0.38 (0.26, 0.53) | 8.4 (4.25, 12.92) |
| **Barbados** | 1.79 (1.25, 2.41) | 0.68 (0.47, 0.91) | 2.57 (1.77, 3.43) | 0.73 (0.51, 0.97) | 6.52 (1.96, 11.24) |
| **Belarus** | 73.87 (50.32, 101.5) | 0.71 (0.48, 0.98) | 69.1 (47.62, 94.65) | 0.74 (0.5, 1.01) | 3.92 (-0.53, 8.31) |
| **Belgium** | 180.98 (124.1, 240.26) | 1.43 (0.99, 1.89) | 316.31 (215.1, 420.99) | 1.84 (1.26, 2.44) | 28.7 (20.93, 37.77) |
| **Belize** | 0.82 (0.55, 1.13) | 0.47 (0.32, 0.64) | 2.04 (1.41, 2.83) | 0.51 (0.35, 0.7) | 8.25 (3.71, 13.5) |
| **Benin** | 12.12 (8.26, 16.98) | 0.3 (0.21, 0.42) | 33.37 (22.65, 46.96) | 0.31 (0.21, 0.43) | 3.04 (-0.63, 7.13) |
| **Bermuda** | 0.64 (0.45, 0.85) | 1.05 (0.73, 1.4) | 0.79 (0.55, 1.06) | 0.99 (0.69, 1.32) | -5.48 (-9.65, -1.22) |
| **Bhutan** | 2.19 (1.5, 3.07) | 0.39 (0.27, 0.54) | 3.26 (2.22, 4.57) | 0.43 (0.29, 0.59) | 10.53 (5.85, 15.88) |
| **Bolivia (Plurinational State of)** | 22.42 (15.41, 31.09) | 0.39 (0.27, 0.54) | 49.23 (33.77, 67.67) | 0.43 (0.3, 0.59) | 11.41 (6.82, 15.87) |
| **Bosnia and Herzegovina** | 33.89 (22.79, 46.56) | 0.73 (0.5, 1) | 26.6 (18.14, 36.64) | 0.79 (0.53, 1.09) | 7.31 (2.65, 12.44) |
| **Botswana** | 4.39 (2.97, 6.09) | 0.37 (0.25, 0.5) | 8.7 (5.97, 12.21) | 0.39 (0.27, 0.54) | 6.13 (1.83, 10.82) |
| **Brazil** | 739.37 (503.16, 1032.63) | 0.51 (0.35, 0.71) | 1356.76 (940.06, 1852.22) | 0.6 (0.42, 0.82) | 17.14 (13.21, 21.71) |
| **Brunei Darussalam** | 0.98 (0.66, 1.36) | 0.48 (0.32, 0.65) | 2.1 (1.42, 2.92) | 0.51 (0.34, 0.69) | 6.82 (2.79, 10.84) |
| **Bulgaria** | 59.29 (40.76, 81.37) | 0.68 (0.47, 0.93) | 50.8 (34.97, 69.67) | 0.72 (0.5, 0.99) | 5.73 (0.78, 11.24) |
| **Burkina Faso** | 23.16 (15.78, 32.49) | 0.29 (0.2, 0.4) | 58.54 (39.61, 81.59) | 0.3 (0.21, 0.42) | 5.07 (1.1, 9.09) |
| **Burundi** | 12.45 (8.44, 17.52) | 0.27 (0.19, 0.38) | 26.86 (18.2, 37.7) | 0.27 (0.19, 0.38) | -0.16 (-3.6, 3.82) |
| **Cabo Verde** | 1.26 (0.85, 1.76) | 0.41 (0.28, 0.56) | 2.35 (1.62, 3.3) | 0.43 (0.29, 0.6) | 5.2 (1.44, 9.11) |
| **Cambodia** | 27.34 (18.71, 38.32) | 0.29 (0.2, 0.4) | 51.79 (35.69, 71.78) | 0.32 (0.22, 0.44) | 9.23 (4.91, 13.75) |
| **Cameroon** | 25.29 (17.03, 35.44) | 0.28 (0.2, 0.4) | 73.91 (50.62, 104.08) | 0.29 (0.2, 0.39) | 0.37 (-2.99, 4.18) |
| **Canada** | 647.94 (448.62, 863.27) | 2.19 (1.52, 2.9) | 1316.71 (911.4, 1735.37) | 2.54 (1.77, 3.32) | 16.41 (9.81, 23.42) |
| **Central African Republic** | 6.26 (4.23, 8.76) | 0.27 (0.19, 0.38) | 12.11 (8.27, 17.06) | 0.27 (0.19, 0.37) | -1.48 (-5.25, 2.58) |
| **Chad** | 15.72 (10.75, 21.76) | 0.31 (0.22, 0.43) | 43.48 (29.35, 61.11) | 0.33 (0.23, 0.46) | 4.48 (0.9, 8.5) |
| **Chile** | 118.17 (82.02, 159.33) | 0.94 (0.65, 1.26) | 233.45 (161.41, 310.57) | 1.15 (0.8, 1.54) | 22.83 (15.76, 29.11) |
| **China** | 6216.86 (4189.43, 8667.07) | 0.52 (0.36, 0.72) | 8870.65 (6084.41, 12429.81) | 0.6 (0.4, 0.83) | 14.34 (12.17, 17.02) |
| **Colombia** | 132.56 (89.84, 183.55) | 0.42 (0.29, 0.58) | 238.26 (165.18, 323.76) | 0.49 (0.34, 0.66) | 15.09 (9.23, 21.48) |
| **Comoros** | 1.41 (0.96, 1.99) | 0.36 (0.25, 0.51) | 2.43 (1.68, 3.41) | 0.36 (0.25, 0.51) | 0.21 (-3.25, 3.68) |
| **Congo** | 6.05 (4.05, 8.55) | 0.29 (0.2, 0.4) | 13.96 (9.48, 19.61) | 0.3 (0.2, 0.41) | 1.52 (-2.22, 5.55) |
| **Cook Islands** | 0.08 (0.06, 0.12) | 0.46 (0.31, 0.63) | 0.09 (0.06, 0.12) | 0.47 (0.32, 0.65) | 3.27 (-0.62, 8.42) |
| **Costa Rica** | 15.71 (10.82, 21.45) | 0.58 (0.4, 0.78) | 36.42 (25.64, 48.46) | 0.74 (0.52, 0.98) | 26.95 (20.89, 33.93) |
| **Croatia** | 46.32 (32.41, 62.42) | 0.93 (0.65, 1.27) | 45.05 (31.11, 60.65) | 0.99 (0.68, 1.33) | 5.74 (-1.37, 13.02) |
| **Cuba** | 70.22 (48.46, 94.76) | 0.64 (0.44, 0.87) | 86.46 (60.48, 116.21) | 0.68 (0.48, 0.92) | 6.35 (1.94, 11.66) |
| **Cyprus** | 8.05 (5.51, 10.78) | 0.99 (0.68, 1.32) | 19.48 (13.38, 26.18) | 1.18 (0.81, 1.56) | 19.19 (13.9, 24.8) |
| **Czechia** | 100.63 (69.88, 137.1) | 0.97 (0.67, 1.32) | 115.43 (80.2, 156.03) | 1.02 (0.7, 1.38) | 5.36 (-1.45, 12.97) |
| **Côte d'Ivoire** | 30.34 (20.29, 42.69) | 0.29 (0.2, 0.41) | 68.44 (46.22, 95.81) | 0.29 (0.2, 0.41) | 0.73 (-3.03, 4.23) |
| **Democratic People's Republic of Korea** | 115.34 (78.99, 158.24) | 0.57 (0.39, 0.78) | 159.61 (109.78, 219.03) | 0.58 (0.41, 0.8) | 2.14 (-2.3, 7.05) |
| **Democratic Republic of the Congo** | 90.7 (61.8, 127.48) | 0.29 (0.2, 0.4) | 209.71 (143.53, 295.26) | 0.28 (0.2, 0.4) | -1.84 (-5.25, 1.82) |
| **Denmark** | 116.18 (79.52, 154.56) | 1.73 (1.18, 2.32) | 173.68 (117.11, 230.47) | 1.96 (1.35, 2.61) | 13.48 (7.1, 19.74) |
| **Djibouti** | 1.46 (0.98, 2.03) | 0.37 (0.25, 0.51) | 4.01 (2.74, 5.63) | 0.37 (0.25, 0.51) | 0.11 (-3.14, 3.53) |
| **Dominica** | 0.38 (0.27, 0.51) | 0.52 (0.37, 0.71) | 0.46 (0.32, 0.62) | 0.63 (0.44, 0.85) | 21.23 (14.46, 28.95) |
| **Dominican Republic** | 31.55 (21.46, 43.9) | 0.47 (0.32, 0.65) | 54.42 (37.44, 74.96) | 0.5 (0.35, 0.69) | 7.73 (2.84, 12.51) |
| **Ecuador** | 31.64 (21.29, 44.4) | 0.34 (0.23, 0.47) | 67.07 (46.24, 92.54) | 0.39 (0.27, 0.53) | 14.62 (8.93, 20.34) |
| **Egypt** | 230.93 (156.83, 321.6) | 0.45 (0.31, 0.63) | 453.51 (308.25, 631.73) | 0.47 (0.32, 0.66) | 4.2 (0.57, 8.21) |
| **El Salvador** | 20.94 (14.15, 29.26) | 0.41 (0.29, 0.57) | 27.5 (18.91, 37.94) | 0.44 (0.3, 0.6) | 5.45 (1.32, 9.93) |
| **Equatorial Guinea** | 0.99 (0.67, 1.39) | 0.28 (0.2, 0.39) | 4.06 (2.72, 5.74) | 0.34 (0.23, 0.47) | 20.2 (14.96, 25.51) |
| **Eritrea** | 8.3 (5.65, 11.78) | 0.33 (0.23, 0.46) | 20.4 (13.96, 28.83) | 0.35 (0.24, 0.48) | 4.26 (0.33, 8.55) |
| **Estonia** | 12.78 (8.88, 17.38) | 0.81 (0.56, 1.11) | 11.21 (7.77, 15.19) | 0.85 (0.58, 1.16) | 3.88 (-1.43, 9.37) |
| **Eswatini** | 2.67 (1.79, 3.71) | 0.37 (0.26, 0.51) | 4.13 (2.84, 5.75) | 0.39 (0.27, 0.53) | 4.28 (0.26, 8.44) |
| **Ethiopia** | 126.81 (85.73, 180.15) | 0.3 (0.2, 0.42) | 295.68 (198.27, 417.5) | 0.32 (0.22, 0.45) | 7.46 (5.08, 10.15) |
| **Fiji** | 2.76 (1.86, 3.86) | 0.37 (0.26, 0.52) | 3.52 (2.38, 4.87) | 0.39 (0.27, 0.54) | 4.31 (0.46, 8.07) |
| **Finland** | 134.54 (92, 177.75) | 2.14 (1.46, 2.84) | 226.94 (150.81, 306.01) | 2.47 (1.7, 3.28) | 15.85 (8.96, 23.47) |
| **France** | 1173.72 (811.23, 1563.02) | 1.63 (1.13, 2.15) | 1964.59 (1343.06, 2627.33) | 1.93 (1.34, 2.57) | 17.93 (10.91, 25.8) |
| **Gabon** | 2.84 (1.91, 3.93) | 0.33 (0.23, 0.46) | 5.33 (3.56, 7.5) | 0.33 (0.23, 0.46) | 0.11 (-3.93, 3.89) |
| **Gambia** | 2.82 (1.9, 3.97) | 0.33 (0.23, 0.47) | 6.72 (4.59, 9.42) | 0.33 (0.23, 0.46) | -0.12 (-3.82, 4.03) |
| **Georgia** | 32.86 (22.63, 45.17) | 0.6 (0.41, 0.82) | 22.36 (15.39, 30.72) | 0.6 (0.41, 0.83) | 1.13 (-3.34, 5.58) |
| **Germany** | 1314.85 (914.23, 1737.62) | 1.29 (0.9, 1.7) | 2270.84 (1534, 3029.02) | 1.66 (1.13, 2.21) | 28.43 (20.87, 36.54) |
| **Ghana** | 38.59 (26.01, 54.52) | 0.29 (0.2, 0.41) | 90.51 (61.64, 126.86) | 0.31 (0.21, 0.43) | 5.25 (1.08, 9.55) |
| **Greece** | 105.33 (73.36, 141) | 0.86 (0.6, 1.15) | 167.82 (116.56, 225.65) | 1.13 (0.79, 1.5) | 30.64 (21.37, 39.78) |
| **Greenland** | 0.88 (0.61, 1.18) | 1.81 (1.28, 2.41) | 1.23 (0.84, 1.66) | 2.02 (1.4, 2.69) | 11.64 (6.34, 16.69) |
| **Grenada** | 0.39 (0.27, 0.54) | 0.47 (0.32, 0.64) | 0.58 (0.4, 0.78) | 0.54 (0.37, 0.73) | 15.07 (9.42, 21.17) |
| **Guam** | 0.71 (0.49, 0.98) | 0.6 (0.41, 0.81) | 0.8 (0.55, 1.09) | 0.46 (0.32, 0.63) | -22.7 (-28.05, -16.77) |
| **Guatemala** | 29.09 (19.6, 40.44) | 0.39 (0.27, 0.55) | 71.03 (48.24, 99.27) | 0.41 (0.28, 0.57) | 3.46 (-0.45, 7.59) |
| **Guinea** | 15.6 (10.64, 21.77) | 0.3 (0.21, 0.41) | 32.29 (21.98, 45.18) | 0.3 (0.21, 0.41) | 0.93 (-2.83, 4.7) |
| **Guinea-Bissau** | 2.49 (1.67, 3.51) | 0.29 (0.2, 0.4) | 4.91 (3.36, 6.99) | 0.29 (0.2, 0.4) | 1.49 (-2.51, 5.42) |
| **Guyana** | 2.57 (1.75, 3.61) | 0.34 (0.24, 0.47) | 2.89 (2, 3.97) | 0.38 (0.26, 0.51) | 9.59 (4.63, 14.5) |
| **Haiti** | 21.08 (14.41, 29.14) | 0.36 (0.25, 0.5) | 43.73 (30.08, 60.26) | 0.38 (0.26, 0.51) | 3.86 (-0.54, 8.16) |
| **Honduras** | 18.14 (12.18, 25.47) | 0.41 (0.28, 0.56) | 41.74 (28.4, 58.4) | 0.44 (0.3, 0.6) | 7.1 (2.65, 12.12) |
| **Hungary** | 90.63 (62.89, 121.67) | 0.86 (0.6, 1.17) | 97.35 (67.25, 130.04) | 0.97 (0.66, 1.3) | 11.91 (2.62, 24.6) |
| **Iceland** | 5.15 (3.61, 6.85) | 1.91 (1.34, 2.53) | 8.1 (5.58, 10.81) | 1.87 (1.3, 2.48) | -2.22 (-7.84, 3.87) |
| **India** | 2789.7 (1878.35, 3927.14) | 0.34 (0.23, 0.47) | 5318.54 (3634.4, 7463.09) | 0.37 (0.26, 0.52) | 11.15 (9.45, 13.21) |
| **Indonesia** | 537.94 (365.15, 754.36) | 0.3 (0.21, 0.42) | 845.6 (573.4, 1189.61) | 0.32 (0.22, 0.45) | 7.03 (5.4, 8.75) |
| **Iran (Islamic Republic of)** | 289.33 (194.56, 403.46) | 0.57 (0.39, 0.79) | 540.7 (369.68, 757.72) | 0.61 (0.41, 0.85) | 6.58 (4.88, 8.41) |
| **Iraq** | 80.42 (54.47, 111.81) | 0.52 (0.36, 0.72) | 217.66 (147.76, 303.17) | 0.53 (0.37, 0.74) | 2.62 (-1.23, 6.4) |
| **Ireland** | 69.74 (48.02, 92.83) | 1.79 (1.23, 2.39) | 150.72 (102.62, 200.57) | 2.32 (1.6, 3.08) | 29.47 (21.28, 37.6) |
| **Israel** | 52.77 (36.57, 69.83) | 1.09 (0.76, 1.43) | 120.31 (82.79, 160) | 1.19 (0.82, 1.58) | 9.26 (3.93, 14.11) |
| **Italy** | 875.46 (597.31, 1162.93) | 1.18 (0.82, 1.58) | 1654.91 (1126.85, 2192.29) | 1.61 (1.11, 2.15) | 36.47 (30.12, 44.47) |
| **Jamaica** | 12.89 (8.91, 17.58) | 0.56 (0.39, 0.75) | 17.1 (11.79, 23.14) | 0.59 (0.41, 0.8) | 6.59 (1.93, 11.97) |
| **Japan** | 1425.1 (976.66, 1902.14) | 0.99 (0.68, 1.33) | 2269.73 (1540.39, 3049.24) | 1.13 (0.78, 1.51) | 13.81 (10.24, 17.54) |
| **Jordan** | 18.58 (12.64, 26.05) | 0.55 (0.38, 0.77) | 65.42 (44.92, 90.92) | 0.57 (0.39, 0.8) | 3.01 (-1.65, 7.19) |
| **Kazakhstan** | 98.85 (68.06, 137.34) | 0.61 (0.42, 0.84) | 115.25 (78.4, 159.85) | 0.63 (0.43, 0.87) | 4.03 (0.17, 8.43) |
| **Kenya** | 58.83 (39.42, 83.86) | 0.3 (0.2, 0.41) | 140.11 (94.73, 197.83) | 0.3 (0.21, 0.42) | 2.95 (2.11, 3.85) |
| **Kiribati** | 0.17 (0.11, 0.24) | 0.24 (0.17, 0.33) | 0.28 (0.19, 0.39) | 0.25 (0.17, 0.34) | 1.67 (-2.5, 5.7) |
| **Kuwait** | 12.07 (8.3, 16.69) | 0.69 (0.48, 0.95) | 32.2 (21.87, 45.47) | 0.66 (0.46, 0.91) | -4.15 (-9.8, 1.14) |
| **Kyrgyzstan** | 23.27 (15.8, 32.46) | 0.53 (0.36, 0.73) | 34.58 (23.57, 48.16) | 0.53 (0.37, 0.74) | 0.04 (-3.97, 3.98) |
| **Lao People's Democratic Republic** | 11.93 (8.17, 16.67) | 0.32 (0.22, 0.44) | 24.19 (16.7, 33.65) | 0.35 (0.24, 0.49) | 11 (6.6, 16.43) |
| **Latvia** | 20.46 (13.9, 28.25) | 0.77 (0.52, 1.07) | 16.29 (11.23, 21.81) | 0.82 (0.56, 1.13) | 6.64 (1.91, 12.22) |
| **Lebanon** | 17.72 (12.07, 24.43) | 0.58 (0.4, 0.8) | 31.4 (21.77, 43.61) | 0.6 (0.41, 0.82) | 2.83 (-0.99, 6.92) |
| **Lesotho** | 5.94 (4.05, 8.28) | 0.35 (0.24, 0.49) | 7.5 (5.18, 10.42) | 0.37 (0.26, 0.51) | 5.64 (1.3, 10.19) |
| **Liberia** | 4.8 (3.24, 6.72) | 0.28 (0.19, 0.39) | 12.04 (8.33, 17.02) | 0.28 (0.2, 0.39) | -0.6 (-4.35, 3.26) |
| **Libya** | 19.2 (13.13, 26.76) | 0.51 (0.35, 0.71) | 34.88 (23.95, 48.61) | 0.49 (0.34, 0.68) | -4.91 (-8.63, -0.95) |
| **Lithuania** | 28.87 (19.78, 39.18) | 0.78 (0.54, 1.06) | 30.99 (21.31, 40.96) | 0.97 (0.67, 1.31) | 24.42 (16.34, 32.48) |
| **Luxembourg** | 6.81 (4.66, 9.1) | 1.45 (1, 1.93) | 14.69 (10.11, 19.44) | 1.79 (1.25, 2.37) | 23.69 (17.19, 30.27) |
| **Madagascar** | 37.81 (25.67, 52.6) | 0.39 (0.27, 0.54) | 87.79 (60.06, 121.82) | 0.39 (0.27, 0.54) | 0.4 (-3.1, 3.69) |
| **Malawi** | 24.7 (16.79, 34.13) | 0.31 (0.22, 0.43) | 50.29 (34.1, 70.79) | 0.32 (0.22, 0.45) | 2.24 (-1.75, 6.34) |
| **Malaysia** | 57.51 (38.45, 80.31) | 0.34 (0.23, 0.48) | 117.38 (78.65, 163.62) | 0.37 (0.25, 0.52) | 8.02 (3.76, 12.16) |
| **Maldives** | 0.62 (0.41, 0.88) | 0.32 (0.22, 0.45) | 1.8 (1.22, 2.51) | 0.36 (0.24, 0.49) | 9.6 (5.61, 14.3) |
| **Mali** | 23.52 (16.08, 32.72) | 0.33 (0.23, 0.45) | 61.73 (42.39, 86.11) | 0.34 (0.24, 0.48) | 4.74 (1.12, 8.83) |
| **Malta** | 4.68 (3.2, 6.27) | 1.15 (0.79, 1.53) | 10.37 (7.12, 13.96) | 1.49 (1.03, 2) | 29.82 (23.75, 37.52) |
| **Marshall Islands** | 0.13 (0.09, 0.18) | 0.31 (0.21, 0.42) | 0.17 (0.12, 0.24) | 0.32 (0.22, 0.43) | 2.24 (-1.38, 5.92) |
| **Mauritania** | 6.96 (4.73, 9.78) | 0.39 (0.27, 0.54) | 14.44 (9.84, 20.12) | 0.4 (0.28, 0.55) | 2.47 (-1.46, 6.41) |
| **Mauritius** | 4.81 (3.22, 6.64) | 0.44 (0.3, 0.6) | 6.45 (4.43, 8.89) | 0.48 (0.33, 0.66) | 9.95 (5.17, 15.09) |
| **Mexico** | 442.06 (297.51, 613.83) | 0.53 (0.36, 0.74) | 735.55 (504.26, 1019.09) | 0.58 (0.4, 0.8) | 9.39 (6.94, 12.05) |
| **Micronesia (Federated States of)** | 0.29 (0.19, 0.41) | 0.3 (0.2, 0.41) | 0.3 (0.21, 0.42) | 0.3 (0.21, 0.41) | 2.05 (-2.68, 6.81) |
| **Monaco** | 0.85 (0.59, 1.14) | 1.66 (1.16, 2.21) | 1.46 (0.99, 1.96) | 2.06 (1.43, 2.76) | 24.12 (15.46, 33.65) |
| **Mongolia** | 11.23 (7.63, 15.5) | 0.54 (0.37, 0.75) | 19.28 (13.05, 26.89) | 0.57 (0.39, 0.8) | 6.29 (1.6, 10.94) |
| **Montenegro** | 4.63 (3.14, 6.38) | 0.73 (0.5, 1.01) | 4.76 (3.23, 6.56) | 0.75 (0.51, 1.04) | 2.81 (-1.58, 7.44) |
| **Morocco** | 112.97 (75.47, 157.81) | 0.48 (0.33, 0.67) | 183.33 (125.48, 256.39) | 0.5 (0.35, 0.7) | 3.96 (-0.01, 7.97) |
| **Mozambique** | 35.27 (24.03, 49.42) | 0.32 (0.23, 0.45) | 83.58 (56.63, 117.49) | 0.35 (0.24, 0.48) | 6.89 (2.9, 11.4) |
| **Myanmar** | 123.89 (84.48, 173.08) | 0.32 (0.22, 0.44) | 196.94 (135.67, 272.66) | 0.36 (0.25, 0.5) | 13.41 (8.59, 18.2) |
| **Namibia** | 4.89 (3.29, 6.82) | 0.38 (0.26, 0.52) | 8.94 (6.09, 12.45) | 0.39 (0.27, 0.54) | 4.47 (0.73, 8.62) |
| **Nauru** | 0.03 (0.02, 0.04) | 0.29 (0.19, 0.39) | 0.03 (0.02, 0.04) | 0.28 (0.19, 0.38) | -3.11 (-6.82, 0.92) |
| **Nepal** | 65.8 (44.88, 92.02) | 0.37 (0.25, 0.51) | 117.35 (80.89, 162.94) | 0.39 (0.27, 0.54) | 6.33 (1.96, 10.62) |
| **Netherlands** | 316.89 (218.38, 424.64) | 1.76 (1.22, 2.34) | 581.93 (402.59, 780.23) | 2.15 (1.49, 2.86) | 22.14 (14.02, 31.23) |
| **New Zealand** | 55.25 (38.21, 72.98) | 1.48 (1.02, 1.95) | 113.53 (76.87, 152.16) | 1.77 (1.22, 2.36) | 20.18 (13.68, 27.07) |
| **Nicaragua** | 15.09 (10.2, 21.21) | 0.43 (0.29, 0.59) | 28.21 (19.36, 39.05) | 0.44 (0.3, 0.6) | 3.04 (-1.44, 7.42) |
| **Niger** | 21.05 (14.47, 29.29) | 0.33 (0.23, 0.46) | 61.04 (41.46, 84.95) | 0.33 (0.23, 0.47) | 1.43 (-2, 5.3) |
| **Nigeria** | 242.98 (164.03, 342.04) | 0.31 (0.21, 0.43) | 608.01 (409.18, 862.36) | 0.32 (0.22, 0.45) | 4.55 (3.43, 5.64) |
| **Niue** | 0.01 (0.01, 0.01) | 0.41 (0.28, 0.56) | 0.01 (0, 0.01) | 0.42 (0.29, 0.58) | 2.44 (-1.73, 6.97) |
| **North Macedonia** | 14.63 (10, 20.04) | 0.72 (0.49, 0.99) | 15.61 (10.65, 21.62) | 0.7 (0.48, 0.97) | -2.13 (-8.85, 5.03) |
| **Northern Mariana Islands** | 0.2 (0.13, 0.28) | 0.45 (0.3, 0.62) | 0.19 (0.13, 0.27) | 0.45 (0.3, 0.61) | -0.48 (-4.42, 3.8) |
| **Norway** | 104.29 (71.5, 138.25) | 1.85 (1.27, 2.44) | 167.22 (114.07, 222.73) | 2.16 (1.48, 2.86) | 17.14 (14.25, 20.07) |
| **Oman** | 8.68 (5.89, 12.18) | 0.48 (0.33, 0.67) | 24.54 (16.82, 34.39) | 0.49 (0.34, 0.68) | 2.72 (-1.33, 6.85) |
| **Pakistan** | 383.45 (258.88, 538.3) | 0.37 (0.25, 0.51) | 841.08 (569.12, 1178.77) | 0.39 (0.26, 0.54) | 5.05 (2.25, 8.06) |
| **Palau** | 0.05 (0.03, 0.07) | 0.34 (0.23, 0.46) | 0.06 (0.04, 0.09) | 0.34 (0.23, 0.47) | 1.27 (-2.87, 5.62) |
| **Palestine** | 9.24 (6.26, 12.79) | 0.52 (0.36, 0.73) | 25.43 (17.2, 35.38) | 0.55 (0.37, 0.76) | 4.02 (0.03, 7.74) |
| **Panama** | 10.33 (6.98, 14.34) | 0.45 (0.31, 0.63) | 20.79 (14.27, 28.38) | 0.5 (0.34, 0.68) | 9.68 (5.11, 14.78) |
| **Papua New Guinea** | 10.32 (6.91, 14.57) | 0.27 (0.19, 0.37) | 25.89 (17.71, 36.66) | 0.28 (0.19, 0.39) | 3.06 (-0.72, 6.99) |
| **Paraguay** | 20.97 (14.16, 29.19) | 0.56 (0.38, 0.77) | 41 (28.48, 56.46) | 0.6 (0.42, 0.82) | 7.61 (2.55, 13.14) |
| **Peru** | 77.35 (52.48, 107.81) | 0.38 (0.26, 0.53) | 137.77 (95.29, 189.82) | 0.4 (0.28, 0.56) | 4.73 (0.1, 8.86) |
| **Philippines** | 204.06 (137.63, 287.46) | 0.34 (0.23, 0.47) | 396.27 (268.63, 553.44) | 0.36 (0.24, 0.5) | 5.87 (4.83, 7.05) |
| **Poland** | 345.83 (236.51, 473.64) | 0.88 (0.61, 1.2) | 439.32 (298.57, 589.42) | 1.01 (0.69, 1.38) | 14.25 (11.51, 17.26) |
| **Portugal** | 117.08 (81.47, 156.68) | 0.99 (0.69, 1.33) | 222.44 (152.53, 296.35) | 1.36 (0.94, 1.81) | 38.11 (29.56, 47.99) |
| **Puerto Rico** | 22.98 (15.8, 31.11) | 0.63 (0.43, 0.85) | 30.34 (21.04, 40.42) | 0.74 (0.51, 0.99) | 16.93 (11.31, 23.41) |
| **Qatar** | 2.71 (1.84, 3.83) | 0.58 (0.4, 0.81) | 19.79 (13.39, 28.07) | 0.6 (0.41, 0.84) | 4.16 (-0.32, 8.66) |
| **Republic of Korea** | 296.72 (202.68, 407.25) | 0.71 (0.48, 0.97) | 593.9 (401.97, 818.69) | 0.89 (0.6, 1.21) | 25.89 (19.81, 32.84) |
| **Republic of Moldova** | 27.27 (18.71, 37.73) | 0.61 (0.42, 0.85) | 22.93 (15.85, 31.79) | 0.62 (0.42, 0.85) | 0.52 (-4.16, 5.93) |
| **Romania** | 161.18 (110.97, 222.01) | 0.68 (0.47, 0.94) | 142.46 (98.67, 195.2) | 0.72 (0.49, 0.99) | 5.31 (0.87, 9.99) |
| **Russian Federation** | 1069.71 (734.77, 1489.93) | 0.71 (0.48, 0.98) | 1107.71 (755.62, 1528.41) | 0.75 (0.51, 1.03) | 5.37 (3.52, 7.56) |
| **Rwanda** | 16.23 (10.92, 22.95) | 0.27 (0.19, 0.38) | 32.08 (22.11, 44.86) | 0.29 (0.2, 0.4) | 4.67 (0.92, 9.16) |
| **Saint Kitts and Nevis** | 0.22 (0.15, 0.3) | 0.52 (0.36, 0.72) | 0.36 (0.25, 0.48) | 0.58 (0.41, 0.78) | 11.09 (6.38, 16.56) |
| **Saint Lucia** | 0.69 (0.47, 0.94) | 0.53 (0.37, 0.71) | 1.1 (0.77, 1.49) | 0.59 (0.41, 0.8) | 12.18 (6.9, 17.75) |
| **Saint Vincent and the Grenadines** | 0.52 (0.35, 0.71) | 0.49 (0.34, 0.66) | 0.64 (0.45, 0.87) | 0.54 (0.38, 0.73) | 11.23 (6.45, 16.53) |
| **Samoa** | 0.57 (0.38, 0.79) | 0.37 (0.25, 0.5) | 0.77 (0.51, 1.08) | 0.37 (0.25, 0.52) | 2.17 (-1.72, 6.38) |
| **San Marino** | 0.47 (0.33, 0.62) | 1.61 (1.12, 2.11) | 0.95 (0.65, 1.28) | 1.92 (1.33, 2.54) | 19.39 (11.67, 28.31) |
| **Sao Tome and Principe** | 0.3 (0.2, 0.43) | 0.29 (0.2, 0.41) | 0.57 (0.38, 0.79) | 0.3 (0.2, 0.42) | 1.99 (-1.62, 5.9) |
| **Saudi Arabia** | 73.81 (50.6, 102.99) | 0.5 (0.35, 0.69) | 197 (134.58, 277.49) | 0.51 (0.35, 0.71) | 2.86 (-0.9, 6.81) |
| **Senegal** | 21.88 (14.73, 30.63) | 0.34 (0.23, 0.47) | 47.15 (32.41, 65.65) | 0.35 (0.24, 0.49) | 2.5 (-1.72, 6.74) |
| **Serbia** | 67.27 (45.98, 91.98) | 0.71 (0.48, 0.96) | 65.31 (44.96, 90.45) | 0.73 (0.51, 1.02) | 3.76 (-1.01, 8.96) |
| **Seychelles** | 0.26 (0.17, 0.36) | 0.36 (0.25, 0.5) | 0.4 (0.27, 0.56) | 0.38 (0.26, 0.53) | 5.96 (1.97, 9.95) |
| **Sierra Leone** | 9.04 (6.17, 12.54) | 0.29 (0.2, 0.4) | 20.9 (14.29, 29.13) | 0.29 (0.2, 0.4) | 0.26 (-3.25, 4) |
| **Singapore** | 16.71 (11.46, 22.76) | 0.57 (0.4, 0.78) | 43.4 (29.83, 58.69) | 0.67 (0.46, 0.91) | 16.31 (8.96, 24.98) |
| **Slovakia** | 42 (28.85, 57.54) | 0.79 (0.54, 1.08) | 46.59 (31.88, 63.96) | 0.84 (0.57, 1.15) | 6.87 (2.08, 12.17) |
| **Slovenia** | 18.76 (13.01, 25.34) | 0.93 (0.64, 1.25) | 18.56 (12.56, 25.55) | 0.86 (0.58, 1.19) | -7.39 (-16.47, 1.14) |
| **Solomon Islands** | 0.86 (0.58, 1.23) | 0.27 (0.18, 0.37) | 1.72 (1.16, 2.43) | 0.27 (0.19, 0.38) | 2.71 (-1.51, 7.09) |
| **Somalia** | 15.49 (10.5, 21.96) | 0.27 (0.18, 0.37) | 43 (29.33, 60.48) | 0.26 (0.18, 0.36) | -2.14 (-5.75, 1.98) |
| **South Africa** | 143.5 (97.68, 200.94) | 0.4 (0.28, 0.56) | 236.06 (161.37, 329.26) | 0.43 (0.29, 0.59) | 5.67 (4.06, 7.41) |
| **South Sudan** | 17.14 (11.75, 23.89) | 0.36 (0.25, 0.5) | 27.42 (18.56, 38.2) | 0.36 (0.25, 0.5) | 0.24 (-3.13, 4.21) |
| **Spain** | 555.96 (383.42, 737.81) | 1.18 (0.82, 1.55) | 1058.1 (729.71, 1402.13) | 1.5 (1.04, 1.99) | 27.52 (19.64, 36.71) |
| **Sri Lanka** | 58.98 (39.64, 82.15) | 0.35 (0.24, 0.48) | 86.78 (58.89, 120.18) | 0.39 (0.26, 0.53) | 10.19 (5.1, 14.82) |
| **Sudan** | 60.56 (41.32, 84.19) | 0.34 (0.24, 0.48) | 135.31 (91.14, 188.06) | 0.36 (0.25, 0.5) | 4.06 (0.39, 8.21) |
| **Suriname** | 1.5 (1.03, 2.07) | 0.4 (0.28, 0.55) | 2.55 (1.78, 3.47) | 0.44 (0.3, 0.59) | 8.83 (3.95, 13.54) |
| **Sweden** | 259.67 (176.49, 349.13) | 2.1 (1.43, 2.83) | 369.98 (255.57, 494.26) | 2.32 (1.6, 3.08) | 10.76 (5.49, 16.93) |
| **Switzerland** | 151.31 (102.95, 201.64) | 1.7 (1.17, 2.25) | 251.77 (173.24, 336.34) | 1.87 (1.3, 2.48) | 10.21 (4.47, 16.29) |
| **Syrian Arab Republic** | 60.76 (40.91, 85.37) | 0.54 (0.37, 0.75) | 77.97 (53.2, 109.9) | 0.54 (0.37, 0.76) | 0.45 (-3.38, 4.57) |
| **Taiwan (Province of China)** | 129.33 (87.6, 176.91) | 0.63 (0.43, 0.87) | 183.51 (126.38, 246.86) | 0.7 (0.48, 0.95) | 10.55 (4.93, 16.11) |
| **Tajikistan** | 25.49 (17.28, 35.59) | 0.51 (0.35, 0.7) | 46.51 (31.81, 64.95) | 0.5 (0.34, 0.69) | -1.71 (-5.97, 2.35) |
| **Thailand** | 213.74 (144.99, 297.67) | 0.39 (0.27, 0.54) | 321.9 (218.93, 446.51) | 0.42 (0.29, 0.59) | 9.9 (4.99, 14.59) |
| **Timor-Leste** | 2.09 (1.42, 2.99) | 0.3 (0.21, 0.42) | 4 (2.72, 5.63) | 0.32 (0.22, 0.44) | 7.06 (2.89, 11.38) |
| **Togo** | 9.04 (6.11, 12.75) | 0.29 (0.2, 0.41) | 21.02 (14.4, 29.3) | 0.3 (0.2, 0.41) | 1.07 (-2.74, 5.08) |
| **Tokelau** | 0.01 (0, 0.01) | 0.33 (0.23, 0.46) | 0 (0, 0.01) | 0.35 (0.24, 0.48) | 5.35 (0.41, 10.35) |
| **Tonga** | 0.38 (0.25, 0.52) | 0.41 (0.28, 0.56) | 0.42 (0.29, 0.58) | 0.43 (0.29, 0.59) | 3.7 (-0.3, 8.1) |
| **Trinidad and Tobago** | 5.63 (3.85, 7.73) | 0.48 (0.33, 0.65) | 7.78 (5.4, 10.56) | 0.53 (0.37, 0.71) | 9 (4.19, 14.87) |
| **Tunisia** | 43.83 (29.69, 60.45) | 0.56 (0.38, 0.77) | 70.61 (48.52, 99.23) | 0.58 (0.4, 0.81) | 4.09 (-0.53, 8.05) |
| **Turkey** | 379.17 (262.74, 521.67) | 0.69 (0.48, 0.94) | 672.09 (464.13, 915.62) | 0.76 (0.53, 1.04) | 10.23 (6.21, 14.49) |
| **Turkmenistan** | 18.13 (12.35, 25.26) | 0.51 (0.35, 0.71) | 27.22 (18.62, 37.75) | 0.54 (0.37, 0.75) | 5.4 (1.09, 9.61) |
| **Tuvalu** | 0.03 (0.02, 0.04) | 0.28 (0.2, 0.39) | 0.04 (0.02, 0.05) | 0.3 (0.2, 0.41) | 5.38 (0.75, 10.07) |
| **Uganda** | 37.03 (24.87, 51.79) | 0.26 (0.18, 0.37) | 93.97 (63.56, 133.33) | 0.28 (0.19, 0.38) | 5.39 (1.12, 9.58) |
| **Ukraine** | 337.9 (229.25, 472.48) | 0.65 (0.44, 0.91) | 277.24 (190.95, 385.26) | 0.64 (0.44, 0.89) | -1.73 (-6.23, 2.76) |
| **United Arab Emirates** | 11.04 (7.47, 15.44) | 0.57 (0.39, 0.79) | 63.18 (42.67, 91.5) | 0.57 (0.39, 0.79) | -0.55 (-4.43, 4.52) |
| **United Kingdom** | 1237.77 (846.71, 1636.03) | 1.61 (1.11, 2.13) | 2083.14 (1435.11, 2767.67) | 2.04 (1.4, 2.69) | 26.62 (23.38, 29.9) |
| **United Republic of Tanzania** | 65.1 (44.21, 92.09) | 0.31 (0.21, 0.43) | 152.49 (103.23, 213.65) | 0.32 (0.22, 0.44) | 4.37 (0.53, 8.52) |
| **United States of America** | 4509.64 (3138.79, 5949.06) | 1.58 (1.1, 2.09) | 8015.62 (5763.69, 10512.14) | 1.81 (1.3, 2.37) | 14.55 (4.85, 26.41) |
| **United States Virgin Islands** | 0.7 (0.49, 0.94) | 0.69 (0.48, 0.93) | 1.13 (0.77, 1.51) | 0.85 (0.59, 1.14) | 23.66 (17.76, 29.99) |
| **Uruguay** | 30.94 (21.21, 40.99) | 0.91 (0.63, 1.22) | 45.64 (31.47, 60.2) | 1.11 (0.77, 1.47) | 21.33 (15.44, 27.02) |
| **Uzbekistan** | 105.9 (73, 146.31) | 0.53 (0.36, 0.73) | 184.04 (125.13, 257.96) | 0.55 (0.37, 0.76) | 4.18 (-0.02, 8.75) |
| **Vanuatu** | 0.48 (0.32, 0.68) | 0.34 (0.24, 0.47) | 0.99 (0.67, 1.37) | 0.35 (0.24, 0.48) | 1.72 (-2.23, 6.25) |
| **Venezuela (Bolivarian Republic of)** | 77.22 (52.07, 105.81) | 0.44 (0.3, 0.59) | 130.45 (89.94, 177.48) | 0.46 (0.31, 0.62) | 4.48 (0.28, 8.78) |
| **Viet Nam** | 236.93 (160.74, 329.56) | 0.37 (0.25, 0.5) | 397.74 (272.51, 551.23) | 0.4 (0.28, 0.55) | 10.36 (6.11, 15) |
| **Yemen** | 39.49 (26.93, 55.17) | 0.35 (0.24, 0.48) | 99.91 (67.76, 140.43) | 0.34 (0.24, 0.48) | -0.76 (-4.46, 3.15) |
| **Zambia** | 21.51 (14.57, 30.38) | 0.33 (0.23, 0.46) | 52.98 (36.14, 74.42) | 0.34 (0.24, 0.47) | 4.42 (1.15, 8.28) |
| **Zimbabwe** | 30.34 (20.32, 42.97) | 0.32 (0.22, 0.44) | 43.97 (29.71, 62.11) | 0.31 (0.22, 0.43) | -2.86 (-6.47, 1.32) |
| **YLL** | | | | | |
| **Afghanistan** | 216.01 (142.48, 354.86) | 2.36 (1.49, 3.98) | 489.61 (352.88, 677.21) | 2.17 (1.46, 3.17) | -8.17 (-39.2, 41.1) |
| **Albania** | 122.72 (109.78, 137.51) | 4.52 (4.05, 5.01) | 179.34 (131.07, 238.03) | 5.36 (4, 6.99) | 18.75 (-12.73, 58) |
| **Algeria** | 362.25 (266.98, 497.18) | 2.1 (1.48, 2.95) | 811.25 (612.96, 1058.46) | 2.04 (1.54, 2.67) | -2.97 (-34.49, 46.35) |
| **American Samoa** | 2.32 (1.74, 3.02) | 8.3 (6.18, 10.82) | 1.4 (1.03, 1.81) | 2.54 (1.89, 3.28) | -69.36 (-79.55, -54.15) |
| **Andorra** | 25.7 (15.76, 39.76) | 48.91 (30.16, 74.85) | 66.25 (41.21, 101.7) | 51.99 (32.57, 79.31) | 6.3 (-33.06, 70.65) |
| **Angola** | 65.15 (42.81, 98.31) | 0.74 (0.45, 1.15) | 193.16 (139.09, 250.19) | 0.76 (0.53, 1.02) | 2.89 (-33.61, 57.8) |
| **Antigua and Barbuda** | 2.22 (2.01, 2.45) | 4.39 (3.97, 4.85) | 11.46 (9.18, 13.98) | 11.48 (9.13, 14.22) | 161.31 (103.82, 229.98) |
| **Argentina** | 1747.13 (1635.13, 1870.47) | 5.33 (4.99, 5.71) | 9820.78 (8855.16, 10570.05) | 19.57 (17.65, 21.13) | 267.38 (230.4, 305.47) |
| **Armenia** | 66.22 (55.99, 88.08) | 2.14 (1.82, 2.86) | 58.09 (47.05, 69.63) | 1.43 (1.15, 1.71) | -33.3 (-52.37, -14.14) |
| **Australia** | 8085.25 (7673.77, 8488.01) | 44.85 (42.37, 47.39) | 18900.38 (16963.88, 20790.89) | 53.85 (48.35, 59.45) | 20.07 (6.02, 34.35) |
| **Austria** | 2131.21 (1993.33, 2267.35) | 24.9 (22.78, 27.29) | 4335.83 (3979.25, 4706.88) | 32.97 (30.08, 36.1) | 32.38 (17.08, 50.12) |
| **Azerbaijan** | 84.6 (67.79, 106.4) | 1.34 (1.06, 1.73) | 192.6 (146.09, 254.37) | 1.66 (1.3, 2.16) | 23.75 (-10.64, 72.28) |
| **Bahamas** | 13.34 (11.75, 15.18) | 6.88 (6.12, 7.77) | 63.9 (49.53, 82.02) | 15.72 (12.13, 20.11) | 128.46 (72.08, 202) |
| **Bahrain** | 12.31 (10.25, 14.72) | 3.93 (3.27, 4.72) | 22.9 (17.66, 29.31) | 1.48 (1.16, 1.85) | -62.48 (-71.91, -50.46) |
| **Bangladesh** | 2256.42 (1474.58, 3419.36) | 2.76 (1.72, 4.17) | 4631.5 (2740.57, 7335.94) | 3.22 (1.89, 5.14) | 16.92 (-30.84, 79.01) |
| **Barbados** | 13.67 (12.64, 14.74) | 5.77 (5.33, 6.26) | 135.64 (102.23, 172.48) | 32.28 (24.18, 41.33) | 459.17 (318.42, 609.28) |
| **Belarus** | 279.42 (222.6, 405.5) | 2.37 (1.91, 3.44) | 775.25 (581.36, 1053.89) | 5.46 (4.09, 7.43) | 130.6 (57.04, 221.3) |
| **Belgium** | 7088.78 (6723.92, 7436.52) | 54.09 (51.09, 57.22) | 7108.35 (6368.33, 7863.53) | 38.59 (34.65, 42.65) | -28.66 (-36.54, -20.51) |
| **Belize** | 6.52 (5.65, 7.52) | 4.95 (4.43, 5.52) | 30.69 (25.22, 36.5) | 8.98 (7.39, 10.64) | 81.45 (43.67, 121.69) |
| **Benin** | 70.8 (51.43, 100.86) | 2.27 (1.59, 3.22) | 117.88 (81.04, 162.24) | 1.36 (0.93, 1.88) | -40.44 (-62.59, -4.63) |
| **Bermuda** | 4.59 (3.88, 5.82) | 7.45 (6.26, 9.42) | 17.04 (13.52, 21.23) | 16.38 (12.97, 20.31) | 119.96 (60.06, 195.27) |
| **Bhutan** | 9.18 (4.29, 15.49) | 2.06 (1.01, 3.68) | 19.1 (9.67, 33.66) | 2.99 (1.5, 5.24) | 44.7 (-13.15, 147.15) |
| **Bolivia (Plurinational State of)** | 368.32 (271.58, 498.21) | 8.21 (6.17, 11.19) | 1060.9 (709.36, 1553.73) | 10.87 (7.27, 15.87) | 32.42 (-13.24, 96.3) |
| **Bosnia and Herzegovina** | 621.28 (522.3, 735.37) | 13.58 (11.4, 16.11) | 53.36 (38.93, 72.66) | 1.13 (0.84, 1.5) | -91.71 (-94.15, -88.39) |
| **Botswana** | 17.94 (11.55, 26.6) | 2.25 (1.34, 3.48) | 41.26 (26.82, 60.02) | 2.2 (1.43, 3.2) | -1.99 (-42.75, 69.59) |
| **Brazil** | 13486.71 (12664.87, 14539.16) | 11.19 (10.64, 11.91) | 42611.6 (38980.29, 45596.34) | 18.14 (16.59, 19.45) | 62.14 (40.47, 78.6) |
| **Brunei Darussalam** | 5.77 (3.86, 9.88) | 2.63 (1.87, 4.24) | 21.69 (15.9, 31.41) | 5.88 (4.17, 8.89) | 123.18 (21.15, 288.81) |
| **Bulgaria** | 855.13 (793.95, 926.36) | 8.75 (8.07, 9.59) | 281.74 (217.02, 360.58) | 2.8 (2.16, 3.58) | -67.98 (-75.63, -58.19) |
| **Burkina Faso** | 132.77 (87.55, 199.02) | 2.04 (1.23, 3.19) | 217.76 (154.64, 296.31) | 1.31 (0.87, 1.87) | -36.06 (-56.2, -3.59) |
| **Burundi** | 34.24 (23.94, 49) | 0.66 (0.45, 1.02) | 68.89 (47.74, 96.5) | 0.59 (0.38, 0.9) | -11.56 (-39.29, 25.7) |
| **Cabo Verde** | 2.63 (2.15, 3.19) | 0.96 (0.76, 1.22) | 8.26 (6.44, 10.57) | 1.69 (1.31, 2.17) | 76.17 (21.66, 152.98) |
| **Cambodia** | 181.65 (134.43, 249.52) | 2.47 (1.81, 3.31) | 342.23 (230.65, 469.7) | 2.37 (1.58, 3.29) | -3.82 (-33.7, 37.89) |
| **Cameroon** | 210.56 (142.55, 289.05) | 3.33 (2.19, 4.7) | 426.09 (271.15, 676.93) | 2.36 (1.44, 3.94) | -29.13 (-61.25, 19.3) |
| **Canada** | 11358.29 (10857.67, 11853.89) | 38.05 (36.22, 39.96) | 26379.89 (24165.54, 28673.78) | 44.46 (40.89, 48.11) | 16.83 (6.48, 27.43) |
| **Central African Republic** | 17.29 (11.86, 26.43) | 0.75 (0.49, 1.24) | 25.7 (18.38, 36.09) | 0.57 (0.38, 0.85) | -23.65 (-48.14, 12.57) |
| **Chad** | 76.4 (49.19, 121.58) | 1.9 (1.13, 3.25) | 131.19 (93.84, 172.96) | 1.22 (0.83, 1.72) | -35.57 (-57.95, -0.53) |
| **Chile** | 1555.71 (1440.39, 1693.25) | 13.49 (12.53, 14.6) | 5326.31 (4755.15, 5868.45) | 23.78 (21.17, 26.34) | 76.3 (53.07, 99.81) |
| **China** | 141723.22 (124717.21, 160210.84) | 12.61 (11.14, 14.19) | 97908.4 (83545.05, 113578.45) | 6.11 (5.32, 6.98) | -51.58 (-60.02, -41.33) |
| **Colombia** | 1744.23 (1613.4, 1897.64) | 7.05 (6.61, 7.53) | 7039.85 (5213.6, 9194.16) | 13.85 (10.26, 18) | 96.35 (44.15, 158.41) |
| **Comoros** | 2.79 (0.99, 4.2) | 0.65 (0.24, 1.03) | 4.73 (2.93, 6.58) | 0.7 (0.44, 0.99) | 8.18 (-32.65, 183.02) |
| **Congo** | 18.05 (12.99, 26.13) | 0.95 (0.67, 1.35) | 37.13 (24.57, 52.57) | 0.88 (0.58, 1.28) | -7.38 (-43.69, 44.36) |
| **Cook Islands** | 1.23 (0.91, 1.58) | 8.53 (6.31, 10.88) | 0.93 (0.66, 1.28) | 3.76 (2.68, 5.17) | -55.87 (-69.85, -34.85) |
| **Costa Rica** | 120.71 (110.36, 134.28) | 5.46 (5.04, 5.93) | 1433.76 (1063.93, 1857.45) | 27.48 (20.49, 35.76) | 403.46 (267.38, 562.05) |
| **Croatia** | 965.64 (882.44, 1056.61) | 18.52 (16.75, 20.34) | 1175.23 (889.92, 1510.9) | 19.82 (14.99, 25.47) | 7.04 (-21.58, 40.66) |
| **Cuba** | 584.69 (544.71, 631.61) | 5.79 (5.39, 6.26) | 3115.42 (2478.77, 3853.78) | 19.43 (15.56, 23.85) | 235.61 (165.09, 320.37) |
| **Cyprus** | 130.75 (104.94, 165.52) | 15.64 (12.64, 19.72) | 372.52 (306.21, 450.81) | 20.16 (16.55, 24.37) | 28.91 (-6.63, 79.25) |
| **Czechia** | 1850.9 (1719.53, 2000.83) | 17.09 (15.59, 18.98) | 2415.67 (1948.95, 2959.16) | 15.52 (12.42, 19.4) | -9.19 (-28.51, 14.65) |
| **Côte d'Ivoire** | 186.45 (126.11, 293.86) | 2.67 (1.76, 4.2) | 260.09 (172.48, 356.23) | 1.48 (0.99, 2.07) | -44.65 (-67.75, -9.07) |
| **Democratic People's Republic of Korea** | 2261.91 (1301.48, 4043.5) | 8.77 (5.44, 14.14) | 1547.21 (989.84, 2424.4) | 5.22 (3.3, 7.95) | -40.43 (-67.82, 2.98) |
| **Democratic Republic of the Congo** | 256.11 (172.3, 402.63) | 0.73 (0.49, 1.27) | 453.08 (329.53, 637.79) | 0.64 (0.42, 0.95) | -12.71 (-41.92, 26.94) |
| **Denmark** | 2378.23 (2195.28, 2574.6) | 32.67 (30.29, 35.18) | 3559.43 (3191.31, 3950.78) | 34.18 (30.78, 37.72) | 4.61 (-8.85, 19.16) |
| **Djibouti** | 2.88 (1.87, 4.42) | 0.69 (0.41, 1.11) | 8.82 (5.53, 12.85) | 0.79 (0.47, 1.21) | 14.56 (-27.27, 82.82) |
| **Dominica** | 4.27 (3.61, 5.01) | 6.66 (5.59, 7.94) | 19.03 (13.66, 25.08) | 25.34 (18.08, 33.77) | 280.24 (157.77, 441.33) |
| **Dominican Republic** | 393.64 (309.18, 498.97) | 6.71 (5.47, 8.06) | 1260.43 (760.67, 1999.16) | 12.44 (7.56, 19.63) | 85.45 (10.76, 199.67) |
| **Ecuador** | 351.03 (317.75, 386.52) | 4.97 (4.52, 5.45) | 1796.99 (1355.06, 2368.19) | 11.25 (8.5, 14.82) | 126.39 (68.54, 204.03) |
| **Egypt** | 1786.39 (1486.35, 2175.64) | 4.23 (3.52, 5.04) | 6029.22 (3334.33, 9617.57) | 7.47 (4.13, 11.92) | 76.38 (-5.38, 195.47) |
| **El Salvador** | 162.43 (142.84, 184.34) | 4.15 (3.69, 4.67) | 199.98 (142.1, 269.58) | 3.43 (2.43, 4.63) | -17.28 (-43.27, 14.94) |
| **Equatorial Guinea** | 2.5 (1.65, 4.16) | 0.65 (0.4, 1.11) | 7.82 (4.54, 12.55) | 0.86 (0.5, 1.41) | 32.9 (-30.38, 151.63) |
| **Eritrea** | 15.76 (10.93, 22.97) | 0.64 (0.41, 1.07) | 44.77 (31.64, 61.94) | 0.76 (0.51, 1.12) | 18.84 (-19.99, 79.02) |
| **Estonia** | 87.53 (77.88, 105.49) | 4.52 (4.04, 5.43) | 55.21 (42.65, 70.51) | 2.66 (2.05, 3.42) | -41.07 (-57.38, -22.42) |
| **Eswatini** | 10.58 (7.38, 14.84) | 2.31 (1.52, 3.41) | 17.37 (11.65, 25.26) | 2.15 (1.42, 3.14) | -6.85 (-47.04, 73.56) |
| **Ethiopia** | 361.35 (263.96, 544.23) | 0.78 (0.51, 1.35) | 603.9 (474.33, 764.56) | 0.62 (0.43, 0.87) | -20.3 (-43.25, 9.76) |
| **Fiji** | 13.1 (9.75, 17.08) | 2.61 (1.9, 3.46) | 23.37 (16.36, 32.07) | 2.61 (1.83, 3.55) | 0.08 (-38.53, 60.99) |
| **Finland** | 3077.82 (2901.61, 3274.52) | 47.29 (44.49, 50.31) | 5112.34 (4419.04, 5811.15) | 50.56 (44.47, 56.96) | 6.91 (-7.6, 23.8) |
| **France** | 25438.68 (24332.37, 26655.93) | 38.02 (35.8, 40.34) | 41420.83 (36643, 46057.95) | 38.46 (34.1, 42.71) | 1.15 (-11.27, 14.56) |
| **Gabon** | 8.52 (5.66, 14.89) | 1 (0.65, 1.72) | 14.08 (9.21, 20.61) | 0.96 (0.62, 1.43) | -4.16 (-46.83, 52.39) |
| **Gambia** | 11.48 (7.13, 17.31) | 1.96 (1.16, 3.1) | 22.63 (16.33, 29.27) | 1.53 (1.07, 2.05) | -21.93 (-55.36, 34.85) |
| **Georgia** | 160 (122.87, 252.99) | 2.55 (1.97, 3.98) | 175.68 (145, 212.59) | 3.68 (3, 4.47) | 44.53 (-7.79, 103.67) |
| **Germany** | 34199.91 (32804.5, 35624.11) | 34.86 (33.06, 36.99) | 48544.04 (44930.39, 52170.85) | 32.93 (30.71, 35.29) | -5.55 (-14.09, 2.91) |
| **Ghana** | 213.93 (157.5, 282.85) | 2.32 (1.67, 3.14) | 586.26 (422.85, 793.62) | 2.67 (1.9, 3.68) | 14.88 (-29.21, 87.41) |
| **Greece** | 1052.89 (961.82, 1191.35) | 9.4 (8.22, 11.99) | 4700.68 (4104.12, 5336.68) | 32.46 (27.56, 38.38) | 245.15 (183.54, 314.63) |
| **Greenland** | 4.76 (3.74, 6.05) | 10.84 (8.76, 13.38) | 7.19 (5.43, 8.98) | 10.08 (7.58, 12.89) | -7.01 (-33.26, 29.74) |
| **Grenada** | 3.64 (3.2, 4.11) | 5.42 (4.81, 6.07) | 20.38 (17.32, 23.26) | 17.88 (15.29, 20.45) | 230.04 (169.74, 288.52) |
| **Guam** | 21.3 (16.58, 26.82) | 23.63 (18.33, 29.54) | 4.01 (2.99, 5.25) | 2.02 (1.52, 2.63) | -91.44 (-94.09, -87.65) |
| **Guatemala** | 282.16 (246.96, 323.56) | 4.85 (4.25, 5.53) | 495.08 (378.13, 640.92) | 4.07 (3.12, 5.28) | -16.04 (-37.02, 13.31) |
| **Guinea** | 104.03 (70.17, 152.69) | 2.25 (1.43, 3.41) | 130.01 (94.08, 175.91) | 1.47 (1.04, 2.07) | -34.77 (-57.61, 3.86) |
| **Guinea-Bissau** | 18.19 (12.64, 26.25) | 3.07 (2.06, 4.47) | 20.44 (14.85, 27.53) | 1.75 (1.23, 2.41) | -42.94 (-63.88, -9.07) |
| **Guyana** | 26.72 (22.53, 31.06) | 5.01 (4.24, 5.77) | 106.63 (79.82, 138.98) | 14.71 (11.06, 19.09) | 193.77 (114.39, 293.64) |
| **Haiti** | 678.91 (372.84, 1212.32) | 10.02 (6.95, 15.14) | 1342.23 (848.63, 2157.83) | 12.62 (8.29, 18.47) | 25.89 (-25.56, 106.26) |
| **Honduras** | 482.65 (346.74, 681.79) | 11.72 (8.98, 15.03) | 1315.28 (795.76, 1950.21) | 17.84 (10.71, 26.59) | 52.13 (-8.78, 135.51) |
| **Hungary** | 2376.26 (2213.27, 2555.17) | 22.91 (20.72, 25.31) | 2649.64 (2132.99, 3277.04) | 20.26 (16.17, 25.18) | -11.59 (-31.83, 10.98) |
| **Iceland** | 89.91 (83.18, 96.84) | 33.98 (31.42, 36.66) | 170.81 (148.29, 194.23) | 36.1 (31.04, 41.69) | 6.23 (-9.71, 24.56) |
| **India** | 18519.09 (13958.17, 23588.62) | 2.76 (2.01, 3.66) | 52614.49 (40778.65, 65366.58) | 4.15 (3.22, 5.18) | 50.6 (16.33, 91.74) |
| **Indonesia** | 4049.48 (3113.62, 5420.12) | 2.79 (2.14, 3.66) | 7953.74 (5319.98, 11153.8) | 3.08 (2.07, 4.29) | 10.32 (-15.82, 39.92) |
| **Iran (Islamic Republic of)** | 1129.47 (861.07, 1653.03) | 2.71 (2.06, 3.85) | 3291.44 (2874.81, 3757.86) | 4.1 (3.57, 4.69) | 51.5 (0.05, 113.12) |
| **Iraq** | 141.81 (107.24, 196.34) | 1.14 (0.8, 1.68) | 422.27 (305.73, 572.55) | 1.3 (0.93, 1.77) | 14.2 (-35.56, 91.02) |
| **Ireland** | 1541.14 (1446.35, 1646.64) | 41.13 (38.51, 44.13) | 3787.76 (3348.65, 4190.23) | 56.43 (49.79, 62.46) | 37.19 (18.6, 55.4) |
| **Israel** | 1373.95 (1238.93, 1542.16) | 28.32 (25.59, 31.71) | 2716.46 (2455.1, 2974.15) | 26.09 (23.57, 28.64) | -7.89 (-20.77, 6.68) |
| **Italy** | 20005.29 (19463.72, 20650.99) | 31.89 (30.35, 34.21) | 37221.42 (33240.6, 40535.44) | 35.95 (32.33, 39.17) | 12.73 (0.9, 25.29) |
| **Jamaica** | 97.63 (88.05, 108.56) | 5.25 (4.81, 5.74) | 366.06 (267.2, 479.68) | 13.29 (9.54, 17.58) | 153.08 (83.86, 233.57) |
| **Japan** | 26040.81 (25336.03, 26584.26) | 16.19 (15.78, 16.52) | 47527.77 (42497.17, 51493.6) | 17.38 (15.87, 18.61) | 7.32 (-0.97, 14.63) |
| **Jordan** | 45.97 (36.25, 59.45) | 1.82 (1.38, 2.38) | 272.9 (201.83, 379) | 2.65 (1.97, 3.62) | 45.72 (-6.63, 129.69) |
| **Kazakhstan** | 61.68 (49.03, 78.58) | 0.44 (0.35, 0.57) | 147.77 (124.5, 172.89) | 0.73 (0.62, 0.86) | 66.18 (21.79, 120.95) |
| **Kenya** | 126.02 (99.76, 157.36) | 0.64 (0.46, 0.9) | 308.71 (242.4, 393.9) | 0.76 (0.58, 1) | 19.63 (-12.69, 62.88) |
| **Kiribati** | 6.87 (5.2, 8.93) | 15.24 (11.43, 19.96) | 12.05 (8.34, 17.58) | 14.8 (10.15, 21.72) | -2.9 (-38.58, 58.18) |
| **Kuwait** | 231.82 (182.44, 306.58) | 14.8 (11.91, 19.03) | 177.54 (124.42, 226.99) | 5.27 (3.69, 6.77) | -64.42 (-75.17, -50.37) |
| **Kyrgyzstan** | 68.96 (54.49, 108.66) | 2.02 (1.59, 3.21) | 171.7 (149.28, 197.69) | 2.8 (2.43, 3.22) | 38.99 (-14.54, 91.97) |
| **Lao People's Democratic Republic** | 90.39 (62.56, 138.24) | 2.74 (1.93, 4.05) | 137.88 (89.15, 199.91) | 2.37 (1.53, 3.43) | -13.58 (-44.58, 32.21) |
| **Latvia** | 60.77 (53.25, 79.59) | 1.79 (1.58, 2.33) | 235.16 (191.58, 286.65) | 7.47 (6.13, 9.17) | 317.07 (202.88, 436.49) |
| **Lebanon** | 70.21 (47.7, 111.89) | 2.56 (1.7, 4.13) | 137.97 (80.73, 227.13) | 2.68 (1.57, 4.45) | 4.86 (-40.21, 68.05) |
| **Lesotho** | 22.5 (14.45, 35.29) | 1.77 (1.09, 2.9) | 33.78 (22.63, 48.03) | 2.11 (1.4, 3.05) | 18.87 (-33.26, 115.1) |
| **Liberia** | 36.67 (26.26, 49.62) | 2.46 (1.69, 3.41) | 44.78 (29.87, 64.67) | 1.37 (0.88, 2.1) | -44.58 (-66.88, -8.88) |
| **Libya** | 57.91 (42.1, 84.94) | 2.15 (1.49, 3.2) | 208.51 (114.83, 326.99) | 3.22 (1.78, 4.91) | 50.12 (-29.26, 183.29) |
| **Lithuania** | 259.01 (225.3, 328.86) | 6.25 (5.44, 7.86) | 904.69 (731.04, 1111.85) | 20.55 (16.84, 25.01) | 228.58 (145.24, 330.61) |
| **Luxembourg** | 152.79 (141.06, 164.06) | 33.3 (30.53, 36.08) | 234.83 (201.48, 273.09) | 27.5 (23.14, 32.76) | -17.42 (-31.59, -0.94) |
| **Madagascar** | 89.09 (65.4, 128.2) | 0.78 (0.54, 1.11) | 147.37 (110.39, 183.01) | 0.64 (0.45, 0.85) | -18.31 (-43.08, 14.16) |
| **Malawi** | 65.3 (45.82, 95.5) | 0.7 (0.47, 1.06) | 117.52 (86.13, 156.4) | 0.7 (0.47, 0.97) | -0.92 (-30.71, 44.14) |
| **Malaysia** | 208.96 (170.34, 254.83) | 1.67 (1.33, 2.1) | 443.01 (321.25, 605.35) | 1.45 (1.05, 1.98) | -13.4 (-42.01, 28.62) |
| **Maldives** | 8.91 (4.61, 17.73) | 4.5 (2.88, 7.53) | 22.84 (15.99, 33.41) | 5.7 (3.97, 8.39) | 26.62 (-35.04, 135.9) |
| **Mali** | 141.39 (94.1, 208.21) | 2.27 (1.41, 3.54) | 199.64 (137.43, 275.75) | 1.3 (0.84, 1.89) | -42.77 (-62.41, -15.2) |
| **Malta** | 110.1 (101.47, 119.29) | 27.73 (25.23, 30.41) | 230.62 (195.6, 267.43) | 33.79 (28.03, 40.37) | 21.86 (-0.7, 49.16) |
| **Marshall Islands** | 1.39 (1.02, 1.9) | 7.04 (4.99, 9.62) | 1.97 (1.38, 2.7) | 4.38 (3.08, 6.03) | -37.77 (-60.82, -1.76) |
| **Mauritania** | 41.77 (31.39, 54.64) | 3.29 (2.4, 4.4) | 43.35 (27.35, 62.57) | 1.56 (0.97, 2.25) | -52.64 (-70.93, -25.69) |
| **Mauritius** | 55.86 (51.39, 61.09) | 6.14 (5.67, 6.66) | 122.45 (96.69, 153.97) | 7.65 (5.95, 9.69) | 24.74 (-2.84, 58.8) |
| **Mexico** | 4847.08 (4556.87, 5163.94) | 7.46 (7.18, 7.76) | 15601.57 (12944.41, 18181.15) | 12.58 (10.48, 14.61) | 68.54 (38.07, 97.09) |
| **Micronesia (Federated States of)** | 3.91 (2.74, 5.33) | 6.98 (4.78, 9.6) | 4.06 (2.62, 5.85) | 4.45 (2.89, 6.36) | -36.26 (-62.78, 5.94) |
| **Monaco** | 3.89 (2.88, 5.16) | 9.25 (6.83, 12.66) | 6.36 (4.53, 8.6) | 9.05 (6.53, 12.14) | -2.2 (-33.5, 42.72) |
| **Mongolia** | 133.81 (84.91, 226.64) | 6.47 (4.7, 9.59) | 198.03 (136.78, 294.15) | 6.29 (4.43, 9.06) | -2.8 (-43.03, 58.23) |
| **Montenegro** | 10.97 (9.2, 12.98) | 1.7 (1.43, 2) | 14.88 (11.75, 18.56) | 1.72 (1.37, 2.13) | 1.28 (-24.06, 34.72) |
| **Morocco** | 278.82 (210.32, 371.33) | 1.52 (1.11, 2.14) | 719.64 (504.52, 991.22) | 1.98 (1.39, 2.7) | 30.09 (-17.51, 90.34) |
| **Mozambique** | 81.66 (55.64, 128.16) | 0.64 (0.4, 1.1) | 211.89 (151.46, 279.89) | 0.75 (0.5, 1.1) | 16.91 (-20.35, 74.67) |
| **Myanmar** | 972.79 (680.1, 1400.96) | 2.91 (2.06, 4.1) | 1417.53 (949.03, 2026.24) | 2.65 (1.77, 3.76) | -8.88 (-42.48, 43.07) |
| **Namibia** | 19.59 (13.03, 27.12) | 2.19 (1.45, 3.08) | 33.98 (23.67, 47.45) | 1.87 (1.32, 2.65) | -14.43 (-46.34, 44.47) |
| **Nauru** | 0.47 (0.31, 0.74) | 8.7 (5.77, 13.99) | 0.31 (0.22, 0.45) | 4.86 (3.32, 6.84) | -44.17 (-65.05, -16.32) |
| **Nepal** | 334.28 (206.65, 527.5) | 2.13 (1.24, 3.66) | 787.08 (470.22, 1290.89) | 3.04 (1.79, 5.1) | 43.23 (-8.64, 115.07) |
| **Netherlands** | 7503.76 (7112.8, 7906.55) | 44.55 (41.77, 47.92) | 13128.16 (11943.8, 14356.55) | 46.43 (42.03, 50.87) | 4.22 (-7.37, 16.28) |
| **New Zealand** | 1684.91 (1588.17, 1781.3) | 45.2 (42.57, 47.77) | 3503.84 (3204.11, 3792.44) | 51.48 (47.35, 55.6) | 13.89 (3.67, 25.91) |
| **Nicaragua** | 81.6 (68.08, 98.68) | 3.47 (2.87, 4.21) | 173.17 (128.53, 228.2) | 3.4 (2.52, 4.5) | -2.15 (-33.05, 36.73) |
| **Niger** | 100.54 (63.2, 160.64) | 2 (1.11, 3.45) | 165.05 (111.99, 238.56) | 1.01 (0.6, 1.65) | -49.44 (-64.59, -21.79) |
| **Nigeria** | 1317.93 (953.55, 1749.49) | 2.11 (1.44, 2.85) | 2175.49 (1529.49, 3034.68) | 1.57 (1.07, 2.22) | -25.82 (-54.94, 17.73) |
| **Niue** | 0.19 (0.13, 0.26) | 9.02 (6.22, 12.83) | 0.12 (0.08, 0.16) | 5.32 (3.83, 7.23) | -40.99 (-63.1, -2.34) |
| **North Macedonia** | 235.59 (178.99, 326.09) | 12.57 (9.34, 17.99) | 59.22 (43.67, 76.47) | 2.01 (1.51, 2.59) | -84 (-89.69, -76.22) |
| **Northern Mariana Islands** | 1.18 (0.88, 1.62) | 4.18 (3.17, 5.6) | 1.18 (0.88, 1.55) | 1.79 (1.37, 2.31) | -57.33 (-71.67, -38.53) |
| **Norway** | 2312.87 (2233.7, 2384.51) | 41.66 (40.3, 42.97) | 3162.5 (2881.5, 3440.25) | 37.89 (34.65, 40.96) | -9.05 (-17.7, -0.14) |
| **Oman** | 23.78 (16.65, 44.83) | 2.21 (1.43, 4.48) | 75.76 (45.56, 105.87) | 2.66 (1.42, 3.88) | 20.71 (-36.93, 109.91) |
| **Pakistan** | 2306.74 (1493.46, 3465.39) | 2.65 (1.67, 3.94) | 7684.4 (5890.28, 10180.22) | 4.56 (3.48, 5.97) | 71.9 (17.59, 161.26) |
| **Palau** | 0.15 (0.1, 0.2) | 1.23 (0.86, 1.69) | 0.33 (0.24, 0.47) | 1.32 (0.96, 1.8) | 7.31 (-32.88, 71.17) |
| **Palestine** | 17.37 (12.39, 25.84) | 1.05 (0.7, 1.59) | 58.87 (47.05, 73.51) | 1.53 (1.21, 1.9) | 45.49 (-11.55, 143.88) |
| **Panama** | 112.94 (101.46, 125.04) | 6.11 (5.55, 6.65) | 341.18 (254.05, 446.74) | 8.21 (6.12, 10.75) | 34.44 (-1.05, 76.81) |
| **Papua New Guinea** | 77.73 (40.91, 125.61) | 3.01 (1.46, 5.1) | 129.6 (74.62, 212.54) | 1.89 (0.98, 3.21) | -37.15 (-57.6, -5.49) |
| **Paraguay** | 123.72 (101.13, 149.06) | 4.19 (3.44, 5.04) | 741.87 (519.37, 1049.92) | 12.24 (8.57, 17.27) | 192.39 (91.05, 343.94) |
| **Peru** | 773.96 (636.07, 946.84) | 4.87 (3.99, 5.97) | 1611.78 (1101.55, 2293.23) | 4.91 (3.36, 6.98) | 0.81 (-35.46, 53.28) |
| **Philippines** | 1025.43 (885.22, 1157.45) | 2.14 (1.78, 2.47) | 2391.4 (1983.45, 2869.69) | 2.42 (2, 2.91) | 12.91 (-14.06, 47.06) |
| **Poland** | 4187.79 (4007.49, 4461.68) | 11.64 (11.01, 12.66) | 11563.75 (9699, 13647.02) | 21.95 (18.36, 25.75) | 88.53 (57.47, 123.44) |
| **Portugal** | 2171.67 (2019.04, 2349.57) | 21.67 (19.5, 24.49) | 5835.37 (5196.56, 6462.01) | 34.83 (30.58, 39.22) | 60.74 (34.3, 89.71) |
| **Puerto Rico** | 222.58 (207.49, 239.33) | 6.34 (5.89, 6.83) | 1000.66 (750.95, 1306.05) | 19.14 (14.32, 25.17) | 202.06 (125.37, 297.27) |
| **Qatar** | 4.22 (3.11, 5.67) | 1.66 (1.18, 2.33) | 30.03 (21.34, 41.47) | 1.53 (1.08, 2.09) | -7.87 (-45.26, 50.35) |
| **Republic of Korea** | 7995.21 (6434.21, 10002.12) | 23.18 (18.44, 29.23) | 2248.22 (1940.31, 2564.64) | 2.64 (2.31, 2.98) | -88.61 (-91.32, -85.27) |
| **Republic of Moldova** | 382.68 (339.21, 448.89) | 8.66 (7.63, 10.2) | 241.08 (206.71, 279.35) | 5.46 (4.7, 6.29) | -36.98 (-49.28, -22.34) |
| **Romania** | 3682.15 (3281.13, 4171.35) | 18.59 (16.09, 21.65) | 1943.15 (1589.57, 2349.68) | 8.66 (7.14, 10.46) | -53.43 (-63.61, -41.54) |
| **Russian Federation** | 6542 (5701.36, 8807.64) | 4 (3.52, 5.32) | 21570.06 (18483.07, 24678.28) | 10.45 (9.04, 11.88) | 161.25 (86.25, 224.87) |
| **Rwanda** | 51.4 (36.42, 74.61) | 0.84 (0.56, 1.28) | 89.72 (64.32, 123.43) | 0.77 (0.52, 1.15) | -7.97 (-37.03, 31.39) |
| **Saint Kitts and Nevis** | 2 (1.81, 2.21) | 5.91 (5.34, 6.54) | 14.56 (10.54, 18.41) | 20.33 (14.75, 25.6) | 243.98 (147.08, 345.08) |
| **Saint Lucia** | 6.05 (5.5, 6.72) | 5.89 (5.43, 6.44) | 43.95 (35.38, 53.83) | 21.6 (17.34, 26.75) | 266.55 (188.05, 362.05) |
| **Saint Vincent and the Grenadines** | 5.31 (4.76, 6) | 6.42 (5.8, 7.14) | 21.12 (17.61, 25.34) | 16.3 (13.48, 19.75) | 154.09 (104.45, 214.38) |
| **Samoa** | 7.34 (4.58, 11.14) | 7.3 (4.46, 11.17) | 6.2 (4.13, 8.79) | 3.67 (2.44, 5.21) | -49.75 (-67.52, -20.82) |
| **San Marino** | 0.89 (0.71, 1.14) | 3.3 (2.63, 4.16) | 1.5 (0.89, 2.34) | 3.17 (1.9, 4.9) | -3.88 (-44.57, 60.38) |
| **Sao Tome and Principe** | 9.23 (6.26, 13.39) | 9 (6.35, 12.07) | 22.86 (13.03, 33.15) | 15.67 (8.98, 22.87) | 74.1 (-2.7, 181.88) |
| **Saudi Arabia** | 214.3 (125.98, 365.32) | 2.09 (1.09, 3.6) | 744.09 (494.9, 1102.19) | 2.57 (1.78, 3.65) | 22.45 (-40.31, 166.42) |
| **Senegal** | 123.36 (81.68, 181.86) | 2.53 (1.6, 3.84) | 169.72 (115.75, 229.12) | 1.54 (1.05, 2.15) | -39.08 (-62.93, -2.65) |
| **Serbia** | 247.64 (207.68, 297.69) | 2.16 (1.84, 2.56) | 259.5 (194.42, 335.02) | 1.98 (1.5, 2.54) | -8.51 (-35.53, 24.36) |
| **Seychelles** | 5.16 (4.21, 6.33) | 8.6 (6.99, 10.54) | 12.57 (7.69, 19.57) | 10.74 (6.65, 16.27) | 24.9 (-21.18, 87.93) |
| **Sierra Leone** | 56.57 (37.88, 81.04) | 2.06 (1.34, 2.98) | 79.24 (55.32, 109.73) | 1.34 (0.92, 1.9) | -35.1 (-59.39, 1.69) |
| **Singapore** | 350.18 (315.93, 396.3) | 13.83 (12.41, 15.67) | 783.32 (663.42, 905.64) | 11.78 (9.78, 13.93) | -14.86 (-32.31, 4.55) |
| **Slovakia** | 201.12 (175.67, 232.63) | 3.56 (3.12, 4.13) | 409.98 (307.49, 538.94) | 4.98 (3.74, 6.48) | 39.85 (3.02, 89.79) |
| **Slovenia** | 359.82 (285.2, 452.75) | 18.72 (15.2, 23.16) | 31.89 (23.73, 43.14) | 0.93 (0.69, 1.26) | -95.02 (-96.59, -92.99) |
| **Solomon Islands** | 9.75 (6.15, 15.35) | 5.27 (3.19, 8.5) | 13.91 (9.64, 19.01) | 3.25 (2.18, 4.53) | -38.34 (-59.58, -1.69) |
| **Somalia** | 33.43 (22.29, 49.88) | 0.54 (0.33, 0.93) | 98.03 (68.03, 135.4) | 0.49 (0.3, 0.77) | -8.02 (-33.31, 29.72) |
| **South Africa** | 491.51 (434.33, 551.11) | 1.77 (1.55, 2.01) | 638.01 (571.77, 705.48) | 1.21 (1.09, 1.34) | -31.42 (-41.18, -18.63) |
| **South Sudan** | 39.47 (23.92, 97.49) | 0.71 (0.4, 1.44) | 49.9 (35.27, 73.08) | 0.56 (0.36, 0.86) | -21.82 (-53.55, 19.44) |
| **Spain** | 12509.71 (11962.8, 13081.28) | 29.01 (27.35, 31.19) | 25246.4 (22448.36, 27998.8) | 33.82 (30.18, 37.34) | 16.58 (3.12, 30.88) |
| **Sri Lanka** | 1303.09 (1029.53, 1694.8) | 9.12 (7.33, 11.5) | 884.09 (599.85, 1250.59) | 3.6 (2.45, 5.18) | -60.5 (-74.54, -40.7) |
| **Sudan** | 193.78 (133.41, 296.03) | 1.41 (0.93, 2.24) | 472.42 (327.61, 673.5) | 1.7 (1.13, 2.52) | 20.47 (-28.03, 97.14) |
| **Suriname** | 18.87 (15.35, 22.38) | 5.95 (4.94, 6.99) | 86.19 (63.54, 114.59) | 14.44 (10.61, 19.34) | 142.66 (73.77, 237.12) |
| **Sweden** | 4190.78 (3982.95, 4397.74) | 32.64 (31.08, 34.09) | 7115.09 (6531.58, 7644.48) | 40.06 (37.19, 42.82) | 22.75 (13.11, 32.8) |
| **Switzerland** | 3266.01 (3002.72, 3549.34) | 35.67 (32.78, 38.51) | 4612.54 (4196.01, 5059.3) | 31.24 (28.56, 34.11) | -12.41 (-23.17, -1.05) |
| **Syrian Arab Republic** | 144.8 (109.34, 193.39) | 1.77 (1.27, 2.49) | 345.83 (203.67, 565.51) | 2.32 (1.37, 3.81) | 31.06 (-26.28, 121.1) |
| **Taiwan (Province of China)** | 1839.33 (1726.06, 1964.37) | 9.69 (9.09, 10.37) | 4411.35 (3307.37, 5843.39) | 14.45 (11.04, 18.78) | 49.11 (11.83, 96.03) |
| **Tajikistan** | 39.74 (32.72, 49.65) | 0.94 (0.72, 1.26) | 96.13 (73.86, 122.93) | 1.22 (0.92, 1.59) | 29.17 (-12.81, 88.63) |
| **Thailand** | 749.58 (617.61, 921.91) | 1.63 (1.33, 2.02) | 1068.65 (748.99, 1489.11) | 1.12 (0.81, 1.52) | -31.01 (-52.49, 0.5) |
| **Timor-Leste** | 11.01 (6.68, 20.34) | 1.79 (1.05, 2.77) | 21.39 (11.44, 31.33) | 2.08 (1.1, 3.13) | 16.09 (-35.37, 82.28) |
| **Togo** | 49.99 (38.26, 65) | 2.43 (1.78, 3.29) | 87.02 (59.71, 119.95) | 1.54 (1.03, 2.18) | -36.56 (-61.42, 2.54) |
| **Tokelau** | 0.09 (0.06, 0.13) | 6.93 (4.49, 9.91) | 0.06 (0.04, 0.08) | 3.95 (2.66, 5.59) | -42.96 (-64.08, -10.81) |
| **Tonga** | 3.32 (2.12, 5.06) | 5.09 (3.19, 7.8) | 2.69 (1.69, 3.97) | 3.19 (1.98, 4.77) | -37.25 (-58.7, -4.33) |
| **Trinidad and Tobago** | 47.73 (43.97, 51.69) | 5 (4.61, 5.39) | 200.72 (142.1, 270.83) | 10.77 (7.65, 14.55) | 115.56 (51.35, 192.58) |
| **Tunisia** | 116.93 (89.94, 156.62) | 1.74 (1.3, 2.4) | 275.09 (181.07, 402.07) | 2.09 (1.38, 3.01) | 19.7 (-31.27, 104.03) |
| **Turkey** | 27152.55 (15222.9, 49533.42) | 41.61 (26.33, 71.02) | 22902.22 (17976.94, 28750.31) | 30.96 (23.82, 39.65) | -25.58 (-58.49, 21.23) |
| **Turkmenistan** | 108.14 (95.29, 123.03) | 3.96 (3.53, 4.42) | 326.54 (232.73, 441.49) | 6.79 (4.88, 9.09) | 71.44 (23.38, 122.54) |
| **Tuvalu** | 0.57 (0.38, 0.78) | 7.06 (4.69, 9.67) | 0.42 (0.28, 0.6) | 3.69 (2.45, 5.34) | -47.76 (-64.97, -19.36) |
| **Uganda** | 88.56 (55.57, 147.85) | 0.57 (0.32, 1.04) | 249.67 (180.52, 343.02) | 0.68 (0.45, 1.01) | 18.24 (-22.82, 87.44) |
| **Ukraine** | 2770.2 (2289.84, 3847.43) | 4.48 (3.76, 6.13) | 5253.88 (4326.36, 6274.83) | 9.32 (7.76, 11.01) | 107.88 (41.16, 168.91) |
| **United Arab Emirates** | 41.79 (27.03, 99.48) | 4.27 (2.34, 10.87) | 497.82 (196.09, 1004.61) | 5.33 (2.01, 10.56) | 24.9 (-47.6, 142.81) |
| **United Kingdom** | 31553.64 (30697.86, 32171.42) | 42.53 (41.18, 43.51) | 52160.14 (48595.45, 54916.57) | 48.34 (45.54, 50.77) | 13.67 (7.07, 20.8) |
| **United Republic of Tanzania** | 195.07 (138.95, 288.87) | 0.76 (0.52, 1.14) | 456.08 (347.47, 590.41) | 0.81 (0.58, 1.1) | 6.48 (-25.32, 47.89) |
| **United States of America** | 99154.13 (96371.51, 101695.94) | 35.44 (34.47, 36.44) | 216849.12 (209042.3, 222819.61) | 45.07 (43.55, 46.32) | 27.18 (22.37, 31.85) |
| **United States Virgin Islands** | 11.73 (9.31, 14.79) | 11.82 (9.37, 14.94) | 51 (36.48, 66.21) | 30.74 (22.63, 39.07) | 160.08 (77.71, 266.13) |
| **Uruguay** | 248.96 (233.38, 266.42) | 7.1 (6.65, 7.6) | 1306.95 (1190.82, 1418.79) | 27.98 (25.57, 30.36) | 293.84 (249.76, 335.11) |
| **Uzbekistan** | 89.4 (76.39, 122.06) | 0.67 (0.57, 0.94) | 313.79 (252.47, 381.82) | 1.08 (0.88, 1.3) | 60.97 (8.07, 108.33) |
| **Vanuatu** | 3.78 (2.14, 6.42) | 4.63 (2.56, 8.14) | 6.38 (4.04, 9.91) | 3.08 (1.91, 4.87) | -33.53 (-56.55, 6.73) |
| **Venezuela (Bolivarian Republic of)** | 707.12 (650.58, 770.69) | 5.25 (4.82, 5.66) | 3403.3 (2402.66, 4599.23) | 11.2 (7.92, 15.12) | 113.61 (48.58, 192.45) |
| **Viet Nam** | 1300.8 (898.78, 1870.52) | 2.64 (1.77, 3.84) | 3394.76 (2419.58, 4596.61) | 3.24 (2.34, 4.36) | 22.86 (-24.37, 98.54) |
| **Yemen** | 93.95 (58.98, 152.44) | 1.15 (0.67, 2.02) | 295.72 (205.94, 416.78) | 1.45 (1, 2.11) | 26.1 (-27.54, 120.64) |
| **Zambia** | 71.93 (52.21, 110.24) | 0.95 (0.7, 1.28) | 149.86 (113.79, 193.83) | 0.97 (0.71, 1.26) | 1.94 (-30.04, 44.58) |
| **Zimbabwe** | 268.05 (211.84, 337.96) | 4.71 (3.68, 5.88) | 613.7 (381.3, 958.16) | 6.4 (3.86, 10.13) | 36 (-23.16, 139.18) |
| **Death** | | | | | |
| **Afghanistan** | 5.92 (3.67, 10.19) | 0.07 (0.04, 0.12) | 11.41 (7.89, 16.61) | 0.06 (0.04, 0.1) | -8.74 (-41.44, 43.14) |
| **Albania** | 3.19 (2.87, 3.55) | 0.13 (0.12, 0.15) | 5.88 (4.2, 7.9) | 0.15 (0.11, 0.2) | 15.22 (-18.07, 55.86) |
| **Algeria** | 9.22 (6.58, 12.89) | 0.06 (0.04, 0.09) | 22.97 (17.02, 30.57) | 0.06 (0.04, 0.08) | -2.13 (-34.83, 49.57) |
| **American Samoa** | 0.07 (0.05, 0.1) | 0.3 (0.22, 0.38) | 0.04 (0.03, 0.06) | 0.08 (0.06, 0.11) | -71.96 (-80.88, -58.14) |
| **Andorra** | 0.94 (0.57, 1.48) | 1.72 (1.07, 2.66) | 2.85 (1.79, 4.27) | 2.1 (1.32, 3.14) | 22.23 (-24.55, 95.37) |
| **Angola** | 1.26 (0.79, 1.93) | 0.02 (0.01, 0.03) | 3.7 (2.64, 4.91) | 0.02 (0.01, 0.03) | -2.3 (-36.52, 53.32) |
| **Antigua and Barbuda** | 0.07 (0.06, 0.07) | 0.13 (0.12, 0.15) | 0.39 (0.31, 0.46) | 0.38 (0.31, 0.46) | 185.83 (127.34, 248.13) |
| **Argentina** | 52.24 (49.01, 55.84) | 0.16 (0.15, 0.17) | 361.61 (326.71, 390.42) | 0.69 (0.62, 0.74) | 333.71 (291.4, 375.46) |
| **Armenia** | 1.9 (1.61, 2.56) | 0.06 (0.05, 0.08) | 1.89 (1.53, 2.27) | 0.05 (0.04, 0.05) | -27.87 (-49.04, -7.48) |
| **Australia** | 311.87 (295.48, 327.45) | 1.61 (1.53, 1.7) | 866.59 (766.03, 961.78) | 2.14 (1.91, 2.37) | 32.56 (18.26, 47.07) |
| **Austria** | 75.35 (70.8, 79.93) | 0.72 (0.67, 0.76) | 183.8 (167.92, 199.64) | 1.15 (1.06, 1.25) | 60.53 (46.27, 76.52) |
| **Azerbaijan** | 2.22 (1.68, 2.93) | 0.04 (0.03, 0.05) | 5.68 (4.27, 7.61) | 0.05 (0.04, 0.07) | 32.2 (-8.49, 91.18) |
| **Bahamas** | 0.35 (0.31, 0.39) | 0.2 (0.18, 0.22) | 1.97 (1.53, 2.52) | 0.49 (0.38, 0.62) | 145.52 (84.85, 221.52) |
| **Bahrain** | 0.3 (0.25, 0.37) | 0.12 (0.1, 0.15) | 0.63 (0.48, 0.82) | 0.04 (0.03, 0.06) | -63.65 (-73.39, -51.14) |
| **Bangladesh** | 49.76 (31.45, 73.86) | 0.08 (0.05, 0.13) | 134.9 (75.39, 217.31) | 0.1 (0.05, 0.16) | 21.13 (-27.04, 84.24) |
| **Barbados** | 0.42 (0.39, 0.45) | 0.17 (0.15, 0.18) | 5.13 (3.91, 6.41) | 1.1 (0.83, 1.38) | 559.17 (394.3, 727.64) |
| **Belarus** | 8.48 (6.65, 12.56) | 0.07 (0.05, 0.1) | 27.13 (20.42, 36.71) | 0.18 (0.13, 0.24) | 164.19 (75.32, 275.63) |
| **Belgium** | 346.61 (323.49, 366.02) | 2.27 (2.12, 2.39) | 332.55 (293.7, 368.96) | 1.54 (1.37, 1.71) | -32.13 (-39.32, -24.59) |
| **Belize** | 0.16 (0.14, 0.18) | 0.15 (0.13, 0.16) | 0.84 (0.69, 0.98) | 0.27 (0.22, 0.32) | 84.52 (47.59, 125.56) |
| **Benin** | 1.71 (1.22, 2.39) | 0.07 (0.05, 0.1) | 2.62 (1.81, 3.61) | 0.04 (0.03, 0.05) | -44.19 (-65.35, -10.56) |
| **Bermuda** | 0.14 (0.12, 0.19) | 0.23 (0.19, 0.3) | 0.66 (0.53, 0.82) | 0.56 (0.44, 0.69) | 142.51 (75.15, 231.67) |
| **Bhutan** | 0.2 (0.1, 0.36) | 0.06 (0.03, 0.12) | 0.56 (0.27, 1) | 0.09 (0.05, 0.17) | 51.56 (-7.41, 144.18) |
| **Bolivia (Plurinational State of)** | 9.75 (7.39, 13.13) | 0.26 (0.19, 0.35) | 36.45 (24.75, 52.45) | 0.39 (0.27, 0.57) | 52.75 (2.19, 125.52) |
| **Bosnia and Herzegovina** | 13.66 (11.7, 15.84) | 0.3 (0.25, 0.34) | 1.71 (1.23, 2.37) | 0.03 (0.02, 0.04) | -89.27 (-92.48, -85.23) |
| **Botswana** | 0.48 (0.3, 0.75) | 0.07 (0.04, 0.11) | 1.11 (0.72, 1.64) | 0.07 (0.04, 0.1) | -5.79 (-45.29, 63.84) |
| **Brazil** | 336.21 (323.07, 353.75) | 0.33 (0.32, 0.35) | 1466.99 (1343.53, 1561.76) | 0.62 (0.57, 0.66) | 88.55 (67.02, 102.81) |
| **Brunei Darussalam** | 0.11 (0.08, 0.17) | 0.07 (0.05, 0.1) | 0.48 (0.37, 0.62) | 0.14 (0.1, 0.18) | 106.12 (18.21, 223.05) |
| **Bulgaria** | 23.65 (21.89, 25.55) | 0.21 (0.2, 0.23) | 9.31 (7.22, 11.9) | 0.08 (0.06, 0.1) | -61.86 (-71, -50.24) |
| **Burkina Faso** | 3.44 (2.09, 5.33) | 0.06 (0.04, 0.1) | 4.76 (3.22, 6.74) | 0.04 (0.02, 0.05) | -42.23 (-60.04, -12.16) |
| **Burundi** | 0.64 (0.44, 0.96) | 0.02 (0.01, 0.03) | 1.18 (0.77, 1.78) | 0.01 (0.01, 0.02) | -21.37 (-47.81, 16.33) |
| **Cabo Verde** | 0.06 (0.05, 0.08) | 0.03 (0.02, 0.03) | 0.23 (0.18, 0.3) | 0.05 (0.04, 0.07) | 93.61 (30.67, 188) |
| **Cambodia** | 4.28 (3.16, 5.74) | 0.07 (0.05, 0.1) | 9.91 (6.51, 13.89) | 0.07 (0.05, 0.1) | 1.22 (-30.81, 45.5) |
| **Cameroon** | 5.72 (3.74, 8.06) | 0.11 (0.07, 0.15) | 10.89 (6.68, 17.98) | 0.07 (0.04, 0.12) | -32.37 (-63.48, 16.91) |
| **Canada** | 444.98 (421.7, 466.84) | 1.4 (1.32, 1.46) | 1202.27 (1085.47, 1324.73) | 1.8 (1.64, 1.98) | 29.11 (17.08, 42.16) |
| **Central African Republic** | 0.36 (0.23, 0.59) | 0.02 (0.01, 0.04) | 0.51 (0.34, 0.76) | 0.01 (0.01, 0.02) | -28.74 (-53.87, 7.98) |
| **Chad** | 1.97 (1.18, 3.28) | 0.06 (0.03, 0.1) | 2.87 (1.99, 3.95) | 0.04 (0.02, 0.05) | -39.49 (-61.64, -4.27) |
| **Chile** | 43.86 (41.06, 46.83) | 0.41 (0.38, 0.44) | 195.62 (174.83, 215.3) | 0.83 (0.74, 0.91) | 103.15 (78.96, 127) |
| **China** | 2577.62 (2296.62, 2854.94) | 0.25 (0.22, 0.27) | 2878.08 (2442.78, 3369.98) | 0.16 (0.13, 0.18) | -37.47 (-48.53, -24.57) |
| **Colombia** | 44.61 (41.95, 47.47) | 0.22 (0.2, 0.23) | 237.54 (178.26, 307.07) | 0.46 (0.34, 0.59) | 111.23 (56.16, 174.46) |
| **Comoros** | 0.06 (0.02, 0.09) | 0.02 (0.01, 0.03) | 0.1 (0.06, 0.15) | 0.02 (0.01, 0.03) | -3.71 (-39.62, 116.22) |
| **Congo** | 0.4 (0.28, 0.57) | 0.03 (0.02, 0.04) | 0.87 (0.57, 1.26) | 0.02 (0.02, 0.04) | -8.84 (-47.32, 44.64) |
| **Cook Islands** | 0.04 (0.03, 0.05) | 0.3 (0.23, 0.38) | 0.03 (0.02, 0.05) | 0.13 (0.09, 0.18) | -56.5 (-69.88, -36.74) |
| **Costa Rica** | 3.2 (2.96, 3.47) | 0.16 (0.15, 0.18) | 52.43 (39.49, 67.64) | 1.02 (0.77, 1.31) | 520.29 (355.22, 712.65) |
| **Croatia** | 27.85 (25.45, 30.43) | 0.47 (0.43, 0.51) | 43.06 (32.68, 55.3) | 0.59 (0.45, 0.76) | 24.95 (-7.53, 63.25) |
| **Cuba** | 17.27 (16.09, 18.6) | 0.17 (0.16, 0.18) | 114.05 (90.92, 141.47) | 0.64 (0.51, 0.79) | 278.06 (194.75, 377.65) |
| **Cyprus** | 5.21 (4.12, 6.66) | 0.6 (0.49, 0.77) | 15.69 (12.91, 19.1) | 0.8 (0.66, 0.97) | 31.91 (-4.87, 82.06) |
| **Czechia** | 54.88 (51.52, 58.69) | 0.44 (0.41, 0.47) | 90.57 (73.6, 110.08) | 0.49 (0.39, 0.6) | 10.58 (-11.79, 37.45) |
| **Côte d'Ivoire** | 4.58 (3.05, 7.15) | 0.08 (0.05, 0.13) | 6.3 (4.23, 8.81) | 0.04 (0.03, 0.06) | -47.61 (-68.83, -12.27) |
| **Democratic People's Republic of Korea** | 40.96 (26.45, 63.02) | 0.18 (0.12, 0.27) | 43.49 (28.09, 69.15) | 0.14 (0.09, 0.21) | -25.21 (-54.75, 19.34) |
| **Democratic Republic of the Congo** | 4.96 (3.34, 8.44) | 0.02 (0.01, 0.04) | 9.22 (6.15, 13.77) | 0.02 (0.01, 0.03) | -14.63 (-46.59, 32.6) |
| **Denmark** | 102.98 (94.17, 112.48) | 1.3 (1.2, 1.42) | 167.51 (148.65, 188.15) | 1.47 (1.31, 1.64) | 12.83 (-2.76, 29.37) |
| **Djibouti** | 0.05 (0.03, 0.08) | 0.02 (0.01, 0.03) | 0.17 (0.1, 0.27) | 0.02 (0.01, 0.03) | 4.65 (-34.05, 67.1) |
| **Dominica** | 0.13 (0.11, 0.15) | 0.19 (0.16, 0.23) | 0.65 (0.47, 0.83) | 0.77 (0.56, 1) | 297.63 (182.58, 453.65) |
| **Dominican Republic** | 8.74 (7.19, 10.49) | 0.18 (0.15, 0.22) | 37.83 (23.15, 59.09) | 0.39 (0.24, 0.6) | 111.31 (27.94, 242.58) |
| **Ecuador** | 9.23 (8.45, 10.1) | 0.15 (0.14, 0.17) | 62.4 (47.39, 80.72) | 0.4 (0.31, 0.52) | 168.83 (103.71, 254.34) |
| **Egypt** | 45.6 (37.72, 54.34) | 0.13 (0.1, 0.15) | 176.73 (97.52, 284.43) | 0.24 (0.13, 0.38) | 85.01 (-4.87, 213.51) |
| **El Salvador** | 4.13 (3.69, 4.63) | 0.12 (0.11, 0.14) | 6.43 (4.56, 8.7) | 0.11 (0.08, 0.15) | -9.39 (-37.05, 23.97) |
| **Equatorial Guinea** | 0.05 (0.03, 0.09) | 0.02 (0.01, 0.03) | 0.18 (0.1, 0.28) | 0.03 (0.01, 0.04) | 44.16 (-28.25, 184.82) |
| **Eritrea** | 0.3 (0.2, 0.47) | 0.02 (0.01, 0.03) | 0.84 (0.57, 1.23) | 0.02 (0.01, 0.03) | 7.57 (-28.23, 66.73) |
| **Estonia** | 2.94 (2.6, 3.58) | 0.15 (0.13, 0.18) | 2.02 (1.56, 2.58) | 0.09 (0.07, 0.12) | -37.73 (-54.1, -18.53) |
| **Eswatini** | 0.27 (0.18, 0.39) | 0.07 (0.05, 0.11) | 0.45 (0.3, 0.64) | 0.06 (0.04, 0.09) | -11.63 (-49.61, 65.16) |
| **Ethiopia** | 6.76 (4.52, 11.33) | 0.02 (0.01, 0.04) | 10.88 (7.84, 15.07) | 0.02 (0.01, 0.02) | -26.37 (-48.88, 1.4) |
| **Fiji** | 0.36 (0.26, 0.48) | 0.08 (0.06, 0.11) | 0.71 (0.48, 0.99) | 0.08 (0.06, 0.12) | 0.68 (-39.24, 63.75) |
| **Finland** | 127.51 (119.41, 135.56) | 1.82 (1.71, 1.93) | 239.66 (206.14, 274.56) | 2.07 (1.8, 2.36) | 13.53 (-2.74, 32.87) |
| **France** | 1003.61 (956.39, 1049.51) | 1.3 (1.24, 1.35) | 1938.2 (1690.07, 2168.32) | 1.53 (1.35, 1.71) | 18.08 (4.25, 31.99) |
| **Gabon** | 0.2 (0.13, 0.34) | 0.03 (0.02, 0.05) | 0.35 (0.23, 0.53) | 0.03 (0.02, 0.04) | -3.59 (-47.48, 58.37) |
| **Gambia** | 0.28 (0.17, 0.43) | 0.06 (0.04, 0.1) | 0.54 (0.38, 0.71) | 0.04 (0.03, 0.06) | -26.67 (-59.64, 26.83) |
| **Georgia** | 5.03 (3.82, 8.09) | 0.08 (0.06, 0.13) | 5.87 (4.84, 7) | 0.11 (0.09, 0.13) | 36.92 (-14.59, 89.78) |
| **Germany** | 1271.45 (1216, 1323.18) | 1.11 (1.06, 1.15) | 2213.48 (2021.93, 2421.62) | 1.27 (1.17, 1.36) | 14.24 (5.38, 24.38) |
| **Ghana** | 5.63 (4.12, 7.58) | 0.07 (0.05, 0.1) | 15.97 (11.39, 22.06) | 0.08 (0.06, 0.11) | 14.92 (-30.31, 92.37) |
| **Greece** | 34.8 (32.37, 37.31) | 0.26 (0.24, 0.29) | 191.29 (166.9, 216.31) | 1.01 (0.88, 1.14) | 289.62 (234.56, 348.5) |
| **Greenland** | 0.13 (0.11, 0.16) | 0.36 (0.3, 0.43) | 0.26 (0.2, 0.33) | 0.38 (0.29, 0.47) | 5.98 (-21.41, 40.81) |
| **Grenada** | 0.1 (0.09, 0.12) | 0.16 (0.14, 0.18) | 0.67 (0.57, 0.76) | 0.58 (0.5, 0.66) | 266.57 (203.74, 329.15) |
| **Guam** | 0.73 (0.56, 0.93) | 0.9 (0.69, 1.13) | 0.12 (0.09, 0.17) | 0.06 (0.05, 0.08) | -93.05 (-95.22, -89.93) |
| **Guatemala** | 6.86 (6.01, 7.8) | 0.15 (0.13, 0.17) | 16.18 (12.52, 20.78) | 0.14 (0.11, 0.18) | -3.19 (-27.14, 29.57) |
| **Guinea** | 2.71 (1.72, 4.07) | 0.07 (0.04, 0.11) | 3.02 (2.16, 4.24) | 0.04 (0.03, 0.06) | -38.63 (-61.16, 2.01) |
| **Guinea-Bissau** | 0.47 (0.32, 0.68) | 0.09 (0.06, 0.14) | 0.5 (0.35, 0.68) | 0.05 (0.04, 0.07) | -45.74 (-65.7, -13.18) |
| **Guyana** | 0.68 (0.58, 0.79) | 0.15 (0.13, 0.17) | 3.06 (2.32, 3.93) | 0.45 (0.34, 0.57) | 195.53 (121.5, 291.75) |
| **Haiti** | 12.25 (8.51, 18.23) | 0.24 (0.17, 0.35) | 30.25 (19.73, 43.91) | 0.35 (0.22, 0.52) | 43.14 (-9.32, 120.86) |
| **Honduras** | 9.35 (7.15, 12.15) | 0.31 (0.24, 0.38) | 37.87 (22.53, 56.4) | 0.57 (0.33, 0.85) | 83.94 (10.35, 175.5) |
| **Hungary** | 63.71 (59.86, 67.54) | 0.52 (0.48, 0.55) | 95.05 (76.29, 116.89) | 0.59 (0.47, 0.73) | 13.74 (-9.22, 39.15) |
| **Iceland** | 3.42 (3.13, 3.69) | 1.22 (1.12, 1.32) | 7.24 (6.23, 8.22) | 1.38 (1.19, 1.57) | 12.66 (-3.4, 31.4) |
| **India** | 467.42 (338.36, 624.71) | 0.08 (0.06, 0.12) | 1598.48 (1225.17, 1994.54) | 0.13 (0.1, 0.16) | 55.75 (17.98, 105.28) |
| **Indonesia** | 98.49 (74.88, 127.78) | 0.08 (0.06, 0.1) | 239.7 (159.88, 338.76) | 0.1 (0.07, 0.14) | 22.82 (-10.16, 56.99) |
| **Iran (Islamic Republic of)** | 28.1 (21.18, 39.68) | 0.08 (0.06, 0.12) | 103.49 (89.25, 119.28) | 0.14 (0.12, 0.16) | 61.92 (3.97, 133.98) |
| **Iraq** | 3.21 (2.29, 4.7) | 0.03 (0.02, 0.05) | 10.94 (7.71, 14.99) | 0.04 (0.03, 0.05) | 19.75 (-35.81, 111.1) |
| **Ireland** | 60.36 (56.6, 64.29) | 1.49 (1.4, 1.58) | 168.68 (148.7, 188.28) | 2.3 (2.03, 2.55) | 54.39 (35.9, 73.67) |
| **Israel** | 39.75 (36.91, 42.79) | 0.82 (0.76, 0.88) | 101.19 (91.63, 111.29) | 0.91 (0.83, 1) | 12.2 (0.31, 25.24) |
| **Italy** | 704.86 (686.05, 721.61) | 0.9 (0.87, 0.93) | 1780.77 (1558.88, 1946.65) | 1.36 (1.21, 1.49) | 51.84 (37.05, 65.85) |
| **Jamaica** | 2.7 (2.49, 2.91) | 0.15 (0.14, 0.17) | 10.66 (7.97, 13.73) | 0.37 (0.27, 0.48) | 141.27 (78.2, 212.39) |
| **Japan** | 996.67 (959.44, 1019.99) | 0.59 (0.57, 0.6) | 2500.11 (2164.81, 2746.31) | 0.74 (0.66, 0.8) | 25.71 (14.59, 35.62) |
| **Jordan** | 1.02 (0.78, 1.33) | 0.05 (0.04, 0.07) | 5.69 (4.36, 7.43) | 0.06 (0.05, 0.08) | 21.1 (-22.72, 91.79) |
| **Kazakhstan** | 1.86 (1.44, 2.42) | 0.01 (0.01, 0.02) | 4.62 (3.9, 5.38) | 0.02 (0.02, 0.03) | 71.12 (23.26, 131.1) |
| **Kenya** | 2.36 (1.76, 3.2) | 0.02 (0.01, 0.03) | 6.58 (4.98, 8.57) | 0.02 (0.01, 0.03) | 18.87 (-16.95, 71.95) |
| **Kiribati** | 0.22 (0.16, 0.29) | 0.54 (0.4, 0.7) | 0.4 (0.27, 0.59) | 0.56 (0.38, 0.84) | 4.95 (-34.08, 68.2) |
| **Kuwait** | 3.73 (3.02, 4.73) | 0.32 (0.26, 0.39) | 4.19 (2.97, 5.33) | 0.15 (0.1, 0.19) | -54.35 (-66.77, -38.72) |
| **Kyrgyzstan** | 1.93 (1.5, 3.13) | 0.06 (0.05, 0.1) | 4.26 (3.68, 4.94) | 0.07 (0.06, 0.09) | 25.12 (-24.77, 76.62) |
| **Lao People's Democratic Republic** | 2.11 (1.49, 3.13) | 0.08 (0.05, 0.11) | 3.71 (2.36, 5.4) | 0.07 (0.04, 0.1) | -8.94 (-41.73, 35.29) |
| **Latvia** | 1.98 (1.73, 2.64) | 0.06 (0.05, 0.07) | 8.81 (7.16, 10.78) | 0.26 (0.21, 0.31) | 356.89 (224.28, 492.24) |
| **Lebanon** | 2.06 (1.35, 3.39) | 0.08 (0.05, 0.13) | 4.35 (2.44, 7.54) | 0.08 (0.05, 0.15) | 7.29 (-39.31, 75.91) |
| **Lesotho** | 0.63 (0.39, 1.03) | 0.05 (0.03, 0.09) | 0.93 (0.62, 1.37) | 0.06 (0.04, 0.09) | 14.17 (-36.66, 108.7) |
| **Liberia** | 0.97 (0.66, 1.34) | 0.07 (0.05, 0.11) | 1.09 (0.7, 1.65) | 0.04 (0.03, 0.06) | -46.44 (-69.78, -7.96) |
| **Libya** | 1.48 (1.04, 2.21) | 0.06 (0.04, 0.1) | 5.89 (3.15, 9.09) | 0.1 (0.05, 0.15) | 51.41 (-30.88, 186.97) |
| **Lithuania** | 8.5 (7.33, 10.89) | 0.19 (0.17, 0.25) | 36.22 (28.74, 44.92) | 0.71 (0.57, 0.87) | 265.18 (163.85, 394.62) |
| **Luxembourg** | 5.83 (5.36, 6.33) | 1.12 (1.03, 1.21) | 9.92 (8.44, 11.47) | 1.05 (0.89, 1.22) | -6.46 (-20.66, 10.32) |
| **Madagascar** | 1.64 (1.14, 2.33) | 0.02 (0.01, 0.03) | 2.87 (2.02, 3.78) | 0.02 (0.01, 0.02) | -18.33 (-46.04, 21.87) |
| **Malawi** | 1.18 (0.81, 1.73) | 0.02 (0.01, 0.03) | 2.09 (1.45, 2.89) | 0.02 (0.01, 0.03) | -8.04 (-36.26, 32.54) |
| **Malaysia** | 5.52 (4.37, 6.91) | 0.05 (0.04, 0.06) | 13.02 (9.35, 18) | 0.04 (0.03, 0.06) | -13.57 (-43.91, 31.07) |
| **Maldives** | 0.18 (0.11, 0.3) | 0.13 (0.09, 0.22) | 0.54 (0.41, 0.72) | 0.16 (0.12, 0.21) | 21.93 (-36.16, 106.8) |
| **Mali** | 3.59 (2.25, 5.56) | 0.07 (0.04, 0.11) | 4.46 (2.94, 6.41) | 0.04 (0.02, 0.06) | -45.96 (-64.57, -18.85) |
| **Malta** | 4.08 (3.77, 4.4) | 0.97 (0.89, 1.04) | 10 (8.49, 11.55) | 1.17 (1, 1.36) | 21.48 (1.72, 43.67) |
| **Marshall Islands** | 0.04 (0.03, 0.06) | 0.24 (0.17, 0.34) | 0.06 (0.04, 0.08) | 0.15 (0.1, 0.2) | -40.16 (-62.98, -5.16) |
| **Mauritania** | 1.18 (0.87, 1.58) | 0.1 (0.07, 0.14) | 1.17 (0.73, 1.67) | 0.05 (0.03, 0.07) | -54.47 (-72.29, -28.22) |
| **Mauritius** | 1.48 (1.38, 1.6) | 0.18 (0.17, 0.19) | 4.03 (3.17, 5.04) | 0.23 (0.18, 0.29) | 31.23 (3.01, 64.95) |
| **Mexico** | 114.82 (110.75, 119.09) | 0.22 (0.21, 0.23) | 501.04 (417.55, 584.14) | 0.41 (0.35, 0.48) | 89.57 (57.07, 122.21) |
| **Micronesia (Federated States of)** | 0.12 (0.08, 0.17) | 0.23 (0.16, 0.33) | 0.13 (0.08, 0.18) | 0.15 (0.1, 0.21) | -37.03 (-63.33, 8.2) |
| **Monaco** | 0.15 (0.11, 0.2) | 0.27 (0.2, 0.36) | 0.3 (0.21, 0.41) | 0.34 (0.25, 0.46) | 25.99 (-14.3, 84.56) |
| **Mongolia** | 2.48 (1.81, 3.67) | 0.16 (0.12, 0.21) | 5.05 (3.46, 7.2) | 0.18 (0.12, 0.26) | 12.28 (-30.7, 78.93) |
| **Montenegro** | 0.31 (0.25, 0.38) | 0.05 (0.04, 0.06) | 0.49 (0.38, 0.61) | 0.05 (0.04, 0.06) | 9.08 (-19.92, 48.98) |
| **Morocco** | 7.43 (5.45, 10.44) | 0.05 (0.03, 0.07) | 21.8 (14.97, 30.08) | 0.06 (0.04, 0.08) | 35.02 (-15.54, 101.15) |
| **Mozambique** | 1.53 (0.96, 2.61) | 0.02 (0.01, 0.03) | 3.64 (2.52, 5.17) | 0.02 (0.01, 0.03) | 8.14 (-27.53, 69.44) |
| **Myanmar** | 23.57 (16.8, 33.75) | 0.08 (0.06, 0.12) | 40.19 (26.43, 57.59) | 0.08 (0.05, 0.11) | -4.76 (-38.76, 47.39) |
| **Namibia** | 0.56 (0.38, 0.78) | 0.07 (0.05, 0.1) | 0.91 (0.64, 1.27) | 0.06 (0.04, 0.08) | -19.16 (-50.38, 33.45) |
| **Nauru** | 0.01 (0.01, 0.02) | 0.3 (0.2, 0.47) | 0.01 (0.01, 0.01) | 0.16 (0.11, 0.23) | -45.85 (-66.49, -18.34) |
| **Nepal** | 7.73 (4.49, 13.19) | 0.06 (0.03, 0.12) | 23.71 (13.54, 40.88) | 0.1 (0.06, 0.17) | 55.69 (2.13, 147.03) |
| **Netherlands** | 293 (277.5, 307.93) | 1.53 (1.45, 1.61) | 597.77 (537.38, 657.54) | 1.82 (1.65, 2) | 18.99 (7.46, 31.13) |
| **New Zealand** | 67.3 (62.83, 71.85) | 1.72 (1.61, 1.83) | 157.12 (141.66, 172.01) | 2.07 (1.88, 2.25) | 20.62 (9.49, 34.04) |
| **Nicaragua** | 2.02 (1.68, 2.44) | 0.11 (0.09, 0.13) | 5.64 (4.26, 7.44) | 0.12 (0.09, 0.16) | 12.27 (-23.06, 57.89) |
| **Niger** | 2.35 (1.37, 3.95) | 0.06 (0.03, 0.11) | 3.46 (2.17, 5.4) | 0.03 (0.02, 0.05) | -53.09 (-66.94, -27.62) |
| **Nigeria** | 34.54 (23.98, 46.18) | 0.07 (0.04, 0.09) | 53.98 (36.94, 76.01) | 0.05 (0.03, 0.07) | -27.28 (-57.06, 16.45) |
| **Niue** | 0.01 (0, 0.01) | 0.3 (0.21, 0.43) | 0 (0, 0.01) | 0.18 (0.13, 0.24) | -40.92 (-62.8, -4.01) |
| **North Macedonia** | 4.38 (3.73, 5.47) | 0.23 (0.19, 0.29) | 1.91 (1.39, 2.46) | 0.06 (0.04, 0.08) | -73.76 (-81.56, -63.82) |
| **Northern Mariana Islands** | 0.03 (0.02, 0.04) | 0.13 (0.1, 0.18) | 0.04 (0.03, 0.05) | 0.05 (0.04, 0.07) | -59.03 (-72.25, -39.43) |
| **Norway** | 98.01 (93.85, 101.14) | 1.51 (1.46, 1.56) | 147.17 (132.74, 161.48) | 1.58 (1.43, 1.73) | 4.55 (-4.87, 14.89) |
| **Oman** | 0.61 (0.4, 1.22) | 0.07 (0.04, 0.14) | 2.04 (1.1, 2.96) | 0.09 (0.04, 0.13) | 25.18 (-37.31, 123.4) |
| **Pakistan** | 55.88 (35.34, 83.35) | 0.08 (0.05, 0.13) | 187.9 (143.11, 247.55) | 0.14 (0.1, 0.18) | 69.06 (13.66, 168.68) |
| **Palau** | 0 (0, 0.01) | 0.04 (0.03, 0.05) | 0.01 (0.01, 0.01) | 0.04 (0.03, 0.05) | 7.88 (-33.74, 77.52) |
| **Palestine** | 0.35 (0.24, 0.53) | 0.03 (0.02, 0.04) | 1.36 (1.07, 1.7) | 0.04 (0.03, 0.05) | 50.56 (-15.54, 165.25) |
| **Panama** | 3 (2.75, 3.25) | 0.18 (0.17, 0.2) | 11.99 (9.02, 15.58) | 0.29 (0.22, 0.38) | 60.09 (19.72, 108.13) |
| **Papua New Guinea** | 2.2 (1.08, 3.71) | 0.1 (0.05, 0.17) | 3.48 (1.78, 5.97) | 0.06 (0.03, 0.11) | -39.34 (-59.36, -9.15) |
| **Paraguay** | 3.19 (2.63, 3.82) | 0.12 (0.1, 0.15) | 23.07 (16.25, 32.09) | 0.39 (0.28, 0.55) | 217.38 (106.64, 381.88) |
| **Peru** | 20.2 (16.52, 24.62) | 0.15 (0.12, 0.18) | 55.19 (37.92, 78.97) | 0.17 (0.12, 0.24) | 17.02 (-23.21, 75.81) |
| **Philippines** | 23.77 (19.68, 27.47) | 0.06 (0.05, 0.07) | 64.7 (52.91, 78.07) | 0.07 (0.06, 0.09) | 17.22 (-13.88, 60.98) |
| **Poland** | 104.79 (101.46, 108.53) | 0.26 (0.25, 0.27) | 422.95 (355, 500.88) | 0.68 (0.57, 0.8) | 158.24 (118.06, 205.13) |
| **Portugal** | 65.22 (61.15, 69.24) | 0.53 (0.5, 0.57) | 244.23 (216.5, 270.98) | 1.19 (1.06, 1.32) | 123.1 (92.48, 153.33) |
| **Puerto Rico** | 6.67 (6.22, 7.13) | 0.19 (0.17, 0.2) | 40.36 (30.27, 52.36) | 0.64 (0.48, 0.84) | 245.94 (158.77, 353) |
| **Qatar** | 0.1 (0.07, 0.14) | 0.05 (0.04, 0.08) | 0.78 (0.54, 1.08) | 0.05 (0.03, 0.07) | -4.2 (-45.99, 63.42) |
| **Republic of Korea** | 136.64 (118.21, 159.74) | 0.41 (0.35, 0.48) | 78.5 (67.9, 90.66) | 0.09 (0.08, 0.1) | -78.53 (-82.81, -73.74) |
| **Republic of Moldova** | 8.95 (7.95, 10.61) | 0.2 (0.18, 0.23) | 6.66 (5.67, 7.73) | 0.13 (0.11, 0.15) | -32.68 (-46.1, -17.63) |
| **Romania** | 73.14 (67.42, 79.24) | 0.32 (0.29, 0.36) | 60.97 (49.36, 74.22) | 0.22 (0.18, 0.26) | -33.17 (-46.89, -17.87) |
| **Russian Federation** | 188.68 (162.03, 261.1) | 0.11 (0.09, 0.15) | 742.01 (636.66, 848.59) | 0.33 (0.28, 0.37) | 205.61 (110.24, 289.33) |
| **Rwanda** | 1.01 (0.69, 1.5) | 0.02 (0.01, 0.04) | 1.73 (1.15, 2.6) | 0.02 (0.01, 0.03) | -17.93 (-44.62, 19.99) |
| **Saint Kitts and Nevis** | 0.06 (0.06, 0.07) | 0.18 (0.16, 0.2) | 0.48 (0.37, 0.6) | 0.69 (0.54, 0.85) | 289.84 (199.39, 393.47) |
| **Saint Lucia** | 0.16 (0.15, 0.18) | 0.18 (0.16, 0.19) | 1.45 (1.18, 1.75) | 0.68 (0.56, 0.83) | 288.54 (208.29, 380.45) |
| **Saint Vincent and the Grenadines** | 0.14 (0.13, 0.15) | 0.18 (0.16, 0.2) | 0.68 (0.58, 0.8) | 0.51 (0.43, 0.6) | 181.06 (131.9, 239.41) |
| **Samoa** | 0.24 (0.14, 0.36) | 0.25 (0.15, 0.38) | 0.2 (0.13, 0.28) | 0.12 (0.08, 0.17) | -51.18 (-68.21, -23.3) |
| **San Marino** | 0.03 (0.02, 0.04) | 0.1 (0.08, 0.13) | 0.06 (0.04, 0.09) | 0.11 (0.06, 0.16) | 4.27 (-40.31, 73.92) |
| **Sao Tome and Principe** | 0.21 (0.15, 0.27) | 0.25 (0.18, 0.33) | 0.6 (0.35, 0.88) | 0.48 (0.27, 0.7) | 90.65 (6.64, 212.84) |
| **Saudi Arabia** | 5.14 (2.72, 8.74) | 0.07 (0.03, 0.12) | 19.91 (13.39, 28.96) | 0.09 (0.06, 0.12) | 23.95 (-40.7, 186.83) |
| **Senegal** | 3.13 (2.01, 4.75) | 0.08 (0.05, 0.12) | 4.22 (2.86, 5.87) | 0.05 (0.03, 0.06) | -42.69 (-64.26, -6.89) |
| **Serbia** | 7.58 (6.26, 9.26) | 0.06 (0.05, 0.08) | 9.02 (6.76, 11.65) | 0.06 (0.05, 0.08) | -1.75 (-32.09, 36.8) |
| **Seychelles** | 0.14 (0.12, 0.17) | 0.25 (0.2, 0.31) | 0.37 (0.22, 0.56) | 0.31 (0.19, 0.47) | 24.96 (-20.55, 88.85) |
| **Sierra Leone** | 1.41 (0.94, 2.02) | 0.06 (0.04, 0.09) | 1.8 (1.25, 2.52) | 0.04 (0.03, 0.05) | -39.25 (-63.46, -3.52) |
| **Singapore** | 8.53 (7.94, 9.21) | 0.36 (0.33, 0.39) | 27.28 (23.23, 31.52) | 0.37 (0.31, 0.42) | 2.39 (-15.09, 20.47) |
| **Slovakia** | 6.16 (5.34, 7.17) | 0.1 (0.09, 0.12) | 14.22 (10.7, 18.83) | 0.16 (0.12, 0.21) | 52.84 (12.39, 109.01) |
| **Slovenia** | 10.27 (7.92, 13.34) | 0.47 (0.37, 0.59) | 1.1 (0.82, 1.48) | 0.03 (0.02, 0.04) | -93.53 (-95.66, -90.69) |
| **Solomon Islands** | 0.29 (0.17, 0.46) | 0.18 (0.11, 0.29) | 0.39 (0.26, 0.54) | 0.1 (0.07, 0.15) | -41.07 (-61.85, -6.56) |
| **Somalia** | 0.61 (0.38, 1.01) | 0.01 (0.01, 0.03) | 1.61 (1.04, 2.38) | 0.01 (0.01, 0.02) | -19.62 (-42.76, 17.25) |
| **South Africa** | 12.34 (10.67, 14.13) | 0.05 (0.04, 0.06) | 17.31 (15.46, 19.12) | 0.03 (0.03, 0.04) | -31.34 (-42.18, -16.8) |
| **South Sudan** | 0.72 (0.41, 1.5) | 0.02 (0.01, 0.04) | 0.89 (0.58, 1.37) | 0.01 (0.01, 0.02) | -27.35 (-53.97, 9.39) |
| **Spain** | 433.74 (414.44, 452.81) | 0.86 (0.83, 0.9) | 1118.45 (988.85, 1243.63) | 1.29 (1.14, 1.43) | 49.78 (32.96, 66.72) |
| **Sri Lanka** | 32.11 (26.33, 39.36) | 0.25 (0.21, 0.31) | 29.52 (20.06, 41.84) | 0.11 (0.08, 0.16) | -55.5 (-71.25, -35.7) |
| **Sudan** | 4.77 (3.14, 7.6) | 0.04 (0.03, 0.07) | 11.82 (7.87, 17.35) | 0.05 (0.03, 0.08) | 21.58 (-27.9, 102.92) |
| **Suriname** | 0.5 (0.42, 0.58) | 0.17 (0.15, 0.2) | 2.62 (1.97, 3.5) | 0.43 (0.33, 0.57) | 150.5 (82.49, 242.71) |
| **Sweden** | 192.35 (181.22, 202.96) | 1.3 (1.24, 1.37) | 358.8 (323.35, 390.85) | 1.74 (1.59, 1.88) | 33.4 (21.93, 44.91) |
| **Switzerland** | 146.14 (132.16, 160.58) | 1.43 (1.3, 1.55) | 222.57 (198.33, 246.42) | 1.33 (1.19, 1.46) | -7.03 (-18.91, 5.57) |
| **Syrian Arab Republic** | 3.57 (2.59, 4.93) | 0.05 (0.04, 0.08) | 10.25 (5.78, 17.45) | 0.07 (0.04, 0.12) | 34.68 (-25.77, 136.76) |
| **Taiwan (Province of China)** | 44.43 (41.96, 47.15) | 0.25 (0.23, 0.26) | 150.97 (112.98, 200.3) | 0.42 (0.32, 0.55) | 69.4 (26.29, 126.8) |
| **Tajikistan** | 0.91 (0.68, 1.24) | 0.03 (0.02, 0.04) | 2.42 (1.79, 3.19) | 0.04 (0.03, 0.05) | 37.23 (-14.1, 119.14) |
| **Thailand** | 20.63 (16.65, 25.65) | 0.05 (0.04, 0.06) | 32.68 (22.42, 45.63) | 0.03 (0.02, 0.04) | -34.52 (-57.07, -1.83) |
| **Timor-Leste** | 0.23 (0.14, 0.36) | 0.05 (0.03, 0.08) | 0.59 (0.31, 0.91) | 0.06 (0.03, 0.1) | 23.8 (-26.77, 99.05) |
| **Togo** | 1.23 (0.92, 1.63) | 0.08 (0.05, 0.1) | 2.19 (1.47, 3.1) | 0.05 (0.03, 0.07) | -39.92 (-64.14, 0.08) |
| **Tokelau** | 0 (0, 0) | 0.23 (0.15, 0.33) | 0 (0, 0) | 0.13 (0.09, 0.18) | -44.25 (-64.52, -12.54) |
| **Tonga** | 0.11 (0.07, 0.16) | 0.18 (0.11, 0.27) | 0.09 (0.05, 0.13) | 0.11 (0.07, 0.16) | -39.01 (-59.48, -7.87) |
| **Trinidad and Tobago** | 1.32 (1.22, 1.41) | 0.15 (0.14, 0.16) | 7.21 (5.22, 9.68) | 0.38 (0.28, 0.51) | 160.54 (86.32, 252.77) |
| **Tunisia** | 3.05 (2.23, 4.21) | 0.05 (0.04, 0.07) | 8.5 (5.53, 12.61) | 0.06 (0.04, 0.09) | 25.28 (-30, 119.52) |
| **Turkey** | 431.47 (291.04, 702.18) | 0.79 (0.58, 1.16) | 623.75 (480.58, 788.94) | 0.76 (0.59, 0.95) | -4.73 (-38.71, 39.24) |
| **Turkmenistan** | 2.7 (2.4, 3.01) | 0.12 (0.1, 0.13) | 9.7 (6.97, 12.88) | 0.21 (0.16, 0.28) | 83.93 (32.28, 139.93) |
| **Tuvalu** | 0.02 (0.01, 0.03) | 0.24 (0.15, 0.33) | 0.01 (0.01, 0.02) | 0.12 (0.08, 0.18) | -48.55 (-66.36, -21.05) |
| **Uganda** | 1.67 (0.98, 2.93) | 0.02 (0.01, 0.03) | 4.4 (3.05, 6.34) | 0.02 (0.01, 0.03) | 6.09 (-33.52, 79.46) |
| **Ukraine** | 77.78 (62.45, 111.15) | 0.12 (0.1, 0.16) | 148.84 (123.15, 177.69) | 0.24 (0.2, 0.28) | 102.9 (34.41, 170.66) |
| **United Arab Emirates** | 0.99 (0.59, 2.48) | 0.13 (0.07, 0.35) | 12.71 (4.81, 25.78) | 0.17 (0.06, 0.34) | 24.95 (-49.26, 143.62) |
| **United Kingdom** | 1294.62 (1252.41, 1323.42) | 1.5 (1.46, 1.53) | 2543.42 (2344, 2699.38) | 2.06 (1.91, 2.18) | 37.22 (27.78, 45.89) |
| **United Republic of Tanzania** | 3.52 (2.43, 5.23) | 0.02 (0.01, 0.03) | 7.92 (5.76, 10.65) | 0.02 (0.01, 0.03) | -3.3 (-32.41, 38.94) |
| **United States of America** | 3730.05 (3606.83, 3829.81) | 1.21 (1.17, 1.24) | 9494.62 (8998.44, 9809.22) | 1.76 (1.68, 1.82) | 45.8 (40.63, 50.88) |
| **United States Virgin Islands** | 0.35 (0.28, 0.44) | 0.39 (0.31, 0.49) | 2.04 (1.46, 2.71) | 1.12 (0.82, 1.44) | 187.1 (97.21, 307.23) |
| **Uruguay** | 8.21 (7.67, 8.78) | 0.22 (0.21, 0.24) | 54.02 (48.99, 59.01) | 1.07 (0.97, 1.16) | 384.02 (330.93, 435.61) |
| **Uzbekistan** | 2.59 (2.19, 3.72) | 0.02 (0.02, 0.03) | 9.66 (7.79, 11.71) | 0.04 (0.03, 0.04) | 71.95 (13.55, 125.38) |
| **Vanuatu** | 0.12 (0.06, 0.2) | 0.16 (0.09, 0.29) | 0.19 (0.12, 0.31) | 0.1 (0.06, 0.16) | -37.01 (-58.74, 3) |
| **Venezuela (Bolivarian Republic of)** | 18.04 (16.65, 19.42) | 0.16 (0.15, 0.17) | 118.49 (84.49, 158.58) | 0.4 (0.29, 0.53) | 152.68 (76.01, 240.75) |
| **Viet Nam** | 36.16 (24.58, 53.2) | 0.08 (0.05, 0.12) | 104.77 (74.13, 141.53) | 0.1 (0.07, 0.14) | 27.97 (-25.14, 109.54) |
| **Yemen** | 2.27 (1.36, 3.93) | 0.03 (0.02, 0.06) | 7.35 (5.08, 10.63) | 0.04 (0.03, 0.06) | 24.02 (-31.23, 125.36) |
| **Zambia** | 1.26 (0.94, 1.72) | 0.02 (0.02, 0.04) | 2.78 (2.08, 3.59) | 0.02 (0.02, 0.03) | -0.66 (-35.23, 51.83) |
| **Zimbabwe** | 7.41 (5.78, 9.27) | 0.15 (0.12, 0.19) | 16.74 (10.22, 26.44) | 0.2 (0.12, 0.31) | 30.67 (-27.59, 131.27) |

Legend. Data in parentheses are 95% uncertainty intervals (UI). MND=motor neuron disease, DALY=disability-adjusted life-year, YLD= years lived with disability, YLL=years of life lost

2. Supplementary material.

*Disability-adjusted life-years (DALYs), years lived with disability (YLDs), and years of life lost (YLLs)*

The GBD of MNDs was evaluated as their incidence, prevalence, related deaths, DALYs, YLDs, and YLLs.^1^ DALYs are defined as the sum of YLDs and YLLs. YLD refers to the individual sequelae prevalence of each disease multiplied by the disability weight, quantifying the severity of the sequelae as a number between 0 (indicating full health) and 1 (indicating death).^2^ YLL is the number of deaths multiplied by the standard life expectancy at the time of death. The standard life expectancy is obtained from the lowest observed age-specific mortality rate for a world population of over 5 million.^1^ Disability weights were estimated from nine US population surveys and an open internet survey that asked respondents to choose the healthier option among random pairs of health conditions provided with brief descriptions of key characteristics.^2^

1. Collaborators GCoD. Global, regional, and national age-sex specific mortality for 264 causes of death, 1980-2016: a systematic analysis for the Global Burden of Disease Study 2016. Lancet (London, England) 2017;390:1151-1210.

2. Salomon JA, Haagsma JA, Davis A, et al. Disability weights for the Global Burden of Disease 2013 study. The Lancet Global health 2015;3:e712-723.

## 3. Supplementary material.

## 2019 GBD of motor neuron diseases by region

High-income North America, Western Europe, Australasia, and Asia Pacific, as well as Southern Latin America had higher age-standardized prevalence rates in the following order: 8.86 (95% UI, 8.19-9.51), 8.33 (95% UI, 7.24-9.54), 8.03 (95% UI, 6.99-9.18), 4.96 (95% UI, 4.21-5.73), 4.77 (95% UI, 4.08-5.53). The age-standardized prevalence rates were low in the following regions: Oceania 1.40 (95% UI, 1.14-1.69), Central sub-Saharan Africa 1.42 (95% UI, 1.16-1.73), Western sub-Saharan Africa 1.49 (95% UI, 1.19-1.83), Eastern sub-Saharan Africa 1.49 (95% UI, 1.21-1.83), and Southeast Asia 1.70 (95% UI, 1.37-2.07).

The age-standardized incidence rates were high in Australasia, high-income North America, Western Europe, Southern Latin America, and high-income Asia Pacific—2.47 (95% UI, 2.36-2.58), 1.97 (95% UI, 1.88-2.06), 1.85 (95% UI, 1.73-1.95), 0.96 (95% UI, 0.87-1.06), 0.87 (95% UI, 0.78-0.97), respectively— and low in Southeast Asia, South Asia, Oceania, Andean Latin America, and Central Asia—0.40 (95% UI, 0.33-0.49), 0.42 (95% UI, 0.34-0.51), 0.43 (95% UI, 0.36-0.52), 0.45 (95% UI, 0.38-0.52), 0.49 (95% UI, 0.41-0.59), respectively.

The age-standardized DALY rates were high in Australasia, high-income North America, Western Europe, Southern Latin America, and Tropical Latin America—55.16 (95% UI, 50.13-60.39), 46.91 (95% UI, 45.25-48.37), 39.39 (95% UI, 36.6-41.89), 22.30 (95% UI, 20.38-23.94), 18.58 (95% UI, 16.98-19.87), respectively. Central sub-Saharan Africa, Eastern sub-Saharan Africa, Western sub-Saharan Africa, Southern sub-Saharan Africa, and Central Asia had lower age-standardized DALY rates—0.98 (95% UI, 0.76-1.27), 1.02 (95% UI, 0.81-1.25), 1.95 (95% UI, 1.59-2.41), 2.40 (95% UI, 1.99-2.98), 2.40 (95% UI, 2.1-2.75), respectively—than the remaining regions. The global age-standardized DALY rates of motor neuron diseases by age and sex are shown in Figure 2.

The age-standardized rates of deaths caused by MND showed a similar pattern as DALYs. Australasia, high-income North America, Western Europe, Southern Latin America, and Tropical Latin America were the top 5 regions with high age-standardized death rates. Central sub-Saharan Africa, Eastern sub-Saharan Africa, Western sub-Saharan Africa, Southern sub-Saharan Africa, and Central Asia had relatively low age-standardized death rates—0.02 (95% UI, 0.01-0.03), 0.02 (95% UI, 0.01-0.02), 0.05 (95% UI, 0.04-0.06), 0.06 (95% UI, 0.05-0.07), 0.06 (95% UI, 0.05-0.06), respectively.

## Regional trend of motor neuron disease between 1990 and 2019

Changes in the age-standardized prevalence rates between 1990 and 2019 were most prominent in Australasia (26.4% [95% UI, 19.56‒33.03]) and Western Europe (25.29% [95% UI, 22.14‒28.44]) but lowest in Oceania (0.75 [95% UI, -4.69‒0.84]) and Central sub-Saharan Africa (1.81 [95% UI, -1.69‒3.26]).

Changes in the age-standardized DALYs and death rates between 1990 and 2019 showed a similar pattern. The highest increase in the rates were observed in Southern Latin America—(DALY 170.04 [95% UI, 145.72‒193.32], death 242.23 [95% UI, 210.3‒271.37])—and the Caribbean—(DALY 123.77 [95% UI, 79.18‒179.23], death 179.6 [95% UI, 129.52‒239.82]). The lowest changes in the rates were observed in Oceania (-42.14 [95% UI, -54.72‒ -26.07] and -47.87 [95% UI, -59.78‒ -32.31]) and East Asia (-47.53 [95% UI, -55.99‒ -37.57] and -35.31 [95% UI, -46.51‒ -22.89]).

## 2019 GBD of motor neuron diseases by country

Prevalence, incidence, DALYs, YLDs, YLLs, and death due to MNDs by country in counts and age-standardized rates for both sexes for 2019 are listed in Supplementary Table. The age-standardized prevalence rates were high in Canada (11.98 [95% UI, 10.54‒13.61]), Andorra (11.71 [95% UI, 10.07‒13.44]), Finland (11.66 [95% UI, 10.01‒13.45]), Ireland (10.96 [95% UI, 9.5‒12.56]), and Sweden (10.95 [95% UI, 9.4‒12.73]). In contrast, Kiribati (1.17 [95% UI, 0.95‒1.42]), Somalia (1.22 [95% UI, 0.98‒1.5]), Burundi (1.27 [95% UI, 1.03‒1.55]), Central African Republic (1.27 [95% UI, 1.03‒1.54]), and Solomon Islands (1.29 [95% UI, 1.04‒1.55]) had lower age-standardized prevalence of MND than other countries.

In 2019, the age-standardized incidence of MND was low in Malaysia (0.35 [95% UI, 0.29‒0.43]), Seychelles (0.36 [95% UI, 0.3‒0.44]), Indonesia (0.37 [95% UI, 0.3‒0.45]), Maldives (0.37 [95% UI, 0.3‒0.45]), and Philippines (0.38 [95% UI, 0.32‒0.47]). In contrast, Ireland (2.53 [95% UI, 2.4‒2.67]), Finland (2.52 [95% UI, 2.35‒2.69]), Australia (2.48 [95% UI, 2.37‒2.59]), United Kingdom (2.47 [95% UI, 2.32‒2.61]), and Andorra (2.46 [95% UI, 2.24‒2.66]) had high age-standardized incidence of MND.

Age-standardized DALYs and death were high in the following countries: Ireland (DALY 58.75 [95% UI, 52.03‒64.85] and death 2.3 [95% UI, 2.03‒2.55]), Australia (55.54 [95% UI, 49.92‒61.27] and 2.14 [95% UI, 1.91‒2.37]), Andorra (54.46 [95% UI, 35.07‒81.63] and 2.1 [95% UI, 1.32‒3.14]), New Zealand (53.25 [95% UI, 49.11‒57.39] and 2.07 [95% UI, 1.88‒2.25]), and Finland (53.03 [95% UI, 46.88‒59.36] and 2.07 [95% UI, 1.8‒2.36]).

The age-standardized DALYs and death were low in the following countries: Somalia (DALY 0.75 [95% UI, 0.54‒1.05] and death 0.01 [95% UI, 0.01-0.02]), Central African Republic (0.84 [95% UI, 0.62‒1.13] and 0.01 [95% UI, 0.01-0.02]), Burundi (0.86 [95% UI, 0.62‒1.17] and 0.01 [95% UI, 0.01-0.02]), and Democratic Republic of the Congo South (0.92 [95% UI, 0.7‒1.26] and 0.02 [95% UI, 0.01-0.03]). The age-standardized death rates were low in Burundi, Central African Republic, Somalia, and South Sudan (0.01 [95% UI, 0.01‒0.02]).

## National trend of motor neuron disease between 1990 and 2019

Between 1990 and 2019, the DALYs rates were increased to the greatest extent in Barbados (411.19 [95% UI, -34.52‒34.05]), Costa Rica (367.3 [95% UI, -10.38‒50.84]), and Uruguay (262.8 [95% UI, -28.3‒36.31]). The DALYs rates decreased in the following countries: Slovenia (-90.88 [95% UI, -93.24‒ -87.91]), Guam (-89.75 [95% UI, -92.58‒ -85.68]), Bosnia and Herzegovina (-86.63 [95% UI, -89.83‒ -82.84]), and Republic of Korea (-85.22 [95% UI, -88.63‒ -81.2]). Portugal (38.09 [95% UI, 29.56‒47.99]), Italy (36.47 [95% UI, 30.12‒44.47]), Lithuania (57.88 [95% UI, 45.08‒70.86]), and Costa Rica (45.48 [95% UI, 39.81‒51.69]) showed the highest increase in age-standardized prevalence and incidence rates over the examined period. Sudan showed low DALY (0.92 [95% UI, 0.67‒1.24]) but an increased death rate of 21.58 (168.83 [95% UI, 103.71‒254.34]) in 2019 compared to 1990.
